# Ubiquitin-proteasome system regulates pro-crossover protein dynamics during meiosis in *Caenorhabditis elegans*

**DOI:** 10.1371/journal.pbio.3003868

**Published:** 2026-06-16

**Authors:** Hongtao Zhang, Wenqing Liang, Meng Li, Yuejun Yang, Lei He, Wencong Nan, Guoteng Liu, Bin Wang, Ye Hong

**Affiliations:** 1 Shandong Provincial Key Laboratory of Development and Regeneration, School of Life Sciences, Shandong University, Qingdao, Shandong, China; 2 Suzhou Research Institute of Shandong University, Suzhou, Jiangsu, China; 3 Shandong Cancer Hospital and Institute, Shandong First Medical University and Shandong Academy of Medical Sciences, Jinan, Shandong, China; 4 Institute of Biological Sciences and Technology, Guangxi Academy of Sciences, Nanning, Guangxi, China; Stowers Institute for Medical Research, UNITED STATES OF AMERICA

## Abstract

Crossover (CO) formation ensures accurate segregation of homologous chromosomes during the first meiotic division. The pro-crossover proteins are essential for crossover formation and undergo dynamic changes during meiotic prophase I, although the underlying regulatory mechanism is largely unknown. Here, we found that the ubiquitin-proteasome system (UPS) plays a pivotal role in orchestrating pro-crossover protein dynamics and crossover patterning during meiosis in *Caenorhabditis elegans*. Knockdown of either the ubiquitin-activating enzyme E1 or the proteasome resulted in elevated pro-crossover protein levels and crossover designation. Impairing ubiquitination, but not proteasome activity, led to persistent association of pro-crossover proteins on meiotic chromosomes, a process mediated by the CDC-48^UFD-1/NPL-4^ segregase. Utilizing a hypomorphic allele of *cosa-1*, a well-characterized pro-crossover protein-encoding gene, we further demonstrate that the UPS restricts crossover formation. Collectively, our findings reveal a multilayered UPS-mediated regulatory network that maintains proper pro-crossover protein dynamics, thereby coordinating crossover formation with meiotic chromosome segregation.

## Abstract 2

Crossover formation ensures accurate homologous chromosome segregation and depends on tightly regulated pro-crossover protein levels during meiotic prophase I. This study shows that the ubiquitin-proteasome system restricts crossover number by orchestrating pro-crossover protein dynamics and ensuring their timely dissociation from chromatin.

## Introduction

Accurate segregation of chromosomes into gametes depends critically on the establishment of crossovers (COs) between homologous chromosomes during the long prophase preceding the first meiotic division [1,2]. Meiotic crossover formation is a result of the repair of programmed DNA double-strand breaks (DSBs) via homologous recombination. At least two types of crossovers are known to exist in sexually reproducing multicellular organisms, referred to as class I and class II crossovers [3]. Class II crossovers are generally less abundant than class I, and are even absent in some organisms such as *Caenorhabditis elegans* [4]. An intriguing fact that distinguishes class I crossovers from class II crossovers is that they are subject to crossover interference (COI), a phenomenon in which the formation of one crossover prevents the formation of another crossover nearby on the same chromosome [5]. Although the mechanism of crossover interference is not yet fully understood, significant progress has been made in the field. Several factors have been identified that influence interference, including the synaptonemal complex (SC), topoisomerase II, and certain pro-crossover proteins such as HEI10 [6–10]. Based on existing evidence, two major models have been proposed to explain the regulation of crossover interference: the mechanical (or beam-film) model and the diffusion-based coarsening model [3,9,11,12]. The coarsening model posits that pro-crossover factors, which can diffuse along the SC/chromosomes, accumulate at a designated DSB site. This local aggregation sequesters additional factors from the surrounding region, thereby depleting them from nearby sites and establishing the final pattern of widely spaced crossovers. This model not only accounts for the regulation of crossover interference but also helps explain the obligatory crossover, and it has gained experimental support across multiple species [3,9,13]. Since DSBs are produced in excess of crossovers, the key to understanding crossover interference lies in how crossovers are selected from among all DSBs.

Class I crossovers are directly governed by a group of evolutionarily conserved pro-crossover proteins, which stabilize recombination intermediates and promote their resolution into crossovers [4,14–16]. Pro-crossover proteins are thought to function as a complex, though a complete composition of such a complex is still lacking [14]. This group of factors, comprising the Zip3 family of proteins [17,18], the conserved MutSγ complex [19,20], COSA-1/CNTD1 [21–23], and the MutLγ complex [24], dynamically associate with the chromosomes in a meiosis stage-dependent manner, under the regulation of protein-protein interaction(s) [25]. However, the mechanisms underlying the dynamic localization of meiotic proteins remain poorly understood.

Following DSBs formation, pro-crossover proteins, such as HEI10, RNF212 in mouse and MSH-5 in *C. elegans*, initially accumulate at multiple early recombination intermediates [25–29]. As meiotic prophase progresses, the distribution of most pro-crossover proteins exhibits ‘interference’ along the chromosomes [13,21,25,26]. Their focus number gradually decreases and the remaining foci specifically mark crossover sites, a process known as crossover designation. Intriguingly, *C. elegans* COSA-1 foci display exceptionally robust interference patterning, making it a commonly used cytological marker for crossover designation [21]. These findings collectively suggest that pro-crossover proteins play critical roles in the differentiation between crossover and non-crossover and in the implementation of crossover interference, potentially serving as direct targets of crossover control mechanisms. Therefore, a comprehensive study of the spatiotemporal regulation and functional modulation of pro-crossover proteins would be crucial for elucidating the fundamental mechanisms governing crossover patterning.

Protein ubiquitination plays important roles in regulating protein function and cellular activities [30]. The type of ubiquitination, such as mono- versus poly-ubiquitination, and the way in which poly-ubiquitination is formed can lead to different cellular outcomes [31]. Two independent cross-species studies demonstrated that the ubiquitin-proteasome system (UPS) localizes to meiotic chromosome axes during prophase I, suggesting a conserved role in regulating meiotic progression [32,33]. Specifically, cytological analyses in one study employing immunostaining of meiotic recombination proteins in mouse spermatocytes revealed that UPS inhibition disrupts the normal dynamics of pro-crossover factors [33]. However, it remains unknown whether these dynamic alterations occur at the transcriptional or post-translational level.

Since then, the UPS has emerged as a master coordinator of meiotic prophase I, governing events ranging from meiosis entry [34], DSB formation [35], SC dynamics [36–38] to meiotic recombination [39], chromosome axis length fine-tuning [40], and meiotic surveillance [41]. Notably, the proteasome has also been shown to be directly involved in crossover/non-crossover differentiation in yeast by degrading unphosphorylated Msh4 from the non-crossover sites [42]. However, this role of the proteasome is ubiquitin-independent. Thus, the contribution of ubiquitination to pro-crossover factor turnover and crossover patterning remains to be determined.

*C. elegans* is an ideal model system to study the regulatory mechanisms of crossover formation, particularly the enforcement of crossover interference. During meiosis in *C. elegans*, homologous chromosome pairs are strictly limited to a single crossover event [6,21,43,44]. Any increase in crossover frequency implies a failure in crossover interference implementation. Here, we dissect the function of the UPS in crossover control, specifically its regulation of pro-crossover proteins and crossover formation. Through targeted disruption of the UPS, we demonstrate both shared and distinct roles for ubiquitination and proteasomal degradation in the regulation of pro-crossover protein dynamics. While both ubiquitination and proteasomal degradation control the abundance and chromosomal distribution of pro-crossover factors along pachytene chromosomes, ubiquitination exclusively governs the dissociation of pro-crossover factors from crossover sites after crossover formation in diakinesis nuclei. Collectively, our findings establish the UPS as a critical post-translational regulator that orchestrates spatiotemporal dynamics of pro-crossover proteins to ensure accurate crossover patterning, revealing a previously unrecognized restrictive role of the UPS in crossover formation.

## Results

### Disruption of the UPS leads to elevated pro-crossover protein levels in *C. elegans*

**Fig 1:**
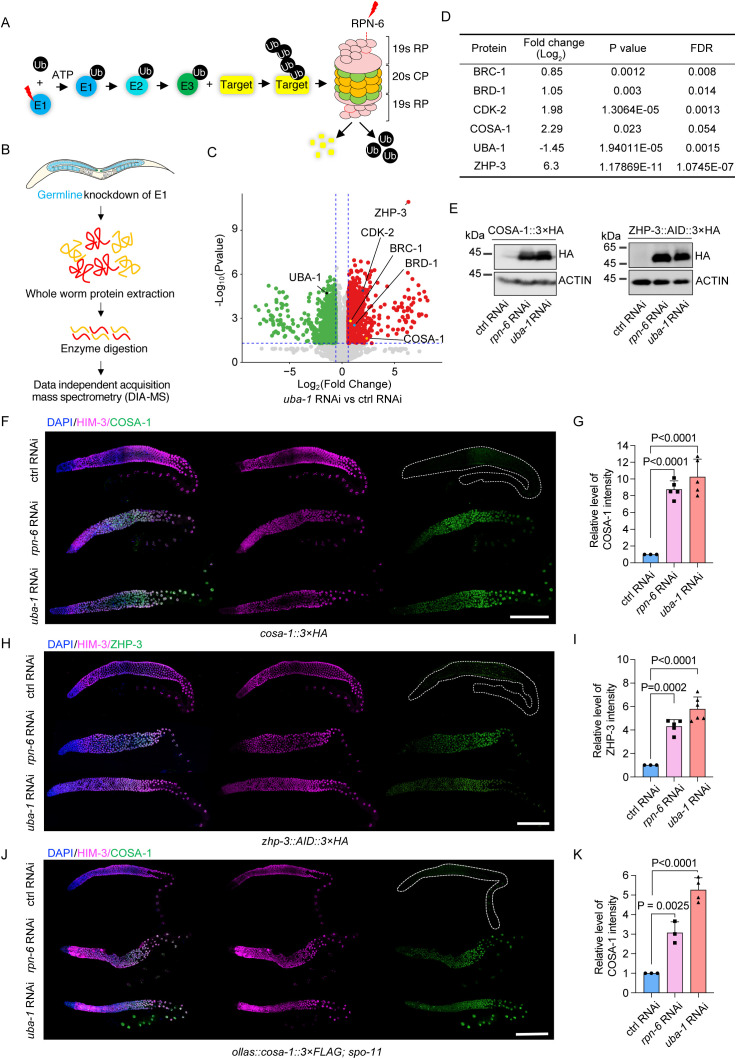
Knockdown of the UPS led to increased abundance of pro-crossover proteins. **(A)** Schematic of the ubiquitin-proteasome system. E1 and RPN-6 were targeted by RNAi in this study. E1, ubiquitin-activating enzyme; E2, ubiquitin-conjugating enzyme; E3, ubiquitin ligase; CP, core particle; RP, regulatory particle. **(B)** Schematic of the germline of *Caenorhabditis elegans* and the workflow of DIA proteomics. **(C)** Volcano plot showing changes in protein level after germline-specific *uba-1* RNAi compared with ctrl RNAi. Dots representing UBA-1, BRC-1, BRD-1, CDK-2, COSA-1 and ZHP-3 are highlighted. The volcano plot was executed with R package ggplot2. **(D)** Table listing the fold change, *P* value, and false discovery rate (FDR) for proteins highlighted in (C). **(E)** Western blot showing changes in protein level of COSA-1::3 × HA and ZHP-3::AID::3 × HA upon ctrl, *rpn-6*, and *uba-1* RNAi treatments. **(F)** Immunofluorescence images of dissected gonad from *cosa-1::3 × HA* treated with ctrl, *rpn-6*, or *uba-1* RNAi, stained for DNA (DAPI, blue), HIM-3 (magenta), and COSA-1 (green). **(G)** Quantification of immunofluorescence intensity of COSA-1 in (F). **(H)** Immunofluorescence images of dissected gonad from *zhp-3::AID::3 × HA*, treated with ctrl, *rpn-6*, or *uba-1* RNAi, stained for DAPI (blue), HIM-3 (magenta), and ZHP-3 (green). **(I)** Quantification of immunofluorescence intensity of ZHP-3 in (H). **(J)** Immunofluorescence images of dissected gonad from *ollas::cosa-1::3 × FLAG; spo-11*, treated with ctrl, *rpn-6*, or *uba-1* RNAi, stained for DAPI (blue), HIM-3 (magenta), and COSA-1 (green). **(K)** Quantification of immunofluorescence intensity of COSA-1 in (J). Signals from the distal tip of the gonad to the end of pachytene were quantified. *n* ≥ 3. The means ± SD are shown. *P* values are given by ordinary one-way ANOVA. All scale bars, 100 μm. The underlying data for Fig 1C can be found in S1 Table, and for Fig 1G, 1I, and 1K in S1 Data.

To systematically investigate the potential targets of the UPS in the germline of *C. elegans*, we adopted a data-independent acquisition (DIA) proteomics strategy. Germline-specific RNAi of *uba-1*, the sole E1-encoding gene in *C. elegans*, was employed to inhibit polyubiquitination initiation (Fig 1A and 1B) [45,46]. The resulting up-regulated proteins were then identified and analyzed compared with control RNAi. The proteomics data revealed a ~60% reduction in UBA-1 abundance, demonstrating the efficacy of our germline-specific RNAi (S1A Fig). Meanwhile, many known UPS targets, including the cyclins and WEE-1, were up-regulated (S1 Table), showing that the function of the UPS was indeed impaired. Biological Processes enrichment reveals that proteins functioning in meiotic cell cycle are enriched (S1B Fig). Among the enriched meiotic proteins are the evolutionarily conserved pro-crossover factors, such as COSA-1, ZHP-3, and CDK-2 (Figs 1C, 1D, and S1A; S1 Table), as well as BRC-1 and BRD-1 (Fig 1C and 1D), which were found to concentrate at crossover-designated sites, colocalizing with pro-crossover factors [47].

To validate the up-regulation of the pro-crossover factors, we performed immunoblotting on whole worm lysates treated with either control RNAi, *uba-1* RNAi, or *rpn-6.1* RNAi (hereafter referred to as *rpn-6* RNAi). The latter is a subunit of 19S proteasome and has been used in a previous study to decrease proteasome function (Fig 1A) [48]. We found that pro-crossover proteins, such as COSA-1, MSH-5, and ZHP-3, were difficult to be detected in whole worm lysates, likely due to low abundance (Figs 1E, S1C, and S1D). Strikingly, following *uba-1* and *rpn-6* RNAi treatments, these proteins became readily detectable, such as MSH-5 (knock-in GFP::MSH-5 in S1C Fig), COSA-1 (detected by both knock-in HA-tagged in Fig 1E and transgenic GFP::COSA-1 in S1D Fig), ZHP-3 (knock-in HA-tagged in Fig 1E), and HIM-6 (knock-in eGFP::HIM-6 in S1C Fig), indicating a significant increase in their levels. Notably, the level of a GFP-tagged SC component SYP-3 remained unchanged (S1E Fig), suggesting that pro-crossover factors might be preferentially targeted over other meiotic proteins. Immunofluorescence analysis was then performed on dissected germline to further confirm the increase in protein abundance. Consistent with the immunoblotting results, a significant increase in COSA-1 and ZHP-3 was observed upon depletion of *uba-1* and *rpn-6* by RNAi (Fig 1F–1I). Notably, both transgenic overexpression lines (using promoters different from the endogenous ones) and endogenous knock-in alleles of *cosa-1* exhibited identical trends in protein accumulation, strongly suggesting that the observed effects are mediated through post-translational regulation. This conclusion is further supported by RNA-seq data, which showed no significant alterations in the mRNA levels of known pro-crossover factors (S1F Fig and S2 Table).

We next set out to investigate the genetic requirement(s) for the up-regulation of pro-crossover factors. Proteolysis of pro-crossover factors has been proposed to facilitate crossover/non-crossover determination [33,42]. For instance, yeast Msh4 phosphorylation at crossover sites prevents its proteasomal degradation [42]. If the mechanism is conserved in *C. elegans*, the increase in pro-crossover protein levels following disruption of the UPS could be due to the non-degraded pro-crossover proteins from the non-crossover sites. To test this hypothesis, we reasoned that a DSB-deficient background, in which crossover designation cannot occur, would eliminate the need for the proposed proteolytic regulation of pro-crossover factors. We generated a *spo-11* null mutant using CRISPR-Cas9 to block endogenously programmed DSB induction [49], as evidenced by the presence of 12 univalents in the diakinesis oocytes (S1G Fig). In *uba-1* or *rpn-6* RNAi treated *spo-11* mutants, the increase in COSA-1 protein levels still occurred (Fig 1J and 1K), suggesting that UPS-mediated regulation of pro-crossover factors operates independently of SPO-11-dependent DSB formation.

### Crossover designation is maintained despite depletion of UBA-1 or RPN-6

**Fig 2:**
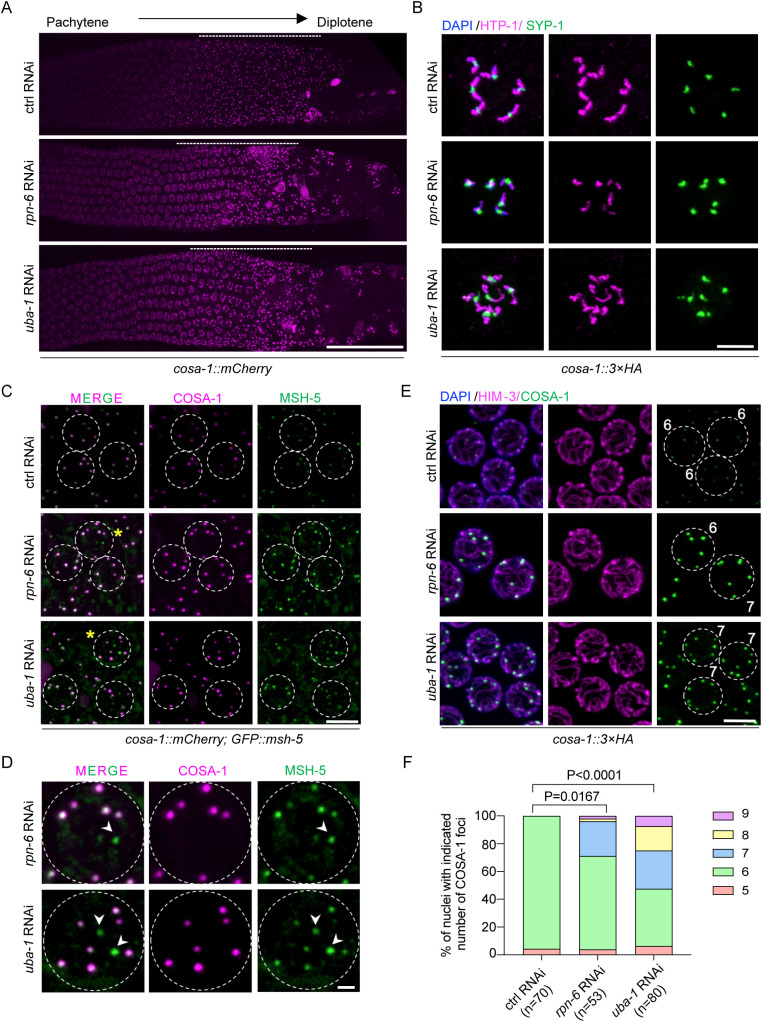
Knockdown of the UPS led to increased intensity and focus the number of pro-crossover protein. **(A)** Fluorescence images of a portion of gonad from *cosa-1::mCherry* worms treated with ctrl, *rpn-6*, or *uba-1* RNAi, extending from early pachytene through early diplotene. Note the overall increase in nucleoplasmic COSA-1::mCherry and COSA-1::mCherry focus intensity following *rpn-6* and *uba-1* knockdown. **(B)** Immunofluorescence images of diplotene nuclei from *cosa-1::3 × HA* worms treated with ctrl, *rpn-6*, or *uba-1* RNAi, stained for DNA (blue), HTP-1 (magenta), and SYP-1 (green). **(C)** Fluorescence images of late pachytene nuclei from *cosa-1::mCherry; GFP::msh-5* worms treated with ctrl, *rpn-6*, or *uba-1* RNAi, showing COSA-1::mCherry (magenta) and GFP::MSH-5 (green). Individual nuclei were outlined by dotted circle. **(D)** Enlargements of two nuclei marked by the yellow star in (C). Arrowheads point to GFP::MSH-5 foci not overlapping with COSA-1::mCherry. **(E)** Immunofluorescence images of late pachytene nuclei from *cosa-1::3 × HA* worms treated with ctrl, *rpn-6*, or *uba-1* RNAi, stained for DAPI (blue), HIM-3 (magenta), and COSA-1 (green). **(F)** Quantification of the percentage of late pachytene nuclei with the indicated number of COSA-1::3 × HA foci as shown in **(E)**. *P* values are given by ordinary one-way ANOVA. Scale bars, 50 μm for (A), 5 μm for (B), (C), and (E), 1 μm for (D). The underlying data for Fig 2F can be found in S1 Data.

Our data demonstrate that the UPS regulates protein level of the pro-crossover factors. To further characterize the impacts of UPS knockdown beyond protein abundance, we carefully examined pro-crossover protein signals from meiotic entry to late pachytene, when CO designation happens (S2A Fig). Consistent with previous studies, knockdown of the UPS through *rpn-6* or *uba-1* RNAi led to the formation of polycomplexes containing the chromosomal axis component HIM-3 [32], along with shortened or even absent TZ region, which normally contains crescent-shaped nuclei (white arrows and yellow arrowheads in S2A Fig) [41]. In these knockdown conditions, elevated levels of COSA-1 and ZHP-3 were already detectable during early meiosis, primarily as diffuse signals in the nucleoplasm without forming distinct foci (S2A Fig, TZ and EP). Nevertheless, COSA-1 and ZHP-3 foci coalesced normally during mid-to-late pachytene, comparable to control (S2A Fig). Using an endogenous mCherry-tagged allele of *cosa-1*, *cosa-1::mCherry* [50], we observed a similar trend regarding COSA-1 dynamics, where the signal of COSA-1 is initially nucleoplasmic and later transitions to foci along the chromosomes. Notably, even though COSA-1 foci exhibited increased brightness and size upon *rpn-6* or *uba-1* RNAi, most likely due to elevated protein levels (Fig 2A), this enhancement was not associated with premature appearance of COSA-1 foci (Fig 2A, dotted white line denotes the LP region with COSA-1::mCherry foci), indicating that crossover designation timing is tightly controlled despite UPS impairment.

We also carefully examined COSA-1 foci formation in the *spo-11* mutant background. In agreement with previous studies, 1–2 COSA-1 foci were observed within the oocyte nuclei at late pachytene in *spo-11* mutants (S2B and S2C Fig). Following *rpn-6* RNAi, the number of foci remained largely unchanged, but their intensity was significantly increased, presumably due to elevated COSA-1 protein abundance (S2B and S2C Fig). Interestingly, *uba-1* RNAi led to a marked increase in the number of COSA-1 foci, indicating a fundamental divergence in how ubiquitination and the proteasome pathway regulate COSA-1 localization in this background.

Crossover designation induces the asymmetric distribution of certain SC-related proteins, such as the axial element HTP-1 and the central element SYP-1, leading to chromosome remodeling [51]. We found that the knockdown of the UPS did not result in any significant changes in the protein levels of HTP-1 and SYP-1 (S3A–S3C Fig), but diplotene chromosome morphology appears altered, such as shortened or disorganized axes, as indicated by HTP-1 staining (Fig 2B). We speculate that this is related to the previously reported role of the UPS in regulating the dynamics of these proteins [32]. Nevertheless, remodeling still occurred following RPN-6 or UBA-1 depletion (Fig 2B), albeit with some deviations from wild type, further demonstrating that crossover designation remains intact.

COSA-1 and MSH-5 co-accumulate at crossover designation sites during late pachytene, exhibiting interdependent chromosomal localization [21]. To simultaneously monitor their dynamics, we generated a *cosa-1::mCherry; GFP::msh-5* double-fluorescent strain [47]. Following UPS knockdown, while most COSA-1 foci colocalized with MSH-5, persistent MSH-5 foci lacking COSA-1 were consistently observed in late pachytene (Fig 2C and 2D, and arrowheads in Fig 2D). By quantification, we found the existence of both MSH-5-only and COSA-1-only foci, with the former being more frequent (S3D and S3E Fig). These persistent single-stained foci may represent either unprocessed recombination intermediates or non-degradable protein aggregates. These results suggest that the UPS may be involved in clearing pro-crossover proteins that have accumulated at non-crossover sites under physiological conditions. Given that MSH-5 accumulates at recombination intermediates earlier and forms more foci than COSA-1 [25,29], it presents more potential targets for UPS-mediated clearance, which could explain the higher frequency of MSH-5-only foci. Since the majority of COSA-1 foci co-localized with MSH-5 (S3D and S3E Fig), we next examined whether increased pro-crossover abundance is accompanied by an increase in crossover designation by quantifying focus number of COSA-1 per nucleus. Nuclear spreading immunofluorescence demonstrated significantly elevated number of COSA-1 focus (>6/nucleus; Fig 2E and 2F) after UPS depletion, indicating defective crossover interference.

### E1-mediated ubiquitination is required for the dissociation of pro-crossover proteins from diakinesis chromosomes

**Fig 3:**
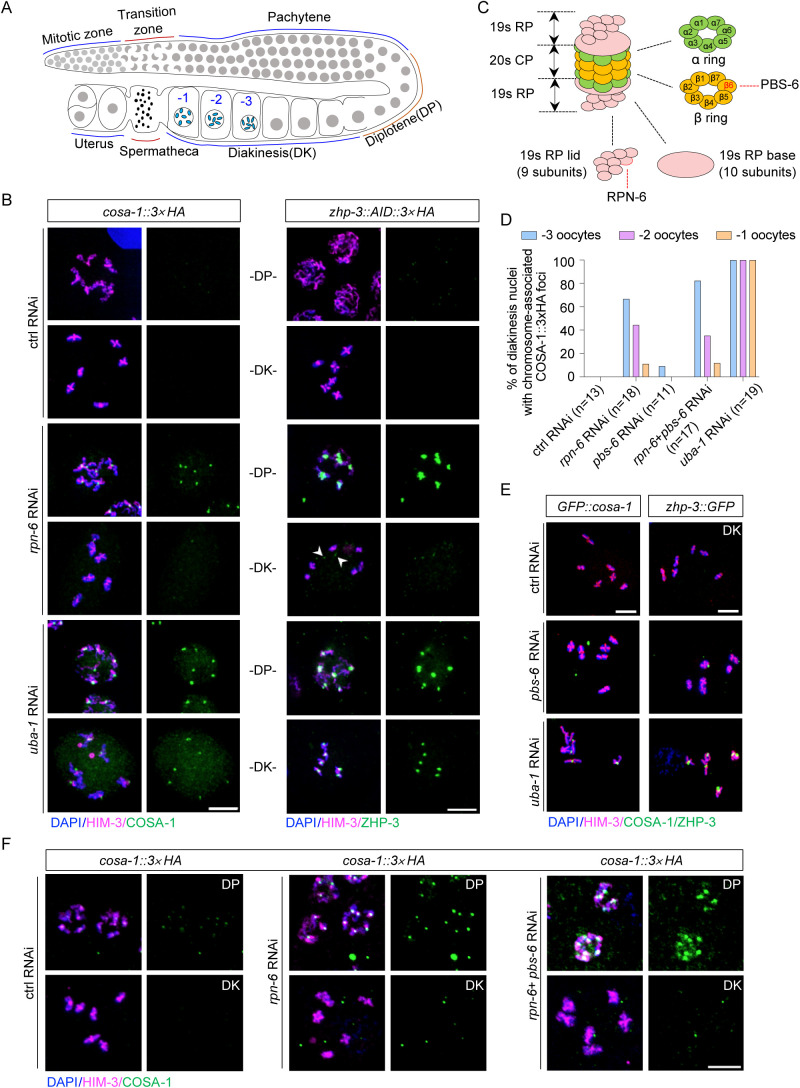
Knockdown of *E1* but not the proteasome led to persistent association of pro-crossover proteins with diakinesis chromosomes. **(A)** Schematic drawing of meiotic events during oogenesis in the *Caenorhabditis elegans* germline. **(B)** Immunofluorescence images of diplotene (DP) and diakinesis (DK) nuclei from *cosa-1::3 × HA or zhp-3::AID::3 × HA* worms treated with ctrl, *rpn-6*, or *uba-1* RNAi, stained for DAPI (blue), HIM-3 (magenta), and COSA-1 or ZHP-3 (green). **(C)** Schematic of the composition of the eukaryotic 26S proteasome. PBS-6 and RPN-6 are indicated. **(D)** Quantification of the percentage of diakinesis nuclei with chromosome-associated COSA-1::3 × HA foci for ctrl RNAi, *rpn-6* RNAi, *pbs-6* RNAi, *rpn-6* + *pbs-6* double RNAi, and *uba-1* RNAi. **(E)** Immunofluorescence images of diakinesis (DK) nuclei from transgenic *GFP::cosa-1* and *zhp-3::GFP* worms treated with ctrl, *pbs-6*, or *uba-1* RNAi, stained for DAPI (blue), HIM-3 (magenta), and COSA-1 (green) or ZHP-3 (green). **(F)** Immunofluorescence images of DP and DK nuclei from *cosa-1::3 × HA* worms treated with ctrl RNAi, *rpn-6* RNAi, and *rpn-6 + pbs-6* double RNAi, stained for DAPI (blue), HIM-3 (magenta), and COSA-1 (green). All scale bars, 5 μm. The underlying data for Fig 3D can be found in S1 Data.

Crossover designation does not necessarily mean successful crossover formation. Cross-species studies have shown that ubiquitination-dependent proteasomal degradation mediated by specific E3s actively promotes crossover formation [27,28,34,36–39,52]. Therefore, impairment of the UPS function would be supposed to disrupt crossover formation. In *C. elegans*, defects in crossover formation could be easily detected by examining the karyotypes of individual diakinesis-stage oocytes. In wild type, six DAPI-stained bodies are visible, representing six pairs of homologs linked by chiasmata, the cytological manifestations of crossover (Fig 3A). The appearance of individual chromosomes (univalents), or more than six DAPI-stained bodies, usually indicates a defect in crossover formation. To directly check the impact of UPS impairment on crossover formation, we examined bivalent formation in diakinesis. Univalent was never observed under *rpn-6* or *uba-1* knockdown conditions, implying efficient crossover formation (Fig 3B). Intriguingly, we observed that the pro-crossover factors COSA-1 and ZHP-3 remained associated with the chromosomes in diakinesis oocytes (Fig 3B), a pattern reminiscent of mutants defective in resolution of meiotic recombination intermediates [14].

During normal meiotic progression to diakinesis, pro-crossover proteins gradually dissociate from crossover sites, becoming undetectable in the majority of −3 and −2 and all −1 diakinesis oocytes [14] (Fig 3A and 3B). However, persistent chromosome-associated COSA-1 and ZHP-3 foci could be detected in −1 diakinesis oocytes following *uba-1* knockdown (Fig 3B). In *rpn-6* RNAi-treated oocytes, although large and bright chromosome-associated COSA-1 and ZHP-3 foci were readily detected in diplotene, a large proportion of these foci vanished as the oocyte progressed into −1 diakinesis (compare *rpn-6* RNAi with *uba-1* RNAi in Fig 3B and 3D). The remnant foci of COSA-1 and ZHP-3 also exhibited distinct localization pattern, where most foci were in the nucleoplasm of the diakinesis oocytes (Fig 3B, arrowheads), reflecting that COSA-1 and ZHP-3 complexes could be dissociated from chromosomes but were unable to be degraded. The persistent chromatin association of ZHP-3 and COSA-1 in *uba-1* RNAi-treated oocyte was not due to a difference in protein levels, since both COSA-1 and ZHP-3 were similarly increased upon *rpn-6* or *uba-1* knockdown. Considering that RPN-6 belongs to the 19S regulatory particle lid complex, we then tested *pbs-6* RNAi, which targets the 20S proteasome’s catalytic core (Fig 3C). Similar to *rpn-6*, *pbs-6* knockdown led to upregulation of COSA-1, as revealed by western blot and IF (S4A–S4C Fig), but no chromosome-associated COSA-1 could be detected in −1 diakinesis oocytes either (Fig 3D).

We next used GFP-tagged transgenic *cosa-1* and *zhp-3* worms to further validate these findings. A similar pattern was observed, whereby the dissociation of COSA-1 and ZHP-3 from chromosomes was exclusively delayed upon *uba-1* knockdown but not *pbs-6* knockdown (Figs 3E and S4D). Considering the distinct effects of disrupting ubiquitination (*uba-1*) and proteasome (*rpn-6*, *pbs-6*) on the dissociation of the pro-crossover proteins, we performed a double knockdown by combining *rpn-6* and *pbs-6* in a single RNAi vector to test whether simultaneously disrupting both the regulatory particle and the catalytic core of the proteasome could block chromosomal dissociation of these factors. However, knocking down both *rpn-6* and *pbs-6* did not enhance the pro-crossover protein retention phenotype as compared with single RNAi; instead, most of the observed foci were either nucleoplasmic or chromosome-adjacent (Fig 3D and 3F).

Together, the above data indicate that ubiquitination, but probably not proteasomal degradation, is responsible for the dissociation of pro-crossover proteins from diakinesis chromosomes. This mechanism is reminiscent of how polyubiquitination directs the disassembly of stable complexes, such as the replisome (see the Discussion for further details).

### The CDC-48^UFD-1/NPL-4^ segregase is involved in the dissociation of pro-crossover proteins from meiotic chromosomes

**Fig 4:**
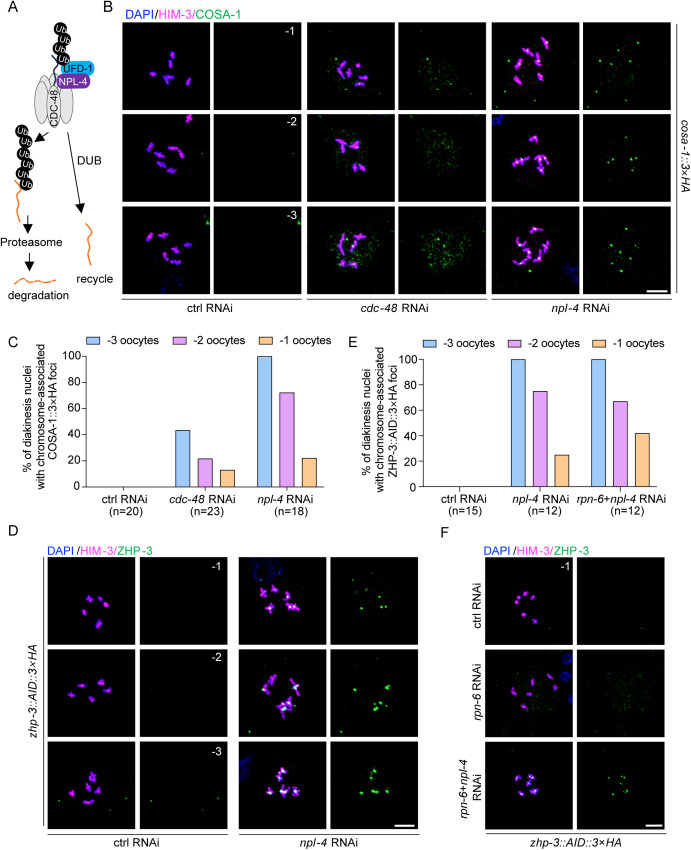
The CDC-48^UFD-1/NPL-4^ segregase is required for stage-specific dissociation of pro-crossover proteins from chromosomes. **(A)** Schematic showing the fate of a polyubiquitinated substrate protein with the CDC-48^UFD-1/NPL-4^ segregase. DUB, deubiquitinase. **(B)** Immunofluorescence images of diakinesis (−1, −2, and −3) nuclei from *cosa-1::3 × HA* worms treated with ctrl, *cdc-48*, or *npl-4* RNAi, stained for DAPI (blue), HIM-3 (magenta), and COSA-1 (green). **(C)** Quantification of the percentage of diakinesis nuclei with COSA-1::3 × HA foci for **(B)**. **(D)** Immunofluorescence images of diakinesis nuclei from *zhp-3::AID::3 × HA* worms treated with ctrl RNAi or *npl-4* RNAi, stained for DAPI (blue), HIM-3 (magenta), and ZHP-3 (green). **(E)** Quantification of the percentage of diakinesis nuclei with ZHP-3::AID::3 × HA foci for (D) and (F). **(F)** Immunofluorescence images of −1 diakinesis nuclei from *zhp-3::AID::3 × HA* worms treated with ctrl RNAi, *rpn-6* RNAi, or *rpn-6 + npl-4* double RNAi, stained for DAPI (blue), HIM-3 (magenta), and ZHP-3 (green). All scale bars, 5 μm. The underlying data for Fig 4C and 4E can be found in S1 Data.

The CDC-48^UFD−1/NPL−4^ segregase is best known for its function in the recognition and triaging of poly-ubiquitinated substrate(s) for proteasomal degradation or recycling [53,54] (schematic in Fig 4A). We therefore tested the hypothesis that a similar ubiquitin-segregase relay is involved in the process of crossover formation. To test this hypothesis, we first performed RNAi experiments targeting *cdc-48* and *npl-4*. Similar to *uba-1* RNAi treatment, *cdc-48* or *npl-4* depletion led to significantly persistent association of COSA-1 and ZHP-3 foci with diakinesis chromosomes, as foci could be detected even in −1 diakinesis oocytes (Figs 4B–4E and S5A–S5C). This is consistent with the idea that CDC-48 and NPL-4 function within the same pathway. Since *npl-4* RNAi produced a stronger effect than *cdc-48* RNAi (Figs 4C and S5A–S5C), we subsequently used *npl-4* alone to represent the function of the underlying CDC-48^UFD-1/NPL-4^ segregase complex. We also noticed an evident phenotypic difference between *npl-4* RNAi and *uba-1* RNAi when COSA-1::HA was monitored (20% versus 100%, Figs 3D and 4C). We reason that this likely reflects limitations in detection sensitivity, because when *npl-4* RNAi was performed in transgenic *GFP::cosa-1* and *zhp-3::GFP* worms, the penetrance of the delayed dissociation phenotype (70%–100%) (S5B and S5C Fig) approached that observed in *uba-1* RNAi (100%) (S4D and S5B Fig).

Unlike *uba-1* RNAi, *npl-4* depletion did not result in an increase in COSA-1 or ZHP-3 (S5D–S5G Fig), even though both *cdc-48*^*ufd-1/npl-4*^ and *uba-1* knockdown resulted in delayed dissociation of pro-crossover factors at diakinesis. Therefore, in contrast to ubiquitination, CDC-48^UFD-1/NPL-4^ specifically regulates the dissociation of pro-crossover factors from diakinesis chromosomes.

Based on these results, we propose a linear dissociation cascade with sequential steps: pro-crossover proteins are first polyubiquitinated, which leads to their recognition and displacement from chromosomes by the CDC-48^UFD-1/NPL-4^ complex; this displacement is then followed by the final degradation by the proteasome. Next, in order to further test if this displacement step is dependent on CDC-48^UFD-1/NPL-4^, we took advantages of the observation that knockdown of *rpn-6* alone didn’t impair the dissociation of pro-crossover proteins. We would expect the phenotype to revert from the *rpn-6* RNAi phenotype (non-chromosome-associated foci) to resemble the *uba-1* or *npl-4* RNAi phenotype (chromosome-associated foci). We performed double knockdown of *rpn-6* and *npl-4* and observed persistent chromosome-associated ZHP-3 foci in −1 diakinesis oocytes (Fig 4E and 4F), indicating *npl-4* is epistatic to *rpn-6* in the dissociation of pro-crossover factors. Collectively, our data establish that the CDC-48^UFD-1/NPL-4^ segregase is required for efficient dissociation of pro-crossover complex from diakinesis chromosomes. However, we cannot formally exclude the possibility that another segregase is also involved.

### UPS knockdown rescues the crossover defect in a hypomorphic allele of *cosa-1*

**Fig 5:**
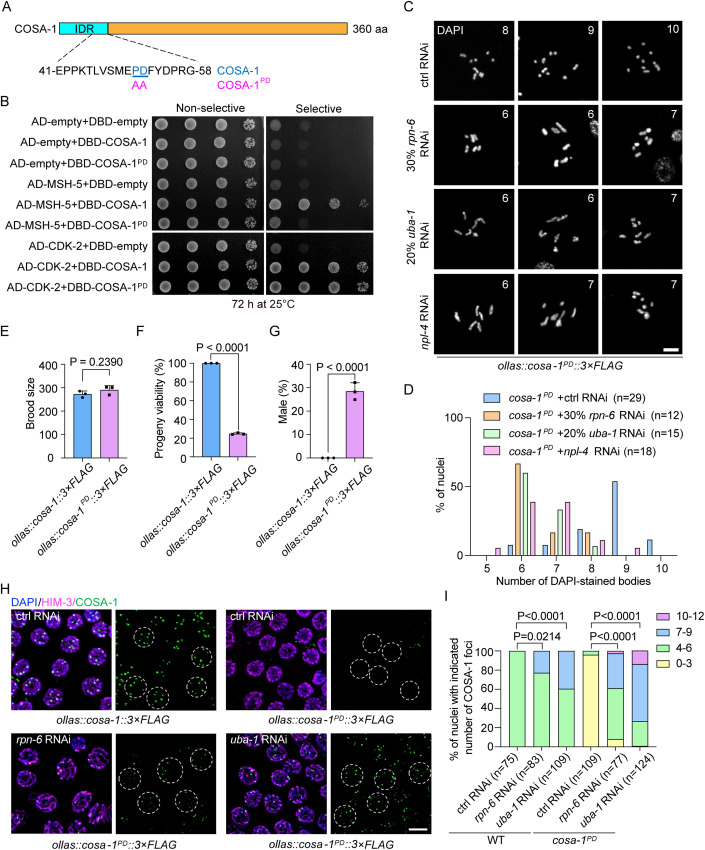
Knockdown of the UPS and NPL-4 rescued chromosomal accumulation of COSA-1 and bivalent formation in *cosa-1*^*PD*^. **(A)** Schematic of the primary structure of COSA-1. The cyan rectangle represents the N-terminus of COSA-1, which is predicted to contain an intrinsically disordered region (IDR) responsible for interactions with other pro-crossover factors. P51 and D52 are underlined and highlighted in green. **(B)** Yeast two-hybrid assay showing that COSA-1^PD^ can bind efficiently to CDK-2, but hardly binds to MSH-5. **(C)** Fluorescence images of diakinesis nuclei stained for DAPI, showing bivalent formation in *ollas::cosa-1*^*PD*^*::3 × FLAG* worms treated with ctrl RNAi, 30% *rpn-6* RNAi, 20% *uba-1* RNAi, or *npl-4* RNAi. **(D)** Quantification of the percentage of nuclei with the number of DAPI-stained bodies indicated in (C). **(E–G)** Quantification of brood size (E), progeny viability (F), and male progeny frequency (G) for *ollas::cosa-1::3 × FLAG* and *ollas::cosa-1*^*PD*^*::3 × FLAG*, respectively. The means ± SD are shown. *P* values are given by *t* test. **(H)** Immunofluorescence images of late pachytene nuclei of the indicated genotypes stained for DAPI (blue), HIM-3 (magenta), and COSA-1 (green). **(I)** Quantification of the percentage of late pachytene nuclei with indicated number of COSA-1 or COSA-1^PD^ foci as shown in **(H)**. Quantification for *rpn-6* and *uba-1* single RNAi treatment in the wild-type background was also included. *P* values are given by ordinary one-way ANOVA. All scale bars, 5 μm. The underlying data for Fig 5D–5G and 5I can be found in S1 Data.

The persistent association of pro-crossover factors with meiotic chromosomes observed above could be explained by an alternative possibility that late recombination intermediates were not processed into mature crossovers after *uba-1* or *npl-4* knockdown, as observed for *slx-4* mutants [14]. To further establish the role of the UPS in pro-crossover factor regulation and crossover formation, we introduced a crossover-deficient condition specifically induced by mutation in the pro-crossover factor COSA-1. P51 and D52 are two amino acids in COSA-1 that are mutated in the *cosa-1-6A* allele but not *in cosa-1-4A*, two alleles generated in a recent study from our lab [14]. Because *cosa-1-6A* exhibits more severe meiotic defects than *cosa-1-4A*, a double-alanine substitution mutant (P51A, D52A) was generated to assess the specific functional contribution of these residues, and this allele was named *cosa-1*^*PD*^ (Fig 5A). Like COSA-1-4A and COSA-1-6A [14], COSA-1^PD^ exhibited reduced binding affinity for other pro-crossover proteins such as MSH-5, while its interaction with CDK-2 was maintained in yeast two-hybrid assays (Fig 5B). Homozygous *cosa-1*^*PD*^ worms showed partial crossover defects, as evidenced by an average of 8.54 DAPI-stained bodies in diakinesis oocytes (Fig 5C and 5D). In contrast to other *cosa-1* mutants [14], *cosa-1*^*PD*^ could be homozygous viable and fertile, with normal brood size, reduced progeny viability, and increased incidence of male offspring (Fig 5E–5G), making this allele ideal for probing UPS-dependent crossover regulation.

Depletion of UBA-1 was thus performed in *cosa-1*^*PD*^ mutants. *uba-1* partial RNAi was used to obtain well resolved and separated chromosomes for quantification. After 20% *uba-1* RNAi treatment (diluted 1:4 with control RNAi), we observed partial rescue of bivalent formation defect in *cosa-1*^*PD*^ mutants, with an average of 6.47 DAPI-stained bodies compared with 8.54 in untreated controls (Figs 5C, 5D, and S6A). This argues against the idea that the persistent pro-crossover protein foci marked unresolved recombination intermediates. Instead, it strongly suggests that crossovers were efficiently generated upon *uba-1* knockdown. Partial knockdown of *rpn-6* (30%) also reduced the number of DAPI-stained bodies in *cosa-1*^*PD*^, implying that the UPS negatively regulates crossover formation (Fig 5C and 5D).

In *cosa-1*^*PD*^ mutants, COSA-1 foci are barely detectable in late pachytene (Fig 5H), even though crossover designation still seems to happen as manifested by the formation of 6 MSH-5 foci at late pachytene (S6B and S6C Fig). We therefore investigated whether the *uba-1* and *rpn-6* RNAi-mediated rescue of bivalent formation involves restoration of COSA-1 localization and accumulation. Strikingly, knockdown of the UPS by either *rpn-6* or *uba-1* RNAi significantly recovered the number of COSA-1 foci (7.5 foci for *uba-1* RNAi and 6.05 foci for *rpn-6* RNAi) (Fig 5H and 5I). These findings strongly suggest that the UPS restricts crossover formation by controlling the stability and chromosomal retention of pro-crossover factors at the crossover designation sites.

We further used *cosa-1*^*PD*^ to test if the CDC-48^UFD-1/NPL-4^ segregase also regulates crossover formation. Intriguingly, *npl-4* RNAi was also able to partially rescue bivalent formation in *cosa-1*^*PD*^ mutants (Fig 5C and 5D), suggesting that it likewise participates in restricting crossover formation. However, unlike *uba-1* and *rpn-6*, knockdown of *npl-4* did not restore the accumulation of COSA-1 during late pachytene, indicating that the UPS may have a broader functional scope than the CDC-48^UFD-1/NPL-4^ segregase (S6D Fig).

### The UPS enforces crossover interference in *C. elegans*

**Fig 6:**
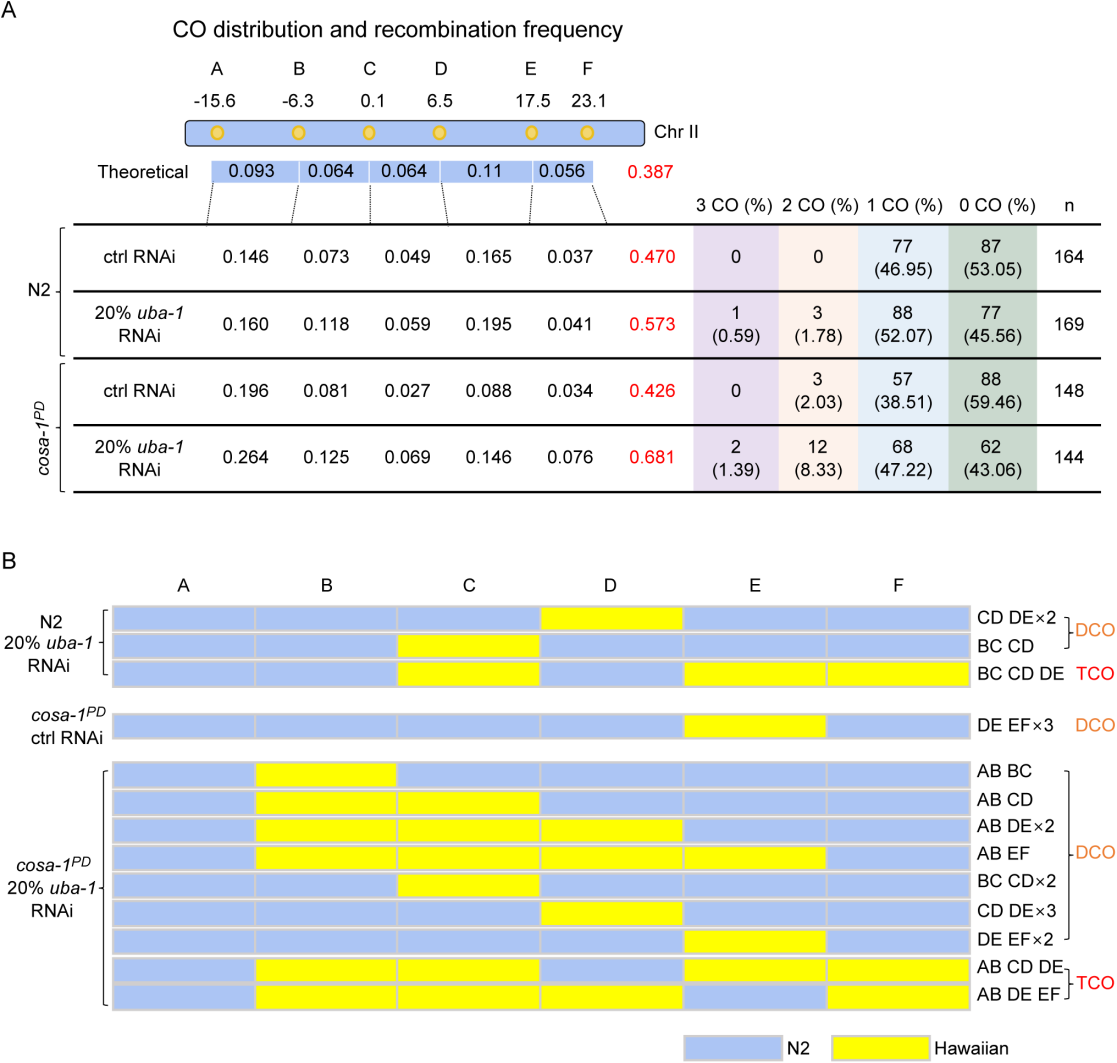
Knockdown of the UPS attenuated crossover interference. **(A)** Crossover frequency and distribution on chromosome II of the indicated genotypes with different RNAi treatments were evaluated. Red-highlighted values denote the sum of the 5 intervals. n is the number of total progenies scored. The frequency of triple crossovers, double crossovers, single or zero crossover per chromosome is also indicated. **(B)** Crossover distribution of double crossover and triple crossover events detected. Blue indicates the Bristol N2-derived allele, and yellow indicates the Hawaiian-derived allele. The number indicates how many times double crossovers occurring at the same location were detected.

Only one crossover per homolog pair is normally generated during meiosis in *C. elegans*, representing an extreme example of crossover interference [55]. The above data therefore indicates a role for the UPS in crossover interference. To directly test the involvement of the UPS in crossover interference, we measured the recombination rates on chromosome II using a recently established Insertion-Deletion-Polymorphisms method [56]. *uba-1* RNAi increased both overall crossover frequency and the incidence of double crossovers and triple crossovers. Although the difference in the frequency of multiple crossovers between control RNAi and *uba-1* partial RNAi does not reach statistical significance (Fisher’s exact test, one-tailed, *P* = 0.065), the presence of more than one crossover strongly supports the hypothesis that the ubiquitin system plays a critical role in restricting crossover designation and formation (Fig 6A).

The recombination rates were next assessed in the *cosa-1*^*PD*^ background. Consistent with the presence of univalent at diakinesis and the HIM (High incidence of Male progeny) phenotype, *cosa-1*^*PD*^ exhibited a reduced recombination rate (Fig 6A, 0.426 versus 0.470). However, double crossovers were detected in *cosa-1*^*PD*^, indicative of impaired crossover interference (Fig 6A). Partial *uba-1* RNAi rescued the reduction in recombination rate of *cosa-1*^*PD*^, consistent with the restrictive role of ubiquitination in crossover control (Fig 6A). Intriguingly, *uba-1* RNAi in *cosa-1*^*PD*^ caused more severe crossover interference defects compared with equivalent *uba-1* knockdown in the wild-type background, whereby the incidence of double and triple crossovers was further elevated (Fig 6A, compare the effect of 20% *uba-1* RNAi treatment in N2 with *cosa-1*^*PD*^). This is in agreement with our observations that a subset of nuclei in *cosa-1*^*PD*^ mutants contained more than 9 COSA-1 foci upon *uba-1* RNAi, a number never reached in wild type with the same treatment (Fig 5I). Consistent with the formation of multiple crossovers, ring-like bivalents could be detected in both the wild-type and *cosa-1*^*PD*^ diakinesis oocyte following *uba-1* RNAi (S7 Fig, yellow arrows). Notably, the frequency of multiple crossovers is significantly higher in the *uba-1* partial RNAi group than control in the *cosa-1*^*PD*^ background (Fisher’s exact test, one-tailed, *P* = 0.0063), reinforcing the idea that the ubiquitin system restricts crossover formation. Given that COSA-1^PD^ exhibits severely impaired binding to other pro-crossover factors, such as MSH-5, these results suggest that the interaction between COSA-1 and MSH-5 may contribute to the enhancement of crossover interference regulation. A preliminary analysis of the available double and triple crossover events revealed no consistent spacing preference, which may indicate a fundamental breakdown of crossover spacing constraints following UBA-1 depletion (Fig 6B). This disruption of spatial patterning provides further evidence that the UPS ordinarily enforces crossover interference.

### The UPS and the synaptonemal complexes (SC) differ in their mechanism underlying crossover interference

The synaptonemal complexes (SC) has long been implicated in crossover interference [57,58]. Since disruption of the UPS was previously shown to lead to defects in SC dynamics [32,33], the impacts of UPS knockdown on crossover designation and formation reported here might be simply attributed to the role of the UPS in synapsis. As the synaptonemal complex central region (SC-CR) proteins (e.g., SYP-1, -2, -3, -4, -5, or -6) are essential for crossover formation in *C. elegans*, it is not feasible to evaluate the function of the UPS in crossover patterning in mutants completely lacking any SYP protein. However, previous studies have shown that partial depletion of the SC central element, such as SYP-1, can attenuate crossover interference without compromising its crossover-promoting function of the SC [6,59–61]. Given that UPS knockdown in this study similarly impairs crossover interference, we compared partial depletion of SYP-1 with that of UBA-1 for their impacts on crossover formation in the *cosa-1*^*PD*^ background, attempting to uncover an SC-independent function of the UPS in crossover regulation.

We first evaluated *syp-1* partial RNAi efficacy and found that it recapitulated what had been used to demonstrate the role of the SC in limiting crossovers: the TZ zone was extended, as would be expected if SC-CR function is disrupted (S8A and S8B Fig); a significant increase in the number of COSA-1 foci at late pachytene (average 7.42, S8C and S8D Fig) and six bivalents in diakinesis (S8E Fig) were observed, indicating defective crossover interference albeit successful formation of chiasmata for all six chromosome pairs [6]. In contrast to knockdown of *uba-1* (Fig 5C, 5D, 5H, and 5I), partial depletion of SYP-1 could neither recover COSA-1 foci at late pachytene (S8F Fig), nor rescue bivalent formation of *cosa-1*^*PD*^ (S8G and S8H Fig). Instead, *syp-1* partial depletion exhibited strong genetic interaction with *cosa-1*^*PD*^ in bivalent formation: the crossover formation defect was exaggerated as suggested by the increased incidence of univalents (S8G and S8H Fig). Together, our genetic evidence strongly suggests that the UPS mediates crossover control through mechanisms separable from its regulation of SC dynamics. However, we cannot exclude that synapsis defects may still partially contribute to the crossover patterning changes observed upon UPS disruption.

### Meiotic proteins are direct targets of ubiquitination

**Fig 7:**
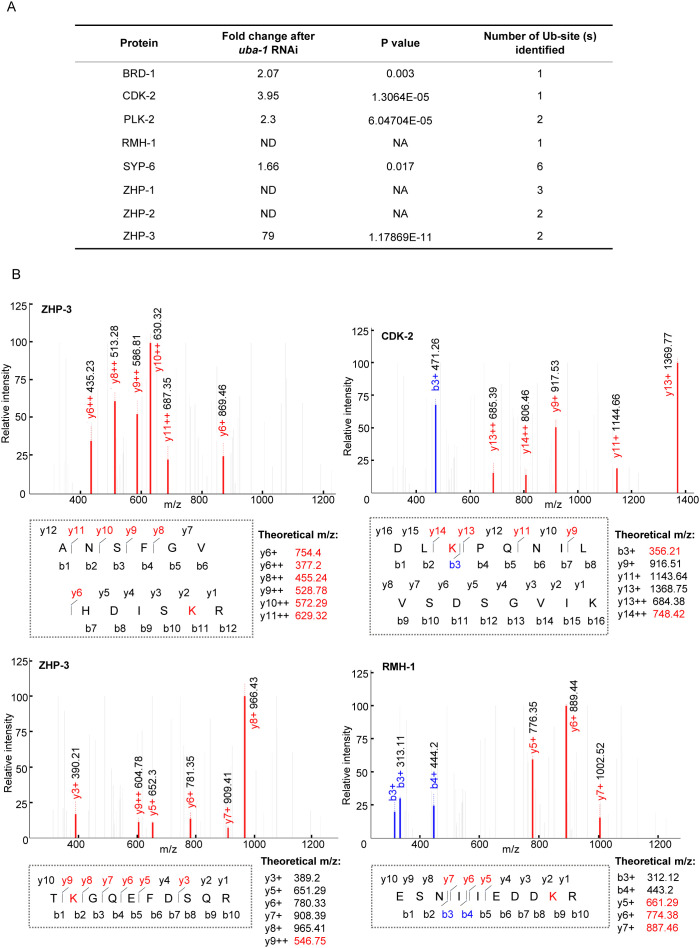
Pro-crossover proteins are subject to ubiquitination. **(A)** Meiotic proteins closely related to crossover designation were identified as being subject to ubiquitination. Data from the DIA proteomics study are included when available. Fold change and *P* value are for the DIA proteomics based on three biological replicates. NA, not applicable. ND means the protein was not detected by the DIA MS. Ubiquitination sites were verified based on the extracted ion chromatogram. **(B)** Secondary mass spectrum showing evidence of ubiquitination modification(s) for ZHP-3 (two sites), CDK-2, and RMH-1. The peptide was broken into charged fragments. The fragments to the left are named as b ions, and the fragments to the right as y ions. The detected fragments are marked by blue b or red y followed by the number of the fragment’s amino acid. *m*/*z* for the detected fragment is shown on the plot, and the theoretical *m*/*z* is shown on the side. The detected value is almost equal to the theoretical value when the lysine (K) in the fragment is not ubiquitinated. The red-highlighted theoretical values are 114 Da (the molecular weight of Gly-Gly) less than the detected ones, indicative of ubiquitination. *m*/*z*, mass to charge ratio; + , single charged; ++, double charged. The underlying data for this figure can be found in S1 and S3 Tables.

The data above demonstrate that the UPS regulates the dynamics of pro-crossover proteins during meiosis prophase. Although the pro-crossover factor ZHP-3 itself is a putative E3 ligase [18,62], ubiquitination of pro-crossover proteins remains poorly characterized, likely due to their low endogenous abundance. To determine whether pro-crossover factors undergo direct ubiquitination, nuclear extracts were prepared following germline-specific *rpn-6* RNAi, in order to enrich for potential polyubiquitinated proteins undergoing proteasomal degradation, and the ubiquitin-modified proteome was analysed by mass spectrometry. Consistent with previous findings that ubiquitin and proteasome localize along meiotic chromosome axes across species [32,33], SMC proteins, axis components, SC lateral components, and SC central components were found to undergo ubiquitination at multiple sites (Fig 7A and S3 Table). Intriguingly, pro-crossover proteins ZHP-3 and CDK-2 were found to possess reliable ubiquitin-modified residues (Fig 7A and 7B; S3 Table). Other meiotic proteins known to accumulate at the CO-designated sites during late pachytene, such as RMH-1 and BRD-1, and proteins localizing specifically on the short arm at late prophase, such as ZHP-1, ZHP-2, and PLK-2, are also subject to ubiquitination (Fig 7A and 7B; S3 Table). No ubiquitination site was identified for COSA-1, likely reflecting either the absence of ubiquitination for COSA-1 or its low abundance. Notably, the identified ubiquitinated proteins are not always accompanied with an increase in their abundance (Fig 7A), suggesting that these ubiquitination events may involve non-K48-linked poly-ubiquitin chains or mono-ubiquitination. While the functional consequences of these ubiquitination events require further investigation, these findings provide direct evidence that the crossover recombination repair machinery is broadly subject to ubiquitin-mediated regulation, and further support the hypothesis that the UPS directly regulates pro-crossover protein homeostasis through ubiquitination.

## Discussion

**Fig 8:**
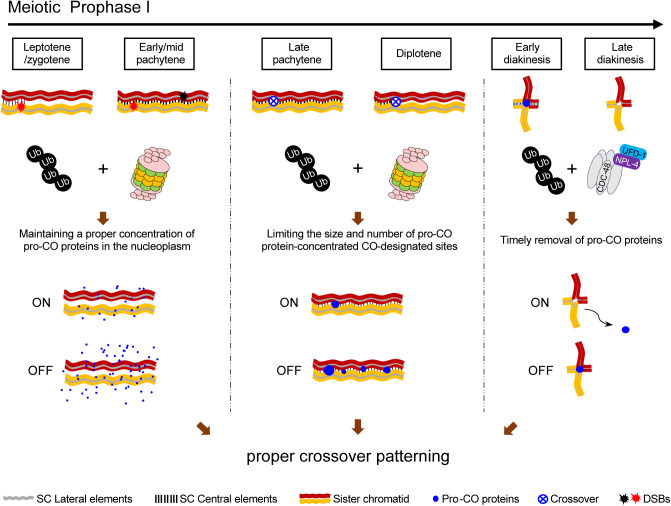
The UPS regulates the dynamics of pro-crossover proteins throughout meiotic prophase I. During early meiotic prophase I, the UPS helps to maintain the correct concentration of the pro-crossover protein pool. In late pachytene, the UPS restricts the accumulation of pro-crossover proteins to one focus per chromosome. After crossover formation, the pro-crossover protein complex dissociates from the crossover site in a ubiquitination-dependent manner, a process that involves the CDC-48^NPL-4/UFD-1^ segregase.

It has been previously shown in many organisms that the UPS has a prominent role in meiosis. Prevailing models posit that the UPS acts exclusively as a positive regulator of meiotic crossover formation, as evidenced by the catastrophic failure of crossover formation upon E3 ligase depletion or proteasome dysfunction across taxa [27,32,35–37,39,52,63,64]. In this study, by means of partial depletion, we establish the UPS as a key coordinator in regulating the dynamics of pro-crossover proteins and crossover patterning during *C. elegans* meiotic prophase I (Fig 8). Specifically, the UPS exerts dual regulatory control: (1) it governs proteostasis of the pro-crossover factors by limiting their total abundance through targeted degradation, and (2) it safeguards crossover interference, likely by preventing the aberrant accumulation or mislocalization of pro-crossover factors. Our work unveils a hidden restrictive role of the UPS in crossover formation: it actively prevents crossover overabundance. Alongside previous studies that have established positive roles for the UPS in crossover formation, our study demonstrates the UPS’s dual functionality in licensing crossover recombination while imposing constrains. This explains how meiotic cells avoid deleterious recombination while ensuring obligate exchanges.

Our cytological analysis, genetic rescue experiments, and recombination rate assessment, which revealed occurrence of multiple crossovers, all converge to indicate a regulatory role of UPS in restriction of crossover formation. Analysis of the positioning of the double and triple crossover events upon *uba-1* knockdown revealed no consistent spacing preference (Fig 6B). While the small sample size precludes a definitive statistical analysis of interference strength (e.g., calculation of the coefficient of coincidence), the presence of these multiple crossovers in a given chromosome and their seemingly random distribution is inconsistent with the robust crossover interference that normally ensures a single crossover per chromosome in *C. elegans*. An alternative explanation could be that UPS impairment leads to a global increase in the number of DSBs resolved as crossovers. However, even in this scenario, the efficient operation of crossover interference would be expected to strongly suppress the occurrence of closely spaced multiple crossovers on the same chromosome (Fig 6B). Therefore, while we cannot formally rule out a general increase in crossover number, our observation of multiple crossovers without clear spacing constraints more strongly suggests a concomitant defect in the crossover interference mechanism.

Our results demonstrate that the UPS regulates crossover patterning through at least two mechanisms. The first mechanism involves the regulation of pro-crossover protein abundance. Our data showed increased amount of pro-crossover proteins upon knockdown of the UPS. Correspondingly, we observed enlarged foci of the pro-crossover proteins during late pachytene suggesting that the UPS was involved in controlling the abundance of the pro-crossover proteins and restricting their chromosomal accumulation at the crossover designation site. Protein abundance has been linked to crossover control. In *Arabidopsis*, HEI10 dosage directly modulates crossover interference patterns. Elevated HEI10 level by overexpression was found to be accompanied by increased crossover formation [9,63]. In contrast, haploinsufficiency of both HEI10 and its interactor RNF212 disrupts normal crossover patterning [27,28]. Moreover, a recent study reported that regulation of the protein abundance of HEI10 by J3, a co-chaperone related to HSP40, can also modulate crossover interference [65], further linking protein abundance to crossover interference control. Our quantitative analysis revealed that UPS perturbation not only enhanced the fluorescence intensity of COSA-1 foci but also led to increased number of crossover-designated sites, indicative of compromised control of crossover interference. These findings extend previous observations of pro-crossover factor dosage sensitivity across species and reveal a conserved mechanism in which pro-crossover protein dosage fine-tunes crossover interference.

Our findings reveal that UPS-mediated regulation of pro-crossover protein abundance operates independently of crossover differentiation, as it remains functional even in the absence of DSB formation. This suggests that the UPS controls total protein levels prior to crossover designation (Fig 8). According to the coarsening model, maintaining a diffusible nuclear pool of pro-crossover proteins before crossover formation is critical. Excess protein increases the amount available for initiation of coarsening along the SC, directly elevating crossover numbers. One potential mechanism is that more proteins require a longer duration for coarsening. If coarsening is constrained to a specific time window, such as during pachytene, failure to complete the process before progression to the next meiotic stage could lead to the erroneous designation of multiple crossovers. In the meantime, during coarsening, different sites compete for limited pro-crossover factors. UPS knockdown, which elevates all pro-crossover proteins, may fundamentally alter this competitive landscape. Consequently, instead of only high-affinity sites winning out, the sudden abundance of pro-crossover proteins may allow many otherwise subthreshold sites to reach the threshold required for crossover designation, thereby weakening interference.

Previous studies have shown that altering the dosage of a single protein, such as HEI10 overexpression or haploinsufficiency, can perturb crossover interference [10,12,63,65]. By causing aberrant protein stabilization or aggregation, elevated level of one protein can promiscuously recruit downstream factors, thereby triggering concurrent crossover maturation at multiple loci. Our study implicates that the UPS acts as an upstream hub that coordinately regulates the entire network of pro-crossover proteins. This shifts the paradigm from individual effector dosage to integrated network control, suggesting that interference is not merely a spatial process driven by diffusion and competition, but also a biologically regulated process under precise temporal control and proteostatic governance.

Our study further reveals a crucial role for the interactions among pro-crossover proteins in enforcing crossover interference. The *cosa-1*^*PD*^ mutants present an intriguing phenotype: while the overall recombination frequency is reduced due to defective crossover formation, the incidence of double crossovers is increased (Fig 6A). This indicates that the weakened interactions between COSA-1 and MSH-5 or ZHP-3 specifically impair crossover interference. We propose that a strong and stable network of pro-crossover protein interactions is essential for the rapid and orderly progression of the coarsening process. Weakened protein interactions slow the dynamics of coarsening, preventing the system from completing the ‘winner-takes-all’ selection within the limited temporal window. Consequently, some pro-crossover proteins, failing to be fully recruited to the designated crossover site, may become stranded at other DSB sites that are normally destined for non-crossover fate, erroneously initiating additional crossovers. Thus, our data provide the first evidence that the crossover interference mechanism requires not only optimal protein abundance but also adequate interaction strength among pro-crossover complex components.

The second mechanism by which the UPS regulates crossover patterning involves actively controlling the chromosomal localization of pro-crossover factors. In late pachytene oocyte nuclei with UPS knockdown, we observed MSH-5-only and COSA-1-only foci, which were rarely observed in controls (S3D and S3E Fig). This suggests that during crossover designation, the UPS may facilitate the clearance of pro-crossover proteins from recombination intermediates that lack the potential to develop into a crossover. A successfully designated crossover site might actively recruit the UPS to degrade local pro-crossover proteins, thereby physically eliminating competing material from adjacent regions and reinforcing interference. UPS knockdown impairs this local clearance, causing a surplus of proteins to be mislocalized, leading to aberrant initiation at multiple sites and consequently, weakened interference. The UPS thus likely ensures the spatiotemporal precision of the coarsening process by mediating the timely degradation and clearance of pro-crossover factors.

Consistent with this, the rescue of COSA-1^PD^ accumulation at late pachytene upon UPS knockdown provides further experimental evidence that the UPS regulates the chromosomal localization of pro-crossover proteins. Although COSA-1^PD^ foci were undetectable cytologically, crossover formation still occurs between some homologs, suggesting that COSA-1 was initially recruited to the crossover-designated sites but failed to accumulate due to weakened interactions with other pro-crossover proteins, leading to dissociation or degradation. However, we cannot exclude the possibility that increased pro-crossover protein abundance upon UPS knockdown also contributes to the extra COSA-1 marked crossover-designated sites and/or to COSA-1^PD^ foci recovery. Although these two possibilities are not mutually exclusive, our data suggest that these functions could be separated. This notion is supported by our findings that knockdown of *npl-4*, which had no effect on COSA-1 abundance (S5D and S5E Fig), could nonetheless rescue the crossover formation defects in *cosa-1*^*PD*^ mutants (Fig 5C and 5D). The suppression of crossover defects in *cosa-1*^*PD*^ by *npl-4* RNAi is unlikely operating by correcting the intrinsic deficiency of COSA-1^PD^, as COSA-1^PD^ foci at late pachytene was not restored (S6D Fig). Instead, we propose another explanation that by inhibiting the timely dissociation of pro-crossover complexes, which could be initiated by ubiquitination as described earlier, *npl-4* RNAi may prolong the chromosomal residency of the functionally compromised COSA-1 protein, allowing it more opportunity to complete crossover designation. Taken together, our findings indicate that both the abundance and the precise chromosomal localization of pro-crossover proteins are critical for determining the number and distribution of crossovers. The UPS functions as a central integrator that coordinates these two regulatory layers, ensuring accurate crossover patterning.

Last but not the least, our findings reveal distinct yet coordinated roles for proteasomal degradation and ubiquitination in regulating pro-crossover factors during meiotic crossover formation. While both pathways influence crossover designation and formation (Figs 2 and 5), only ubiquitination seems to be required for the removal of pro-crossover proteins from the crossover sites during diakinesis (Figs 3 and 4). A well-established example of ubiquitination-mediated complex disassembly is that of the replisome. In that setting, polyubiquitination of MCM7 (a component of the CMG helicase) primes the replisome for CDC-48^UFD-1/NPL-4^-mediated disassembly at the end of DNA replication [66]. Our findings in this study indicates that polyubiquitin chains on one or more pro-crossover factors may similarly be recognized by CDC-48^UFD-1/NPL-4^ to trigger their dissociation from crossover sites. While we cannot formally exclude that residual proteasome activity after *rpn-6* and *pbs-6* RNAi treatment could be enough for pro-crossover factor dissociation, our data indicate a distinct role for ubiquitination in this process. In *C. elegans*, two spatially segregated proteasome populations have been identified: one localizes along the chromosome axis [32] and the other at the perichromosomal periphery [41]. We propose that axis-associated proteasomes may degrade pro-crossover protein aggregates at non-crossover sites, thereby maintaining crossover interference. In contrast, the peripheral/nucleoplasmic proteasome likely mediates the clearance of the disassembled pro-crossover complexes during diakinesis. Future studies should identify the specific E3 ligases and ubiquitination targets involved in these processes. Notably, SKR-1 and SKR-2, two core components of the SCF E3 complex, have recently been identified as structural components of the SC in *C. elegans* [67]. It would therefore be worth testing the potential effect of the SCF E3 ligases on the regulation of pro-crossover proteins during crossover designation. In particular, our ubiquitinomics study has revealed that a number of meiotic proteins were modified by ubiquitination, with part of them exhibiting changes in protein level. Future studies are needed to clarify the type of ubiquitination and the way how poly-ubiquitination is formed, reveal the spatial-temporal regulation of these modifications, and investigate their corresponding biological significances during meiosis.

## Materials and methods

### *C. elegans* strains and maintenance

All strains in this study were maintained on NGM (nematode growth medium) plates seeded with OP50 bacteria. All experiments were performed at 20°C. All *C. elegans* strains were derived from a Bristol N2 background. S4 Table summarizes all strains and mutations used in this study. *cosa-1::3 × HA*, *ollas::cosa-1*^*PD*^*::3 × FLAG* and *spo-11* deletion were generated by CRISPR-Cas9 genome editing. Briefly, worms were injected with CRISPR/Cas9 injection mixture (10 μl volume), including 0.2 μl Cas9 (IDT, 10 μg/μl stock), 1 μg sgRNA, 10 ng/μl pCFJ90 and 10 ng/μl pCFJ104 injection marker plasmids, and a melted dsDNA donor cocktail (PCR donor with blunt ends or single stranded overhangs, 30 ng/μl) [68,69] or single-stranded templates (30 ng/μl) in the final injection mixture as the repair template. Gene editing-related crRNAs, repair templates, and primers for genotyping are shown in S5 Table. Progenies were genotyped by PCR to detect edits and subsequently validated by sequencing. The strains were outcrossed with N2 at least 3 times prior to analysis.

### Analyses of brood size, progeny viability and incidence of males

L4 hermaphrodites were individually picked onto NGM plates (4 worms per plate, 3 plates total). The worms were transferred to fresh plates every 12 h, and the number of eggs were recorded for each plate until cessation of reproduction. The total egg output across all plates was averaged to determine the mean brood size per worm. The percentage of progeny viability was calculated as the total number of viable progenies divided by the total number of eggs. Unhatched embryos were counted 24 h after each worm transfer. The incidence of males was determined by dividing the number of males by the total number of hatched offspring.

### DIA proteomics

Whole worm samples were ground into powder using liquid nitrogen. Approximately 20 mg of powder were resuspended in 200 μl lysis buffer (4% SDS, 150 mM Tris-HCl pH 8.0, 5 mM EDTA with protease inhibitors). The samples were boiled for 3 min and further ultrasonicated. Undissolved cellular debris were removed by centrifugation at 16,000*g* for 20 min. The supernatant was collected and quantified with a BCA Protein Assay Kit. DTT was added to a final concentration of 100 mM and boiled for 5 min. 200 µl UA buffer (8 M Urea, 150 mM Tris-HCl, pH 8.0) was then added to the samples and the samples were then transferred into 1.5 ml Nanosep tube (Pall Corporation, MWCO 10K) and centrifuged for 20 min at 14,000 *g* at 20°C to remove DTT. 100 µl 0.05 M iodoacetamide (IAA) in UA buffer was added to block reduced cysteine residues and the samples were incubated for 30 min in darkness and then centrifuged for 10 min at 12,000*g*. After 2× wash with 100 µl UA buffer and 100 µl 50 mM NH_4_HCO_3_, respectively, 40 µl Trypsin buffer (6 µg Trypsin in 40 µl 50 mM NH_4_HCO_3_) was added into the sample and incubated at 37°C for 20 h. The peptides were harvested by centrifugation, acidified by 0.1% TFA, desalted with the C18 stage tip, and subsequently dried by a refrigerated CentriVap concentrator. Finally, the tryptic digested peptides were re-dissolved with 0.1% FA. The concentrations of re-dissolved peptides were determined by Nanodrop One device. LC–MS/MS were performed on a Orbitrap Astral mass spectrometer coupled with Vanquish Neo UHPLC system (Thermo Fisher Scientific). Mass spectra were acquired in the DIA mode and the DIA MS data were analyzed using DIA-NN 1.8.1. The MS data were then searched against the *Caenorhabditis elegans* reference proteome database uniprotkb-*Caenorhabditis elegans*[6239]-27445-20241018.fasta. Volcano plot and scatter plots in Figs 1C and S1A were executed with R package ggplot2. Proteins that met the criteria of fold change greater than 1.5-fold and *P* value less than 0.05 were considered as significant differentially expressed proteins. GO enrichment analyses were carried out with the Fisher’s exact test, and FDR correction for multiple testing was also performed. Enriched GO was nominally statistically significant at the Fisher’s exact test *P* < 0.01. Sample preparation and MS analysis were conducted by Shanghai Bioprofile Technology Company (Shanghai, China).

### Ubiquitin-modified proteome analysis

Isolated worm nuclei were used for the analysis. Following RNAi treatment, the adult worms were mixed with small shards of broken glass coverslips and shredded by vortexing in 15 ml conical tubes containing ice-cold nuclear isolation buffer (10 mM HEPES at pH 7.6, 1 mM EGTA, 1.5 mM MgCl_2_, 10 mM KCl, 250 mM sucrose, 0.5 mM DTT, and protease inhibitor cocktail). Worm debris was removed by centrifugation at 30*g* for 5 min, and the supernatant containing nuclei was filtered first with a 100 µm mesh, followed by a second filtration with a 40 µm mesh. The nuclei were then collected by centrifugation at 2,500*g* for 10 min. Protein preparation steps from grounding to the addition of IAA to block reduced cysteine residues are the same as in the DIA proteomics protocol. After 30 min in darkness, cold acetone was added, and the samples were left in −20°C to precipitate protein overnight. The samples were then centrifuged for 10 min at 12,000*g*, and the pellets were washed with cold acetone, centrifuged again, and air-dried. 8 M UA buffer was added to re-dissolve the precipitated protein. Next, 50 mM NH_4_HCO_3_ was added to dilute UA to less than 1 M. Trypsin was then added into the samples, at the enzyme-to-protein ratio of 1:25 (W:W). After incubation at 37°C for 20 h, the samples were acidified by 0.1% TFA, desalted with Sep-PAK, and dried by a refrigerated CentriVap concentrator (Labconco). The trypsin-digested samples were reconstituted and the ubiquitin-modified peptides were enriched with PTMScan Ubiquitin Remnant Motif (K-ε-GG) Kit (Cell Signaling Technology, cat. no. 5562). After wash with IPA buffer, the modified peptides were eluted with 0.15% (v/v) TFA. The elution of the modified peptides was then desalted with a C18 Stage Tips column and dried by a refrigerated CentriVap concentrator (Labconco). Finally, the modified peptides were re-dissolved with 0.1% FA. LC-MS/MS were performed on a Orbitrap Astral mass spectrometer coupled with Vanquish Neo UHPLC system (Thermo Fisher Scientific). Mass spectra were acquired in the DIA mode, and the DIA MS data were analyzed using Spectronaut 19 (Biognosys AG). The MS data were then searched against the *Caenorhabditis elegans* reference proteome database uniprotkb-*Caenorhabditis elegans*[6239]-27445-20241018.fasta. Sample preparation and MS analysis were conducted by Shanghai Bioprofile Technology Company (Shanghai, China).

### Immunoblot

Synchronized adult worms were washed into M9 buffer and lysed by adding 5×SDS sample buffer and heating to 95°C for 10 min with intermittent vortexing. Whole worm lysates were separated by SDS-PAGE and transferred onto PVDF membranes (Millipore IPVH00010). Membranes were blocked in 5% milk in PBST for 1 h at room temperature and incubated with primary antibodies overnight at 4°C. Primary antibodies used are as follows: mouse anti-HA (1:1,000, BioLegend, 16B12), rabbit anti-GFP (1:2,000, ABclonal Technology, AE011), and mouse anti-ACTIN (1:3,000, Proteintech, #66009-1-Ig).

### Immunofluorescence

Gonads from young adult hermaphrodites (48 h post L4 at 20°C) were dissected and stained as previously described [14]. Briefly, young adult hermaphrodites were dissected in 7 μl dissection buffer (25 mM HEPES pH 7.4, 118 mM NaCl, 48 mM KCl, 2 mM EDTA, 5 mM EGTA, 0.1% Tween-20) and briefly fixed in 1% formaldehyde. The dissected gonads were placed on a slide, covered with a coverslip, and flash-frozen in liquid nitrogen. The coverslip was quickly removed and the slide containing the gonads was then placed in −20°C methanol for 10 min. The slide was next washed three times in 1× PBST (0.1% Tween-20) and blocked with 1% BSA for 30 min at room temperature. The sample was then incubated with primary antibodies overnight at 4°C at the indicated dilutions as listed below.

Chromosome spreading of *C. elegans* germ cell nuclei was performed as previously described [14]. The gonads of adult worms, treated with RNAi for 48 h, were dissected in 5 μl low solution (0.2× PBS with 0.1% v/v Tween-20) on a 18 × 18 mm coverslip (ZEISS, thickness 0.170 ± 0.005 mm). Fifty microliters of spreading solution was then added and the dissected gonads were immediately distributed over the whole coverslip. Coverslips were put in 6 cm culture plates, left to dry overnight at room temperature, washed for 20 min in methanol at −20°C, and rehydrated by washing 3 times for 10 min in 1× PBST. After rehydrating, the samples were processed for immunofluorescence using antibodies at the concentrations listed below. Spreading solution (for one coverslip, 50 μl): 32 µl of fixative (4% w/v paraformaldehyde and 3.2 ± 3.6% w/v sucrose in water), 16 µl of Lipsol solution (1% v/v Lipsol in water) and 2 μl of sarcosyl solution (1% w/v of sarcosyl in water, Sigma-Aldrich L7414).

Primary antibodies used are as follows: mouse anti-FLAG (1:800, Sigma, F1804), GFP booster (1:400, Chromotek, gb2AF488), mouse anti-HA (1:500, BioLegend, 16B12), rabbit anti-HIM-3 (1:300, generated by ABclonal Technology) [14], guinea pig anti-HTP-1 (1:300, generated by ABclonal Technology) [14], and rabbit anti-SYP-1 (1:300, generated by ABclonal Technology) [14]. Secondary antibodies (Invitrogen Alexa 488 and Alexa 594) and DAPI (Sangon Biotech E607303) were used at 1:1,000 and 1:20 dilution, respectively. The samples were mounted in antifade mounting medium (Vectashield H-1000) and imaged using Olympus SpinSR10 with a 60× oil immersion and 1.42 NA objective.

### Meiotic crossover recombination frequency assay

Meiotic crossover recombination frequencies were determined as previously described [14], using six insertion–deletion polymorphisms on Chromosome Ⅱ that differ between N2 Bristol and CB4856 Hawaiian. *ollas::cosa-1*^*PD*^*::3 × FLAG* was crossed into a genetic background with Chr Ⅱ homozygous for Hawaiian DNA. Hawaiian males or homozygous *cosa-1*^*PD*^ mutant males with Chr Ⅱ homozygous Hawaiian were then crossed with hermaphrodites of the identical genotype in the N2 Bristol background to obtain mutant F1 strains heterozygous for Hawaiian. The F1 hermaphrodites were treated with either ctrl RNAi or 20% *uba-1* RNAi for 24 h and then crossed with males of CB5584, a *myo-2*::GFP expressing strain, and allowed to lay eggs. After confirming the genotype of the F1s, the F2 offspring with high level of GFP signals in the pharyngeal muscles were analyzed for crossover recombination by PCR. Primers used are as follows: Chromosome II:

genetic position (gp) -15.4 B5: primer 1:5′-AACGACGCGATGCTATGGAT-3′, primer 2:5′-TGGAATTGAAACAGAACTCAGC-3′, N2: 1,100 bp, CB4856:824 bp;
gp -6.3 B7: primer 1:5′-ATTTGGGTGGGAACTGGAGG-3′, primer 2:5′-GCGTGCAGACATAAGATAGGG-3′, N2:634 bp, CB4856:425 bp;
gp 0.1 B10: primer 1:5′-ACCAGCAATAGGTCAAGGTCT-3′, primer 2:5′-CACGTCATTCGCCAGTCAAA-3′, N2: 819 bp, CB4856:484 bp;
gp 5.7 C3: primer 1:5′-ACATGGGAGCGACGGTTTTA-3′, primer 2:5′-CCCGACACCATAACACAACA-3′, N2:933 bp, CB4856:448 bp;
gp 17.5 C5: primer 1:5′-AGCCGTTACTCGCCATGAAA-3′, primer 2:5′-GCCAAACATCGGTCATCGGA-3′, N2:959 bp, CB4856:744 bp;
gp 23.1 C6: primer 1:5′-TTGTGTGCAAACACCGTCAC-3′, primer 2:5′-TCGGTCCGAAGGCAATCAAA-3′, N2:1220 bp, CB4856:811 bp.

### RNAi, partial RNAi, and double RNAi

Feeding RNAi was used to achieve gene knockdown in *C. elegans*. The corresponding cDNAs were cloned into L4440 to construct the RNAi plasmid. The empty L4440 was used as control. The RNAi plasmid was transferred to *E. coli* HT115, which had tetracycline resistance. Single colonies were grown overnight in LB liquid medium containing ampicillin (Amp) and tetracycline (Tet) (both at 25 μg/ml final concentration) at 37°C and 225 rpm. The overnight bacterial cultures were transferred to fresh LB (+Amp, +Tet) at 1:50 (v/v) and incubated at 37°C until a value of optical density at 600 nm (OD_600_) between 0.8 and 1 was reached. 3 mM IPTG was then added to induce dsRNA expression for 6–8 h at 20°C and 160 rpm. The induced bacterial cultures were then spread onto NGM plates supplemented with 2 mM IPTG, 25 μg/ml Amp, and 25 μg/ml Tet. L4-stage worms were transferred to the prepared RNAi plates and left at 20°C for 48 h, unless otherwise stated, before analysis.

For *uba-1* partial RNAi, after obtaining bacterial cultures (empty L4440 control and *uba-1* RNAi, respectively) with identical OD_600_ values, IPTG was added to induce dsRNA expression. The IPTG-induced bacterial cultures were then mixed at predetermined ratios before being applied to IPTG plates to achieve titrated gene silencing. For 50% *syp-1* and 50% *uba-1* double RNAi, we mixed the IPTG-induced bacterial cultures at a 1:1 ratio before seeding. For *rpn-6* and *pbs-6* double RNAi, cDNAs of both *rpn-6* and *pbs-6* were cloned into the same L4440 plasmid. The remaining steps were identical to the standard RNAi procedures.

### RNA-seq

Total RNA was extracted using Trizol (Thermo Fisher Scientific) and further treated with DNase to remove genomic DNA contamination. Isolation of mRNA was performed using the KAPA Stranded mRNA-Seq Kit (Roche Diagnostics GmbH), and the mRNA was then used for RNA-Seq library preparation with the KAPA Stranded mRNA-Seq Kit for Illumina (Roche Diagnostics GmbH). The library was then subjected to Illumina sequencing with paired-end 2 × 150 as the sequencing mode. After quality control, the clean reads were mapped to the *C. elegans* genome (assembly WBcel235) using the HISAT2 v2.1.0 (non-default parameters: --rna-strandness RF --dta) software. Gene expression levels were estimated using FPKM by StringTie v1.3.4d (non-default parameters: -e --rf). edgeR v3.24.2 was used to measure differential gene expression. The false discovery rate (FDR) control method was used to calculate the adjusted *P* values in multiple testing to evaluate the significance of the differences. Only gene with an adjusted *P* value <0.05 and |log2FC| ≥ 1 were used for analysis. Library preparation, sequencing, and data analysis were performed by Genefund Biotech (Shanghai, China).

### Yeast two-hybrid analysis

The yeast two-hybrid assay was performed as previously described [70]. Full-length coding sequences for pro-crossover factors (COSA-1, MSH-5, and CDK-2) and COSA-1 mutants (COSA-1^PD^) were cloned into plasmid pGADT7 or pGBKT7. Each pair of bait and prey plasmids was co-transformed into yeast strain AH109. The positive colonies were selected on a medium lacking tryptophan (-Trp) and leucine (-Leu) and were resuspended in 1 ml 1× PBS. Serial dilutions of the resuspension were plated on -His -Trp -Leu and -Trp -Leu solid media, and the plates were incubated at 25°C for 3 or 4 days to view the results.

### Statistics

Prism 8 software (GraphPad) was used to generate graphs and perform statistical analyses. When it comes to the total immunofluorescence intensity of the germline, signals from the distal tip to the end of pachytene were quantified. *P* values were primarily calculated using an unpaired *t* test or ordinary one-way ANOVA. The significance of multiple crossover occurrence was assessed using a one-tailed Fisher’s exact test. *P* < 0.05 was considered significant. Sample sizes were not predetermined and experiments were not randomized. All the experiments for quantification were conducted at least three times.

## Supporting information

S1 Fig Knockdown of the UPS led to increased protein levels of pro-crossover factors. (**A**) Scatter blot showing changes in individual protein upon *uba-1* RNAi treatment as revealed by DIA proteomics. The scatter blot was executed with R package ggplot2. (**B**) Top 10 biological processes in which upregulated proteins by germline *uba-1* RNAi are enriched. The size of the circle represents protein count, and the color of circle represents rich factor. Rich factor = (*a*/*b*)/(*c*/*d*), in which *a* indicates the number of differential proteins annotated to a specific TERM, *b* indicates the total number of differential proteins annotated to the total TERM, *c* indicates the number of background proteins for the specific TERM, and *d* indicates the number of background proteins for the total TERM. (**C–E**) Western blot showing changes in protein levels of eGFP::HIM-6, GFP::MSH-5, GFP::SYP-3 and GFP::COSA-1 after RNAi treatment of *rpn-6*, *uba-1* or *mpk-1* as compared with ctrl RNAi. (**F**) RNA-seq showing no significant changes in the transcription of known pro-crossover proteins. (**G**) Immunofluorescence images of diakinesis nuclei from *ollas::cosa-1::3 × FLAG; spo-11*, stained for DAPI (blue) and HIM-3 (magenta), showing failure in CO formation. The underlying data for S1A, S1B, and S1F Fig can be found in S1 Table, S1 Data, and S2 Table, respectively.
(TIF)

S2 Fig The regulation of pro-crossover protein abundance by the UPS is not dependent on SPO-11. (**A**) Representative immunofluorescence images of nuclei at transition zone (TZ), early pachytene (EP), mid-pachytene (MP) and late pachytene (LP) stages in *cosa-1::3 × HA* and *zhp-3::AID::3 × HA* worms treated with ctrl, *rpn-6* or *uba-1* RNAi, stained for DAPI (blue), HIM-3 (magenta), and COSA-1 (green, upper panels) or ZHP-3 (green, lower panels). Yellow arrowheads point to crescent-shaped nuclei, and white arrows point to HIM-3 polycomplexes. (**B**) Immunofluorescence images of late pachytene nuclei from *ollas::cosa-1::3 × FLAG; spo-11*, treated with ctrl, *rpn-6* or *uba-1* RNAi, stained for DAPI (blue), HIM-3 (magenta), and COSA-1 (green). (**C**) Quantification of focus number of COSA-1 in (B). The means ± SD are shown. *P* values are given by ordinary one-way ANOVA. Scale bars, 5 μm for (A) and (B). The underlying data for S2C Fig can be found in S1 Data.
(TIF)

S3 Fig Effects of UPS knockdown on protein level of HTP-1 or SYP-1, and on colocalization between COSA-1 and MSH-5. (**A**) Immunofluorescence images of dissected gonad from *cosa-1::3 × HA*, treated with ctrl, *rpn-6* or *uba-1* RNAi, stained for DAPI (blue), HTP-1 (magenta) and SYP-1 (green). Scale bar, 100 μm. **(B, C**) Quantification of immunofluorescence intensity of HTP-1 and SYP-1 in (A). *n* = 3. (**D, E**) Quantification of the percentage of MSH-5 foci that colocalize with COSA-1 per nucleus (D) and the percentage of COSA-1 foci colocalizing with MSH-5 per nucleus (E). The number of nuclei quantified is provided. The means ± SD are shown. *P* values are given by ordinary one-way ANOVA. The underlying data for S3B–S3E Fig can be found in S1 Data.
(TIF)

S4 Fig Knockdown of the proteasome did not lead to persistent association of pro-crossover proteins with diakinesis chromosomes. (**A**) Western blot showing changes in protein level of COSA-1::3 × HA upon ctrl and *pbs-6* RNAi treatments. (**B**) Representative immunofluorescence images of dissected gonads from *cosa-1::3 × HA* after ctrl and *pbs-6* RNAi treatments. (**C**) Quantification of immunofluorescence intensity of COSA-1 in (B). The means ± SD are shown. *P* value is given by *t* test. *n* = 3. (**D**) Quantification of the percentage of diakinesis nuclei with chromosome-associated GFP::COSA-1 foci after RNAi treatment for ctrl, *uba-1* and *pbs-6*. (**E**) Representative immunofluorescence images of dissected gonads from *cosa-1::3 × HA* after ctrl, *rpn-6* and *rpn-6* + *pbs-6* RNAi treatments. The underlying data for S4C and S4D Fig can be found in S1 Data.
(TIF)

S5 Fig Knockdown of the CDC-48^UFD-1/NPL-4^ segregase delayed dissociation of pro-crossover proteins from diakinesis chromosomes. (**A**) Immunofluorescence images of diakinesis (−1, −2, and −3) nuclei from the *zhp-3::GFP* transgenic worms treated with ctrl, *cdc-48*, or *npl-4* RNAi, stained for DAPI (blue), HIM-3 (magenta), and ZHP-3 (green). (**B**) Quantification of the percentage of diakinesis (−1, −2, and −3) nuclei with chromosome-associated ZHP-3::GFP foci after RNAi treatment for ctrl, *cdc-48*, *npl-4* and *uba-1*. (**C**) Quantification of the percentage of diakinesis (−1, −2, and −3) nuclei with chromosome-associated GFP::COSA-1 foci after RNAi treatment for ctrl, *cdc-48* and *npl-4*. (**D**) Immunofluorescence images of dissected gonad from *cosa-1::3 × HA*, treated with ctrl RNAi or *npl-4* RNAi, stained for DAPI (blue), HIM-3 (magenta) and COSA-1 (green). (**E**) Quantification of immunofluorescence intensity of COSA-1 in (D). *n* ≥ 3. (**F**) Immunofluorescence images of dissected gonad from *cosa-1::3 × HA*, treated with ctrl RNAi or *npl-4* RNAi, stained for DAPI (blue), HIM-3 (magenta) and ZHP-3 (green). (**G)** Quantification of immunofluorescence intensity of ZHP-3 in (F). *n* ≥ 3. The means ± SD are shown. *P* values are given by *t* test. Scale bar, 5 μm for (A), 100 μm for (D) and (F). The underlying data for S5B, S5C, S5E and S5G Fig can be found in S1 Data.
(TIF)

S6 Fig Effects of *uba-1* partial RNAi and *npl-4* RNAi in *cosa-1*^*PD*^. (**A**) Immunofluorescence images of dissected gonad from *ollas::cosa-1*^*PD*^*::3 × FLAG*, treated with ctrl RNAi or 20% *uba-1* RNAi, stained for DAPI (blue), HIM-3 (magenta), and COSA-1 (green), showing effect of 20% *uba-1* RNAi on protein level of COSA-1. (**B**) Fluorescence images of late pachytene nuclei from *GFP::msh-5; mCherry::H2B* and *cosa-1*^*PD*^*; GFP::msh-5; mCherry::H2B* worms, showing mCherry::H2B (magenta) and GFP::MSH-5 (green). (**C**) Quantification of GFP::MSH-5 foci as shown in (B). *P* value is given by *t* test. (**D**) Immunofluorescence images of LP nuclei from *ollas::cosa-1*^*PD*^*::3 × FLAG*, treated with ctrl or *npl-4* RNAi, stained for DAPI (blue), HIM-3 (magenta), and COSA-1 (green). Scale bar, 100 μm for (A) and 5 μm for (B) and (D). The underlying data for S6C Fig can be found in S1 Data.
(TIF)

S7 Fig *uba-1* RNAi led to the appearance of ring-like bivalents. Immunofluorescence images of diakinesis nuclei from *ollas::cosa-1::3 × FLAG* and *ollas::cosa-1*^*PD*^*::3 × FLAG* treated with *uba-1* RNAi. The yellow arrows in the images denote the ring-like bivalent structures. Scale bar, 5 μm.
(TIF)

S8 Fig SYP-1 partial knockdown cannot rescue meiotic defects of *cosa-1*^*PD*^. (**A**) Immunofluorescence images of dissected gonad from *cosa-1::3 × HA* treated with ctrl RNAi and 50% *syp-1* RNAi, stained for DAPI (blue) and HIM-3 (magenta). (**B**) Representative DAPI-stained images of the distal part of the gonad upon different RNAi treatments, showing the effects of each RNAi treatment on chromosome morphology of the TZ region. (**C**) Immunofluorescence images of representative late pachytene nuclei from *cosa-1::3 × HA* worms with the indicated RNAi treatment, stained for DAPI (blue), HIM-3 (magenta) and COSA-1 (green). (**D**) Quantification of the percentage of late pachytene nuclei with indicated number of COSA-1::3 × HA foci as shown in (C). *P* value is given by *t* test. (**E**) Representative images of diakinesis nuclei showing normal bivalent formation upon 50% *syp-1* RNAi treatment (*n* = 11). (**F**) Immunofluorescence images of representative late pachytene nuclei from *ollas::cosa-1*^*PD*^*::3 × FLAG* worms with the indicated RNAi treatment, stained for DAPI (blue), HIM-3 (magenta) and COSA-1 (green). (**G**) Immunofluorescence images of diakinesis nuclei stained for HIM-3 (magenta) and DAPI (blue), showing bivalent formation in *ollas::cosa-1*^*PD*^*::3 × FLAG* worms treated with ctrl RNAi and 50% *syp-1* RNAi. (**H**) Quantification of the percentage of nuclei with the number of DAPI/HIM-3-stained bodies indicated in (G). Scale bars, 100 μm for (A), 50 μm for (B), 5 μm for (C) and (E–G). The underlying data for S8D and S8H Fig can be found in S1 Data.
(TIF)

S1 Raw Images Scanned films for Figs 1E, S1C–S1E, and S4A. (PDF)

S1 Data The underlying numerical data for Figs 1G, 1I, 1K, 2F, 3D, 4C, 4E, 5D–5G, 5I, S1B, S2C, S3B–S3E, S4C, S4D, S5B, S5C, S5E, S5G, S6C, S8D, and S8H. (XLSX)

S1 Table DIA proteomics data. (XLSX)

S2 Table Spreadsheet of all the differentially expressed genes resulted from *uba-1* RNAi as revealed by RNA-seq. (XLSX)

S3 Table Spreadsheet of all the reliable ubiquitination sites identified by the DIA ubiquitinomics. (XLSX)

S4 Table List of strains used in this study. (DOCX)

S5 Table crRNAs, repair templates and genotyping primers used in this study. (DOCX)

## Abbreviations

CO: Crossover
COI: crossover interference
DIA: data-independent acquisition
DSBs: double-strand breaks
FDR: false discovery rate
OD: optical density
SC: synaptonemal complex
SC-CR: synaptonemal complex central region
UPS: ubiquitin-proteasome system
