# Supplementary figures and images for: Ubiquitin-proteasome system regulates pro-crossover protein dynamics during meiosis in Caenorhabditis elegans
Source: PLoS Biol. 2026 Jun 16;24(6):e3003868. doi: 10.1371/journal.pbio.3003868 (PMC13293516; doi:10.1371/journal.pbio.3003868)

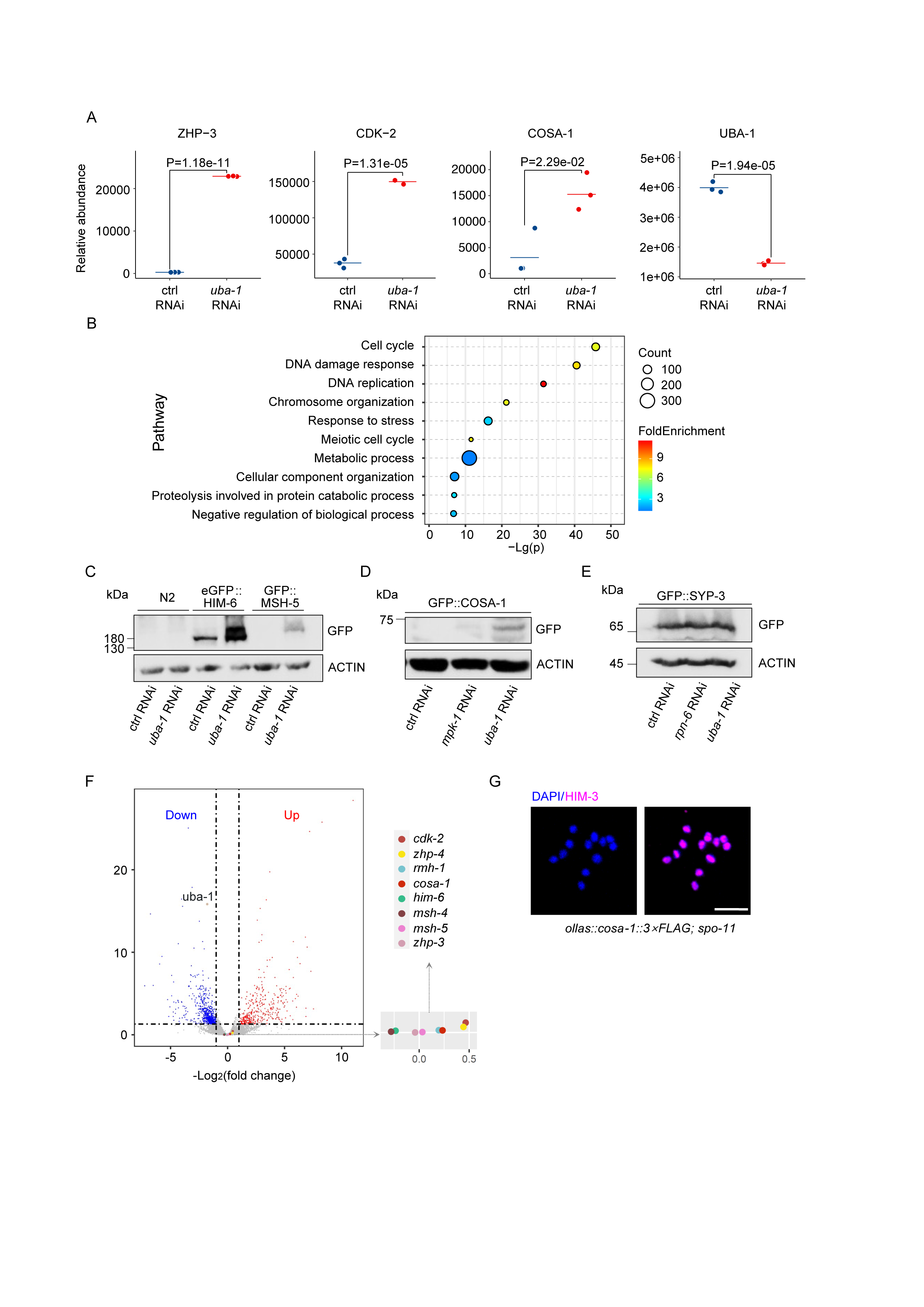

Supplement: S1 Fig — (A) Scatter blot showing changes in individual protein upon uba-1 RNAi treatment as revealed by DIA proteomics. The scatter blot was executed with R package ggplot2. (B) Top 10 biological processes in which upregulated proteins by germline uba-1 RNAi are enriched. The size of the circle represents protein count, and the color of circle represents rich factor. Rich factor = (a/b)/(c/d), in which a indicates the number of differential proteins annotated to a specific TERM, b indicates the total number of differential proteins annotated to the total TERM, c indicates the number of background proteins for the specific TERM, and d indicates the number of background proteins for the total TERM. (C–E) Western blot showing changes in protein levels of eGFP::HIM-6, GFP::MSH-5, GFP::SYP-3 and GFP::COSA-1 after RNAi treatment of rpn-6, uba-1 or mpk-1 as compared with ctrl RNAi. (F) RNA-seq showing no significant changes in the transcription of known pro-crossover proteins. (G) Immunofluorescence images of diakinesis nuclei from ollas::cosa-1::3 × FLAG; spo-11, stained for DAPI (blue) and HIM-3 (magenta), showing failure in CO formation. The underlying data for S1A, S1B, and S1F Fig can be found in S1 Table, S1 Data, and S2 Table, respectively. (TIF) [file pbio.3003868.s001.tif]

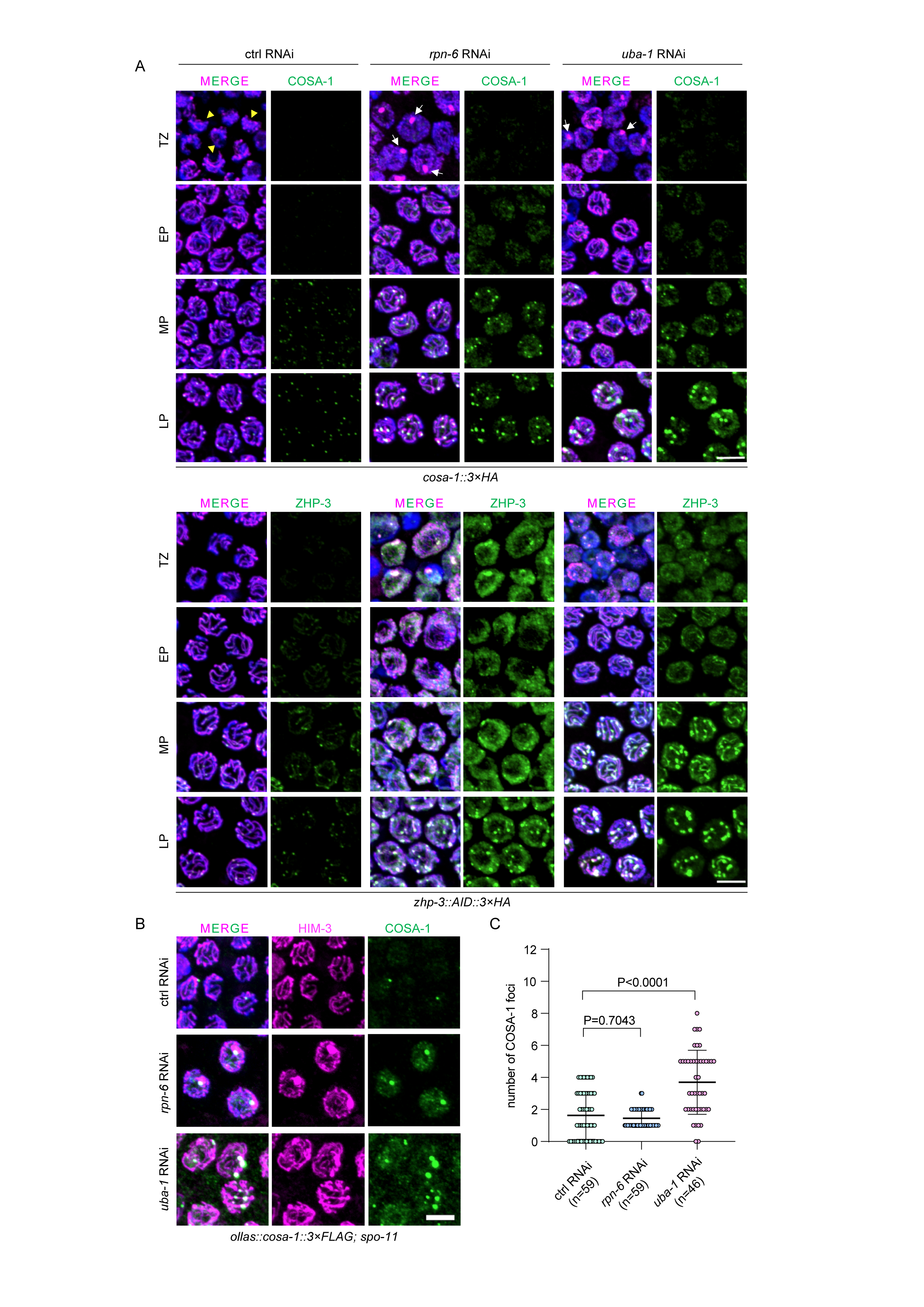

Supplement: S2 Fig — (A) Representative immunofluorescence images of nuclei at transition zone (TZ), early pachytene (EP), mid-pachytene (MP) and late pachytene (LP) stages in cosa-1::3 × HA and zhp-3::AID::3 × HA worms treated with ctrl, rpn-6 or uba-1 RNAi, stained for DAPI (blue), HIM-3 (magenta), and COSA-1 (green, upper panels) or ZHP-3 (green, lower panels). Yellow arrowheads point to crescent-shaped nuclei, and white arrows point to HIM-3 polycomplexes. (B) Immunofluorescence images of late pachytene nuclei from ollas::cosa-1::3 × FLAG; spo-11, treated with ctrl, rpn-6 or uba-1 RNAi, stained for DAPI (blue), HIM-3 (magenta), and COSA-1 (green). (C) Quantification of focus number of COSA-1 in (B). The means ± SD are shown. P values are given by ordinary one-way ANOVA. Scale bars, 5 μm for (A) and (B). The underlying data for S2C Fig can be found in S1 Data. (TIF) [file pbio.3003868.s002.tif]

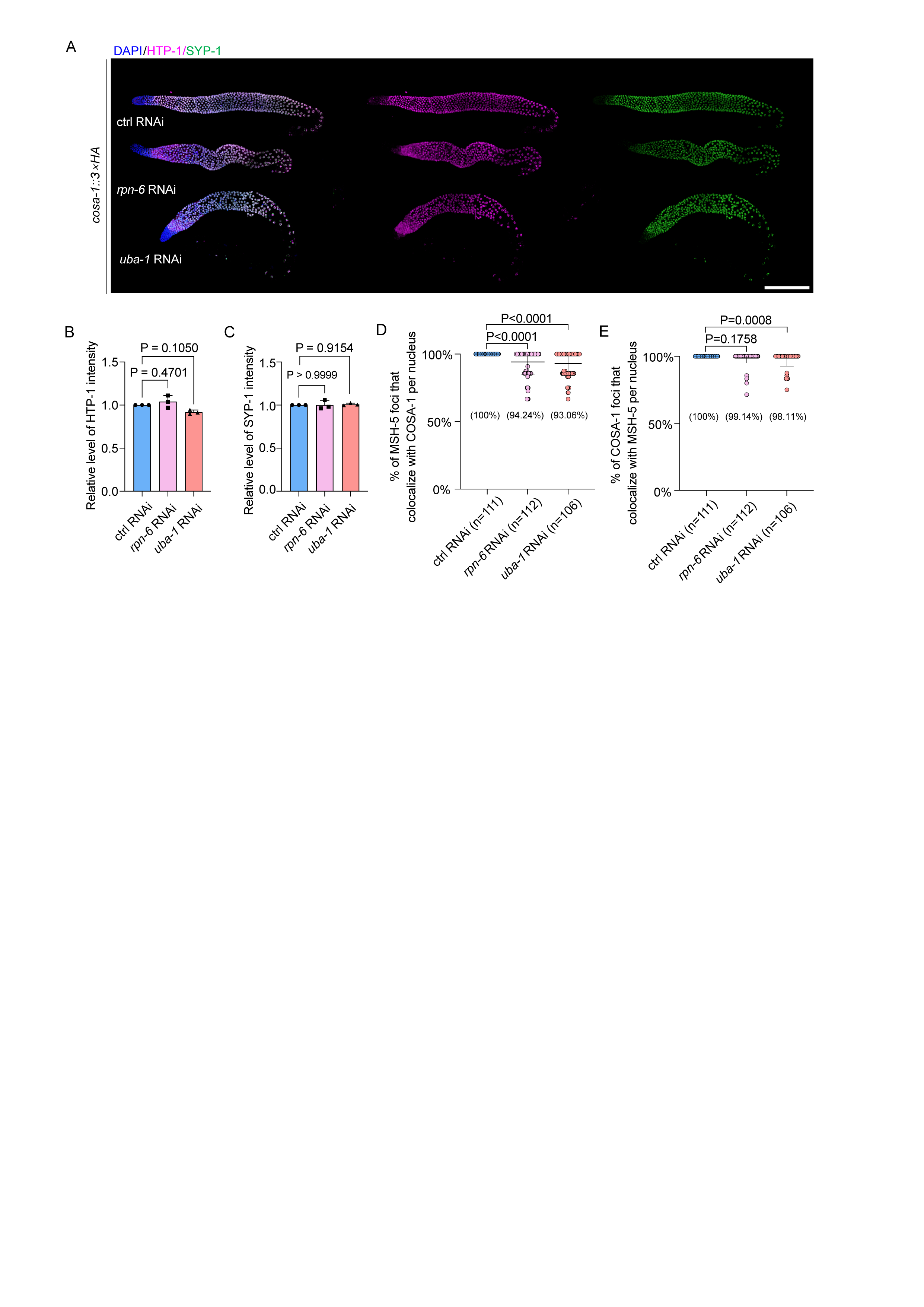

Supplement: S3 Fig — (A) Immunofluorescence images of dissected gonad from cosa-1::3 × HA, treated with ctrl, rpn-6 or uba-1 RNAi, stained for DAPI (blue), HTP-1 (magenta) and SYP-1 (green). Scale bar, 100 μm. (B, C) Quantification of immunofluorescence intensity of HTP-1 and SYP-1 in (A). n = 3. (D, E) Quantification of the percentage of MSH-5 foci that colocalize with COSA-1 per nucleus (D) and the percentage of COSA-1 foci colocalizing with MSH-5 per nucleus (E). The number of nuclei quantified is provided. The means ± SD are shown. P values are given by ordinary one-way ANOVA. The underlying data for S3B–S3E Fig can be found in S1 Data. (TIF) [file pbio.3003868.s003.tif]

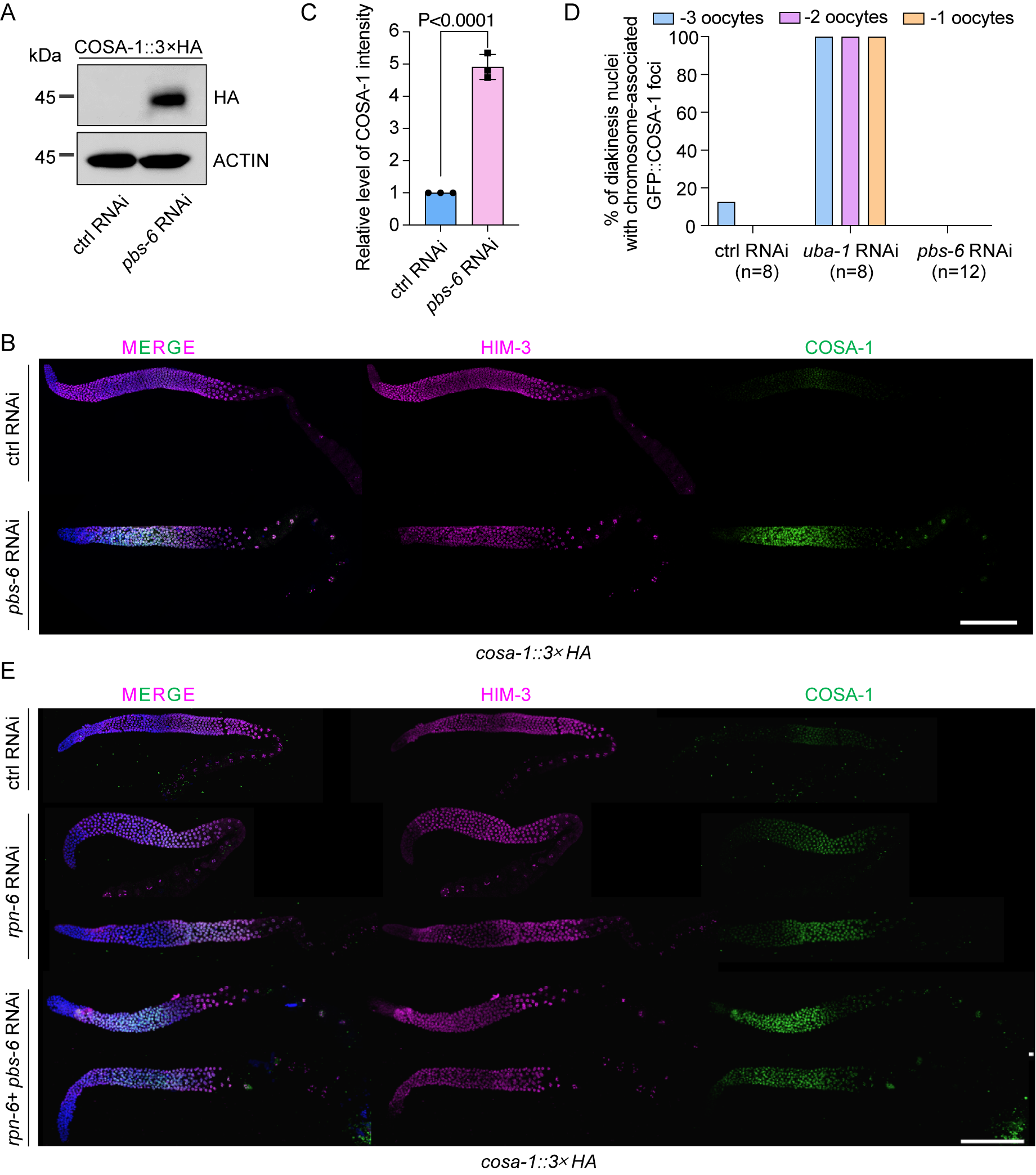

Supplement: S4 Fig — (A) Western blot showing changes in protein level of COSA-1::3 × HA upon ctrl and pbs-6 RNAi treatments. (B) Representative immunofluorescence images of dissected gonads from cosa-1::3 × HA after ctrl and pbs-6 RNAi treatments. (C) Quantification of immunofluorescence intensity of COSA-1 in (B). The means ± SD are shown. P value is given by t test. n = 3. (D) Quantification of the percentage of diakinesis nuclei with chromosome-associated GFP::COSA-1 foci after RNAi treatment for ctrl, uba-1 and pbs-6. (E) Representative immunofluorescence images of dissected gonads from cosa-1::3 × HA after ctrl, rpn-6 and rpn-6 + pbs-6 RNAi treatments. The underlying data for S4C and S4D Fig can be found in S1 Data. (TIF) [file pbio.3003868.s004.tif]

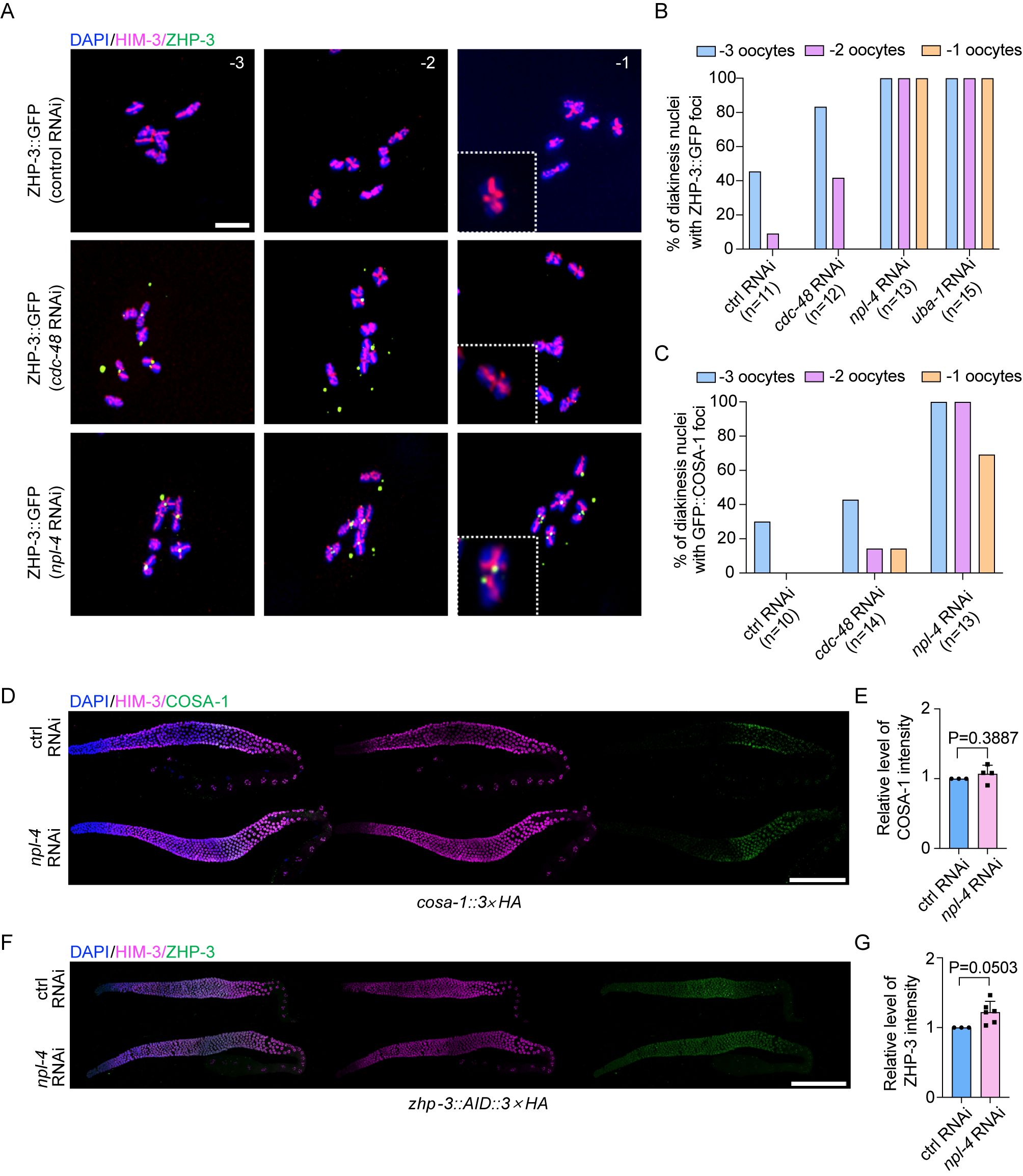

Supplement: S5 Fig — (A) Immunofluorescence images of diakinesis (−1, −2, and −3) nuclei from the zhp-3::GFP transgenic worms treated with ctrl, cdc-48, or npl-4 RNAi, stained for DAPI (blue), HIM-3 (magenta), and ZHP-3 (green). (B) Quantification of the percentage of diakinesis (−1, −2, and −3) nuclei with chromosome-associated ZHP-3::GFP foci after RNAi treatment for ctrl, cdc-48, npl-4 and uba-1. (C) Quantification of the percentage of diakinesis (−1, −2, and −3) nuclei with chromosome-associated GFP::COSA-1 foci after RNAi treatment for ctrl, cdc-48 and npl-4. (D) Immunofluorescence images of dissected gonad from cosa-1::3 × HA, treated with ctrl RNAi or npl-4 RNAi, stained for DAPI (blue), HIM-3 (magenta) and COSA-1 (green). (E) Quantification of immunofluorescence intensity of COSA-1 in (D). n ≥ 3. (F) Immunofluorescence images of dissected gonad from cosa-1::3 × HA, treated with ctrl RNAi or npl-4 RNAi, stained for DAPI (blue), HIM-3 (magenta) and ZHP-3 (green). (G) Quantification of immunofluorescence intensity of ZHP-3 in (F). n ≥ 3. The means ± SD are shown. P values are given by t test. Scale bar, 5 μm for (A), 100 μm for (D) and (F). The underlying data for S5B, S5C, S5E and S5G Fig can be found in S1 Data. (TIF) [file pbio.3003868.s005.tif]

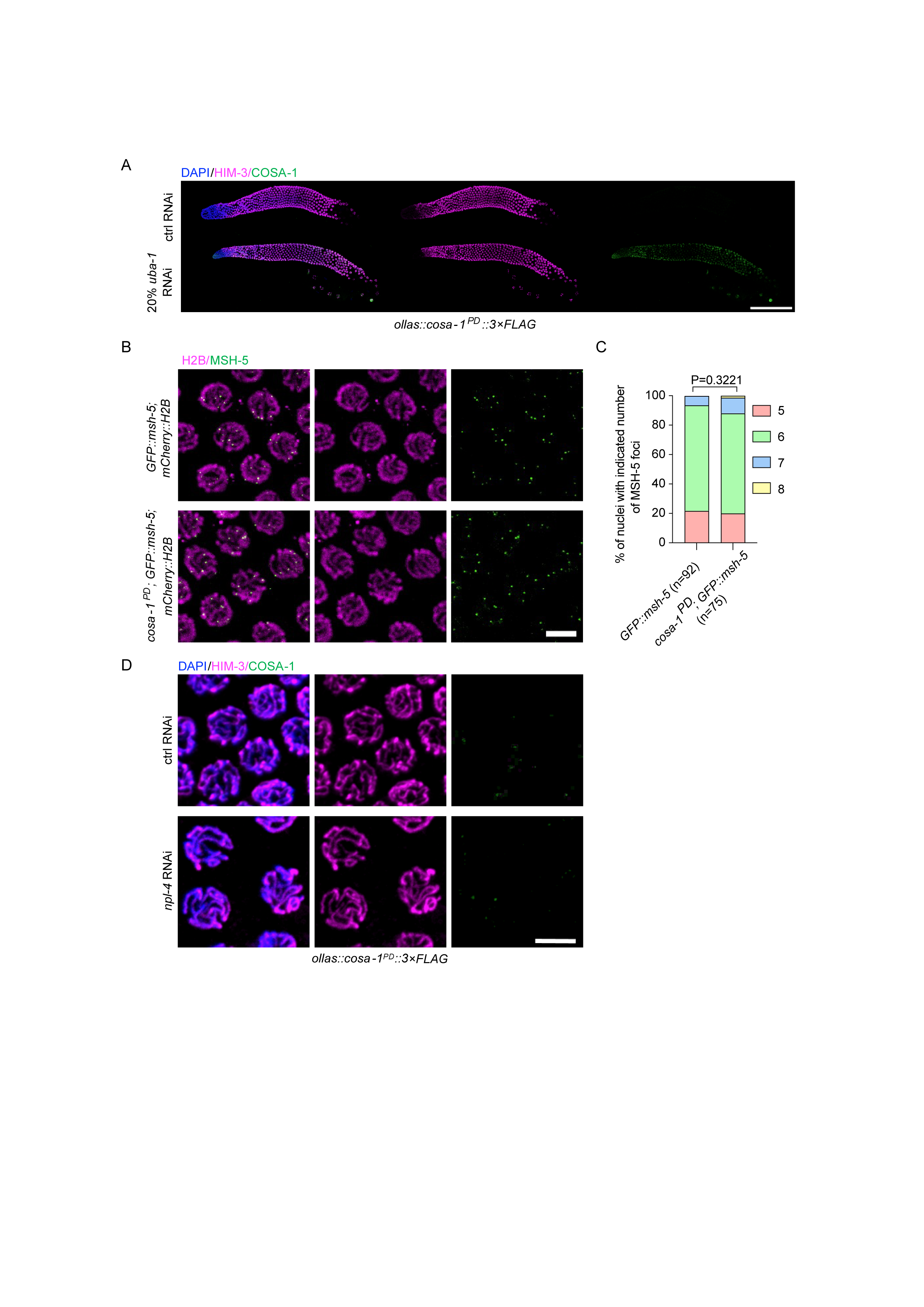

Supplement: S6 Fig — (A) Immunofluorescence images of dissected gonad from ollas::cosa-1PD::3 × FLAG, treated with ctrl RNAi or 20% uba-1 RNAi, stained for DAPI (blue), HIM-3 (magenta), and COSA-1 (green), showing effect of 20% uba-1 RNAi on protein level of COSA-1. (B) Fluorescence images of late pachytene nuclei from GFP::msh-5; mCherry::H2B and cosa-1PD; GFP::msh-5; mCherry::H2B worms, showing mCherry::H2B (magenta) and GFP::MSH-5 (green). (C) Quantification of GFP::MSH-5 foci as shown in (B). P value is given by t test. (D) Immunofluorescence images of LP nuclei from ollas::cosa-1PD::3 × FLAG, treated with ctrl or npl-4 RNAi, stained for DAPI (blue), HIM-3 (magenta), and COSA-1 (green). Scale bar, 100 μm for (A) and 5 μm for (B) and (D). The underlying data for S6C Fig can be found in S1 Data. (TIF) [file pbio.3003868.s006.tif]

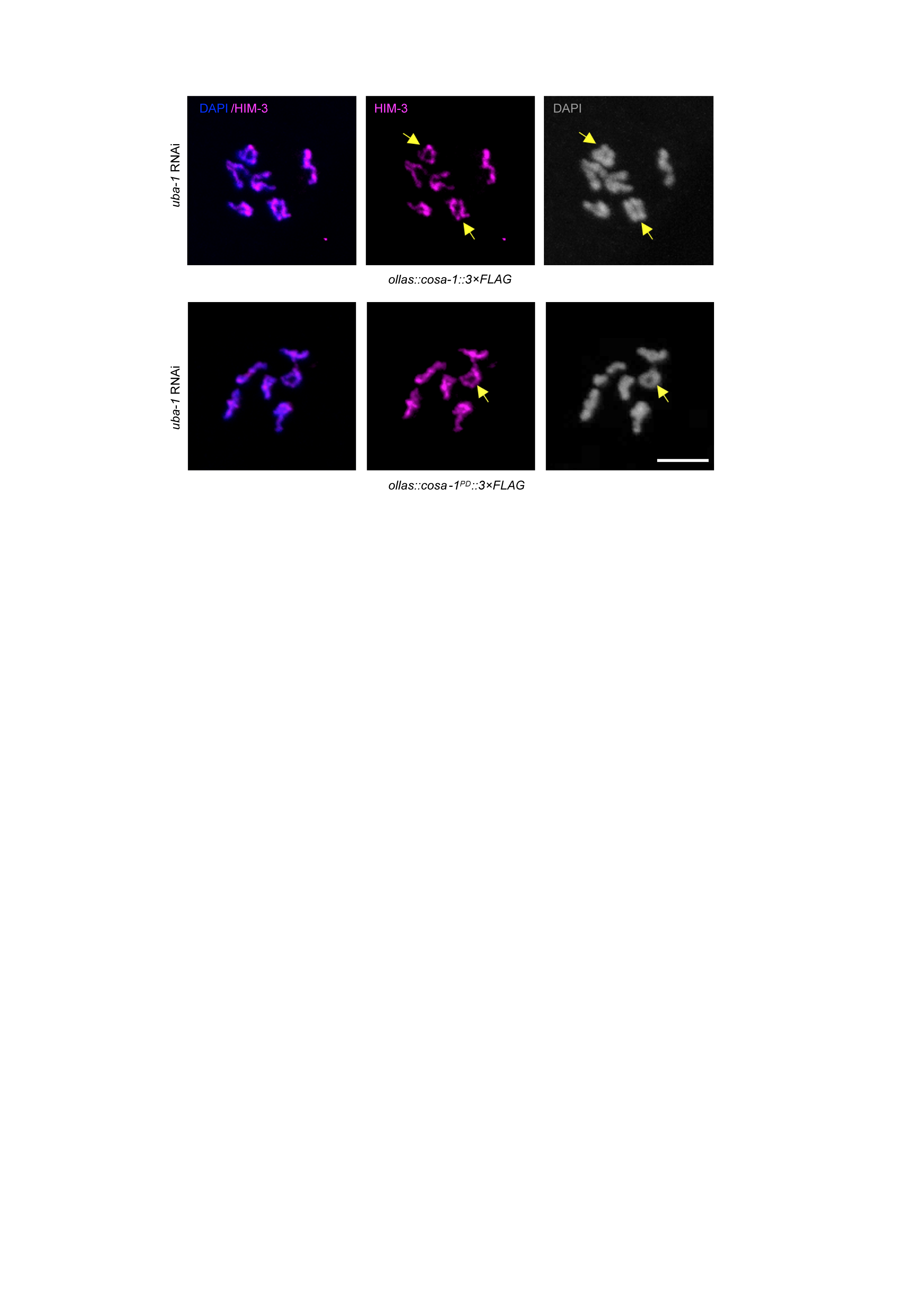

Supplement: S7 Fig — Immunofluorescence images of diakinesis nuclei from ollas::cosa-1::3 × FLAG and ollas::cosa-1PD::3 × FLAG treated with uba-1 RNAi. The yellow arrows in the images denote the ring-like bivalent structures. Scale bar, 5 μm. (TIF) [file pbio.3003868.s007.tif]

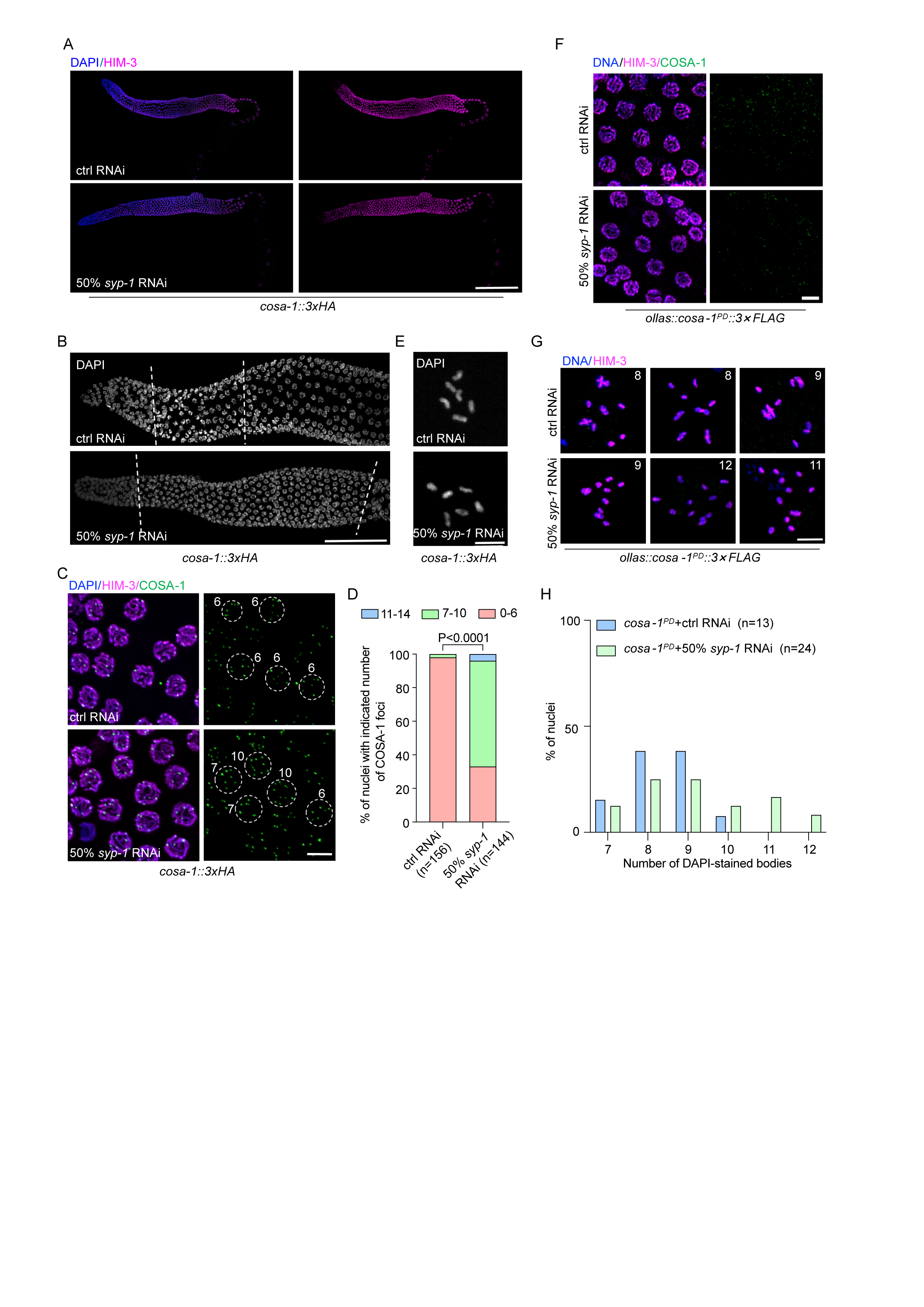

Supplement: S8 Fig — (A) Immunofluorescence images of dissected gonad from cosa-1::3 × HA treated with ctrl RNAi and 50% syp-1 RNAi, stained for DAPI (blue) and HIM-3 (magenta). (B) Representative DAPI-stained images of the distal part of the gonad upon different RNAi treatments, showing the effects of each RNAi treatment on chromosome morphology of the TZ region. (C) Immunofluorescence images of representative late pachytene nuclei from cosa-1::3 × HA worms with the indicated RNAi treatment, stained for DAPI (blue), HIM-3 (magenta) and COSA-1 (green). (D) Quantification of the percentage of late pachytene nuclei with indicated number of COSA-1::3 × HA foci as shown in (C). P value is given by t test. (E) Representative images of diakinesis nuclei showing normal bivalent formation upon 50% syp-1 RNAi treatment (n = 11). (F) Immunofluorescence images of representative late pachytene nuclei from ollas::cosa-1PD::3 × FLAG worms with the indicated RNAi treatment, stained for DAPI (blue), HIM-3 (magenta) and COSA-1 (green). (G) Immunofluorescence images of diakinesis nuclei stained for HIM-3 (magenta) and DAPI (blue), showing bivalent formation in ollas::cosa-1PD::3 × FLAG worms treated with ctrl RNAi and 50% syp-1 RNAi. (H) Quantification of the percentage of nuclei with the number of DAPI/HIM-3-stained bodies indicated in (G). Scale bars, 100 μm for (A), 50 μm for (B), 5 μm for (C) and (E–G). The underlying data for S8D and S8H Fig can be found in S1 Data. (TIF) [file pbio.3003868.s008.tif]
