# Supplementary material for: Ubiquitin-proteasome system regulates pro-crossover protein dynamics during meiosis in Caenorhabditis elegans
Source: PLoS Biol. 2026 Jun 16;24(6):e3003868. doi: 10.1371/journal.pbio.3003868 (PMC13293516; doi:10.1371/journal.pbio.3003868)
Supplement: S1 Raw Images — (PDF) [file pbio.3003868.s009.pdf]

Fig 1E

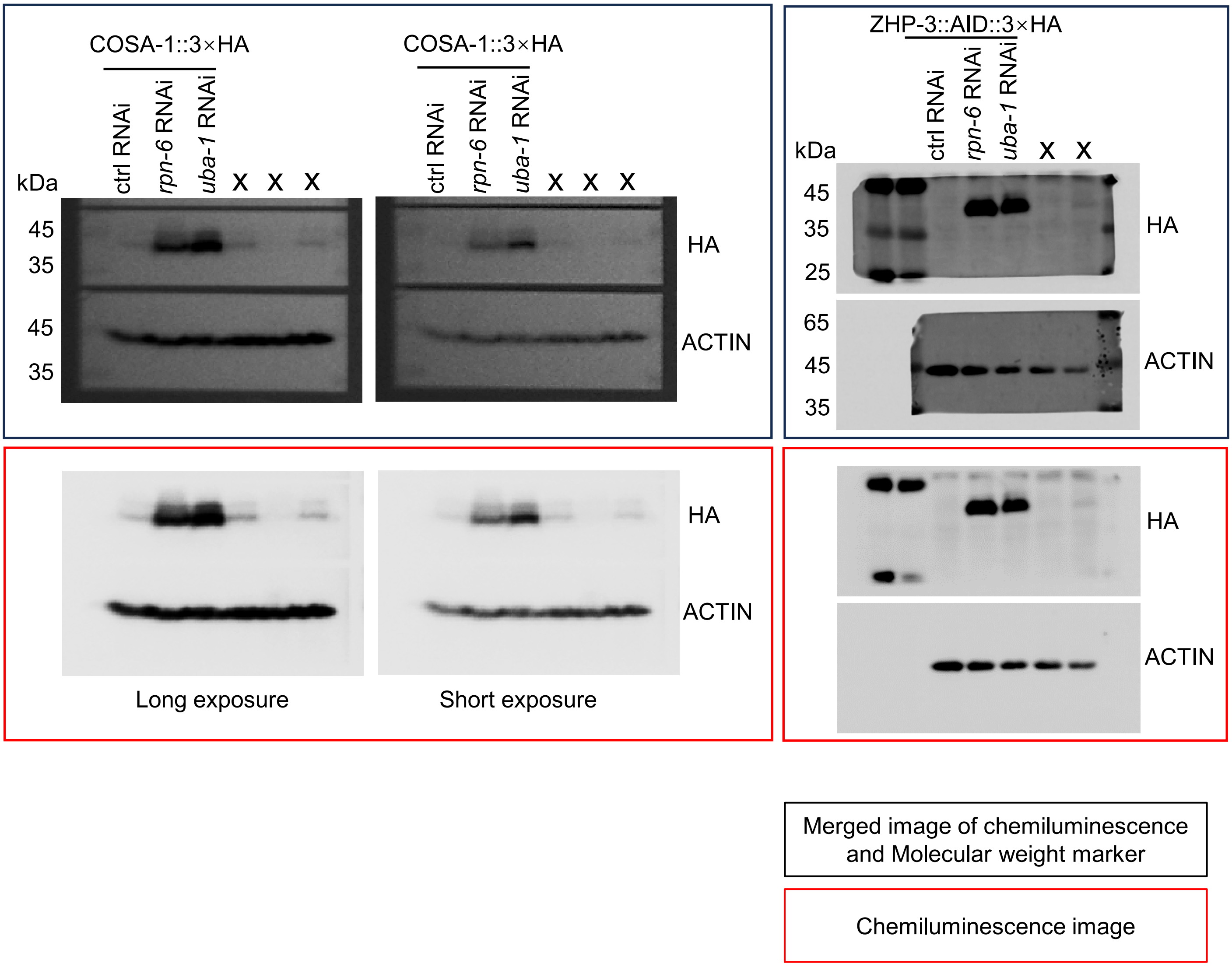
COSA-1::3×HA
ctrl RNAi
rpn-6 RNAi
uba-1 RNAi
X X X
kDa
45
35
45
35
COSA-1::3×HA
ctrl RNAi
rpn-6 RNAi
uba-1 RNAi
X X X
HA
ACTIN
ZHP-3::AID::3×HA
ctrl RNAi
rpn-6 RNAi
uba-1 RNAi
X X
kDa
45
35
25
65
45
35
HA
ACTIN
HA
ACTIN
Long exposure
Short exposure
HA
ACTIN
Merged image of chemiluminescence and Molecular weight marker
Chemiluminescence image

S1C Fig

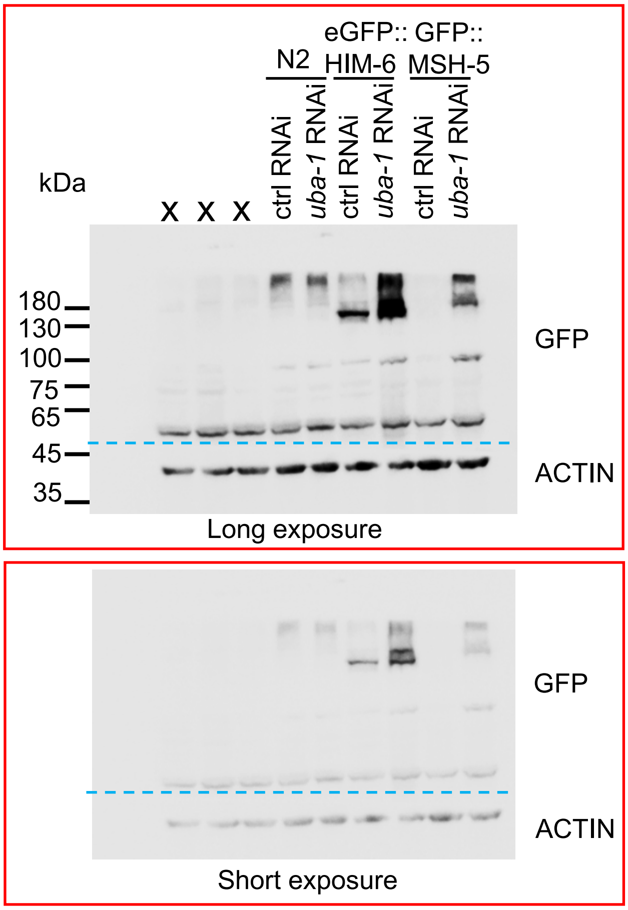

S4A Fig

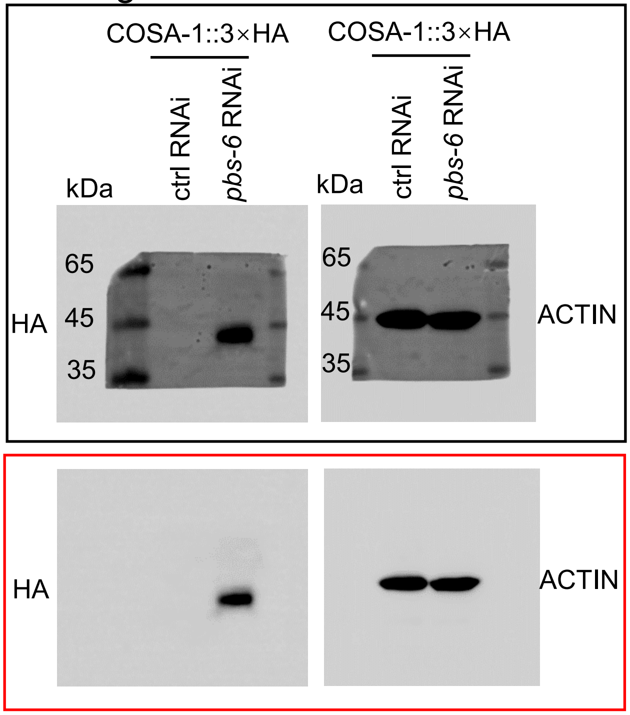

S1D Fig

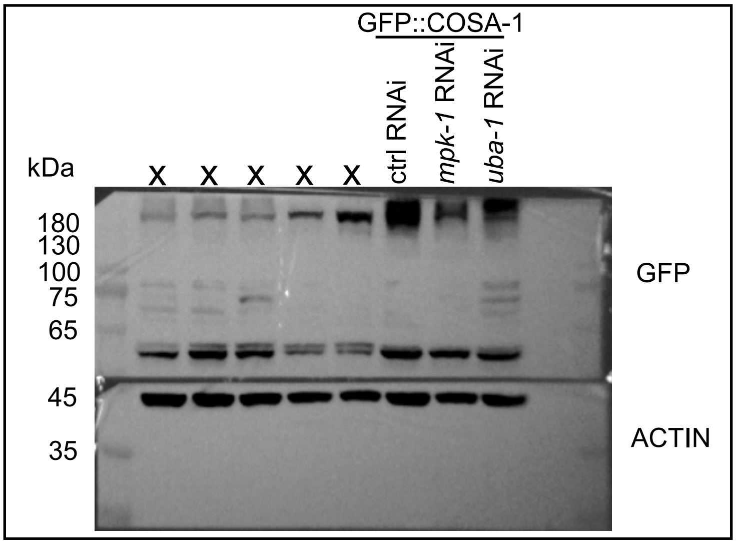

S1E Fig

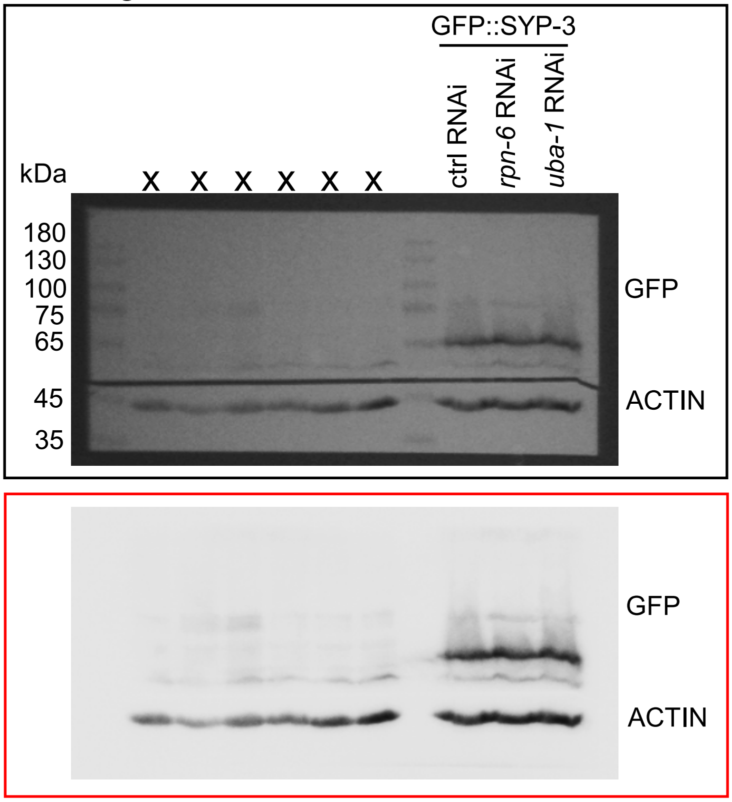

Merged image of chemiluminescence and Molecular weight marker

Chemiluminescence image
