# Supplementary material for: Ubiquitin-proteasome system regulates pro-crossover protein dynamics during meiosis in Caenorhabditis elegans
Source: PLoS Biol. 2026 Jun 16;24(6):e3003868. doi: 10.1371/journal.pbio.3003868 (PMC13293516; doi:10.1371/journal.pbio.3003868)
Supplement: S4 Table — (DOCX) [file pbio.3003868.s014.docx]

**S4 Table. List of strains used in this study.**

| **Strain#** | **Strains** | **Source** |
| --- | --- | --- |
| sYH_0007 | N2 | Caenorhabditis Genetics  Center |
| sYH_0124 | Hawaii (CB4856) | Caenorhabditis Genetics  Center |
| sYH_0100 | *mls12* (CB5584) | Caenorhabditis Genetics  Center |
| sYH_0348 | *cosa-1::3×HA III* | This study |
| sYH_0270 | *cosa-1::mCherry III* | Ezechukwu et al., 2022 |
| sYH_0008 | *eGFP::him-6* *IV* | Caenorhabditis Genetics  Center |
| sYH_0513 | *GFP::cosa-1* *(AV630)[pie-1p::GFP::cosa-1 + unc-119(+)] II.* | Yokoo et al.,  2012 |
| sYH_0059 | *GFP::msh-5* *IV* | Janisiw et al.,  2018 |
| sYH_0740 | *spo-11 deletion/nT1 IV* | This study |
| sYH_0097 | *axIs1914 [syp-3p::GFP::syp-3 3'UTR + unc-119(+)]*(abbreviated as *GFP::syp-3*)*.* | Caenorhabditis Genetics  Center |
| sYH_0087 | *zhp-3::GFP IV* | Balla et al., 2008 |
| sYH_0633 | *zhp-3::AID::3×HA* *I* | Yang et al., 2024 |
| sYH_0099 | *DCL569 (mkcSi13 [sun-1p::rde-1::sun-1 3’UTR + unc-119(+)] II; rde-1(mkc36) V)* | Zou et al., 2019 |
| sYH_0685 | *DCL569; cosa-1::3×HA III* | This study |
| sYH_0708 | *cosa-1::mCherry III; GFP::msh-5 IV* | This study |
| sYH_0389 | *ollas::cosa-1::3×FLAG III* | Yang et al., 2024 |
| sYH_0741 | *ollas::cosa-1::3×FLAG III; spo-11 deletion/nT1 IV* | This study |
| sYH_0510 | *ollas::cosa-1^PD^::3×FLAG III*  *P51D52 of cosa-1 mutated into alanine.* | This study |
| sYH_0133 | *GFP::msh-5 IV; mcherry::H2B* | This study |
| sYH_0847 | *ollas::cosa-1^PD^::3×FLAG III; GFP::msh-5 IV; mcherry::H2B* | This study |
