# Supplementary material for: Ubiquitin-proteasome system regulates pro-crossover protein dynamics during meiosis in Caenorhabditis elegans
Source: PLoS Biol. 2026 Jun 16;24(6):e3003868. doi: 10.1371/journal.pbio.3003868 (PMC13293516; doi:10.1371/journal.pbio.3003868)
Supplement: S5 Table — (DOCX) [file pbio.3003868.s015.docx]

**S5 Table**

**crRNAs, repair templates and genotyping primers used in this study.**

| Editing strains | crRNAs and repair templates | Genotyping primers and  fragment sizes |
| --- | --- | --- |
| *cosa-1::3×HA* | crRNA:5’-cagagatggtagttacgagg-3’;  Repair template-PCR donor with  single stranded overhangs:  5’-gtattccggaatgcagcacctcctcgggaggctcaggatacccatacgacgtcccagactacgcctacccatatgatgtcccggattacgcttacccatacgatgttccagattacgcttaactaccatctctg-3’;  5’-gcgcgaaaaaggtaactgctggccgagttttttctaggccacgcgtggcaattttacaattaattattttttatttatttcagaatgagagtattccggaatgcagcacctcctcgggaggctcaggatacccatacgacgtcccagactacgcctacccatatgatgtcccggattacgcttacccatacgatgttccagattacgcttaactaccatctctgacagcacctctttgtcgccgattccactggtcgcggctcgttcactgcaacaaattattgatttttattgtcatgtaccatattgaatgcat-3’; | 5’-gtgacaatgcttatgtcgaaccat-3’;  5’-gtggtgcaatgagtacgtgac-3’  wt, 300 bp; mutant, 400 bp |
| *OLLAS::cosa-1^PD^::3×FLAG* | crRNA:5’-tcatatgacacgctccccga-3’;  Repair template-PCR donor with  single stranded overhangs:  5’-atccgaatctgaagaaaaatgagccaaaaagcgacaatgagccaccgaaaacgctggtttcaatggaagctgcattttatgaccctagaggtgcgtgtcatatgatttattggacggattgcattgcacaaatggctgttgatattcgag-3’;  5’-acagcaagaaaattgagagaaaactggctgaaaatggaaataattgattttttttttgattttttaatttttcagaccgtaaaaaatccgaatctgaagaaaaatgagccaaaaagcgacaatgagccaccgaaaacgctggtttcaatggaagctgcattttatgaccctagaggtgcgtgtcatatgatttattggacggattgcattgcacaaatggctgttgatattcgaggtgtttatatcggaaattcaatgtttatattgcaaaaattcgtaaatttcaaggtttttaaccttaaaaattgggaaaattaggc-3’ | 5’-cggtgaatcgaacctcattggg-3’;  5’-ctggtttcaatggaacctgca-3’;  mutant, 393 bp;  5’-cggtgaatcgaacctcattggg-3’;  5’-ctggtttcaatggaacctgat-3’  wt, 393 bp; |
| *spo-11 deletion* | crRNA1:  5’-gcacgggtcccgcagcgaat-3’  crRNA2:  5’-agtactgagactgagaagtg-3’  Repair template-blunt end PCR donor:  5’-gtcgattggccagagaggcaaagtgaggagaaacactattggaaatggagagacgcagacactctgcatgtctgcctgcattggaaacgggccattgcggcggagaggacaaatgaatgggaaaagagacaagaaaagaaggaatagtggaaaatacaagaagactacggtagatatagaccatatcagagcttcattatgactatttgaataaagtatttgaaggaaatttaatatagcaaaaatatttcagcgcaacaacatttaatgctagtttaagaaaaaaatgaatactatacataaattttatattctataaaaatcttcttcgactgcgacgaaatgaatggtgcagtcattcgcttcaaaattgcatttaagctgtggtttttcggcagggattgagaaaaaccgagttatatttaagaaatgcattttaaaatattttttgaagaaaaatgtcaatttaagaata | 5’-gtcgattggccagagaggc-3’  5’-ggatggtctggaaacatggag-3’  wt, 2900 bp; mutant, 1058 bp |
|  | tagaaaagaagatcgaacattgagactcgtcggcgccgagtattaaaaatctgacgaaaattacagtactactccggtggggggagaaacaacattaaataaatagaaaacaacacaagttatcttatcttatcacaatatcatcgagtgcataaagctaatggaatggggagatgttttacattgtttagagctcagagtgctcctcctcaatcgttgtttcttcagcggcttctggctctggttcagcctcttcaatatgctgttcggtttcaacttgagcatcttgggaaacatcgagcgattggcgaaggacagcttcgattctatcagcaaaaccaacttgatcttgaagtgagaatccagaacgaagagtggcagtttcgaagagaagcttggcagtagaagcagcggtggtatcttcttcagaagcagtgactctcttgagaagctccttgataactggatggcgtgggttaatctcgaatgtctttttctgagtagcatagaagtcttgagttggatcctttgcc-3’ |  |
