# Supplementary material for: Ubiquitin-proteasome system regulates pro-crossover protein dynamics during meiosis in Caenorhabditis elegans
Source: PLoS Biol. 2026 Jun 16;24(6):e3003868. doi: 10.1371/journal.pbio.3003868 (PMC13293516; doi:10.1371/journal.pbio.3003868)
Supplement: Attachment — Submitted filename: PB-revision-responses to reviewers.pdf [file pbio.3003868.s018.pdf]

We are grateful to the time and constructive feedback provided by the reviewers. We are encouraged by their overall positive assessment that the study is interesting and the data are of high quality. In the revised manuscript we have addressed all the various points that were raised and included substantial new data.

- Highlights include:

1. Three more biological replicates for the DIA proteomics (Fig 1C and 1D) were added to strengthen the proteomics data. The core findings regarding the enrichment of pro-crossover proteins have been robustly reproduced.

2. New data demonstrating *rpn-6* RNAi rescue of *cosa-1*^{PD} have been incorporated (Fig 5C and 5D).

3. New quantification data about the chromosome associated COSA-1 foci upon *uba-1*, *rpn-6*, *pbs-6* and *rpn-6*+*pbs-6* RNAi were added (Fig 3D) to allow for direct comparison regarding the dissociation phenotype.

4. The Discussion has been significantly expanded and included a new reference (PMID: 41650243). We now provide a more comprehensive consideration of the results, specifically elaborating on the role of the UPS in crossover interference. Based on our findings, we have summarized and discussed two potential mechanisms through which the UPS may act to restrict crossover formation.

Below we address all of the specific points raised by the two referees.

Black = Referee comments
Red = **Our reply**

REVIEWS:

Reviewer #1: The manuscript by Zhang et al. investigates how the ubiquitin-proteasome system (UPS) controls the behavior of pro-crossover proteins during C. elegans meiosis. Using a combination of Data-Independent Acquisition proteomics, RNAi screens, and cytological analysis, the authors show that inhibiting the E1 enzyme UBA-1 or the proteasome increases levels of pro-crossover proteins and elevates crossover designation. Interestingly, blocking ubiquitination, but not the proteasome activity, causes pro-crossover proteins to persist on meiotic chromosomes, and their removal requires the CDC-48 segregase complex. Using a hypomorphic allele of cosa-1, the authors further show that partial uba-1 knockdown rescues crossover formation, supporting a model in which the UPS restricts crossover number by tuning pro-crossover protein levels and ensuring their timely dissociation from chromatin.

This study addresses an important question in meiotic regulation, and the identification of pro-crossover factors as UPS targets is very interesting. The experimental approaches are largely appropriate and the data are of high quality. However, there are several concerns regarding the interpretation of the results, the statistical power of the proteomics data, and the lack of evidence demonstrating RNAi efficiency (particularly for the proteasome core). Addressing these points and incorporating the suggested quantifications and explanations would substantially strengthen the manuscript.

Main points:

Figure 1: Statistical Power of Proteomics

The Methods and Figure 7 legend indicate that the DIA proteomics relied on N = 2 biological replicates. N = 2 yields very poor statistical power and likely results in a large False Discovery Rate (FDR) for many proteins. For a study relying on this data to define the mechanism, this is below the standard for the field. Increasing the biological replicate number is highly recommended. If this is not possible, the authors must provide a strong justification in the text (e.g., extreme rarity of samples) and be more transparent about the statistical limitations.

We thank the reviewer for raising this important point regarding statistical power. As suggested, we have repeated the DIA proteomics experiment, now including three biological replicates. While some variations were observed between the original and the new dataset, the core finding that a consistent upregulation of known pro-crossover proteins upon *uba-1* knockdown was robustly reproduced. Key updates from the new analysis include:

MSH-5 was not detected in either control or *uba-1* RNAi samples in this replicate set; COSA-1, which was not captured previously, was now reliably detected; ZHP-3 was specifically detected in the *uba-1* RNAi condition, resulting in a high fold-change value.

All relevant figures (Figs 1, 7 and S1) and the corresponding results section and S1 Table have been updated to reflect this new, more statistically robust dataset. We believe these revisions adequately address the concern regarding sample size and improve the reliability of our proteomic conclusions.

Figure 2B: If altering levels of pro-CO factors by uba-1 RNAi induces double crossovers as shown in Figure 6, this should result in ring-like bivalents. Have the authors observed such structures?

We agree with the reviewer's insightful point. The induction of multiple crossovers by *uba-1* RNAi is indeed theoretically expected to result in ring-like bivalents. While such structures have been primarily documented in *C. elegans* studies using fused chromosome (PMID: 24107990 and 32886661), under our experimental conditions with *uba-1* RNAi, we have

indeed observed configurations consistent with ring-like bivalents. Representative images of these structures have now been included as supplemental panels in S7 Fig for reference. Please check lines 528-530 for the description of this observation.

SYP-1 appears abnormal in RNAi-treated animals, as it is continuous between two chromosomes in rpn-6 RNAi-treated oocytes, and there more additional smaller SYP-1 foci in uba-1 RNAi-treated oocytes. Can the authors comment about this?

We thank the reviewer for this careful observation. Indeed, SYP-1 localization appears altered under these RNAi conditions. While our quantification did not indicate an increase in overall SYP-1 abundance, the ubiquitin-proteasome system (UPS) is known to regulate the dynamic turnover of synaptonemal complex components, including SYP-1. Furthermore, as our data show that knockdown of UPS components leads to an increase in crossover formation, the resulting changes in chromosome organization and recombination intermediate processing could secondarily affect the pattern of SYP-1 assembly and distribution along the chromosomes. Thus, the abnormal SYP-1 morphology is consistent with the broader disruption of meiotic regulation observed upon UPS impairment. We have made a brief comment about this at lines 242-247.

Figure 2D: The authors observe MSH-5 foci that lack COSA-1 and interpret this as a failure of the UPS to "dismantle recombination intermediates". Could it be that the persistent MSH-5 foci represent excess protein aggregates that cannot be degraded, rather than bona fide recombination intermediates? Perhaps showing additional recombination marker (e.g. RMH-1) would resolve this issue.

We thank the reviewer for raising this important point regarding the interpretation of persistent MSH-5 foci. The reviewer’s suggestion to examine an additional recombination marker such as RMH-1 is well-reasoned. After careful consideration, we have concluded that this particular experiment may not definitively resolve the issue and the interpretation of the result would remain ambiguous. If RMH-1 colocalizes with the persistent MSH-5 foci, it could support their identity as recombination intermediates; however, we cannot rule out the possibility that RMH-1 itself, like other recombination proteins, might associate non-specifically with protein aggregates under conditions of UPS impairment. Conversely, a lack of RMH-1 colocalization would not be conclusive evidence against the foci being genuine intermediates, as the recruitment of RMH-1 to all types of recombination intermediates is not fully established.

Therefore, we have revised the text to more cautiously frame our interpretation. We now explicitly state that the persistent MSH-5 foci could represent either unprocessed recombination intermediates or non-degradable protein aggregates (lines 275-277).

In addition, we have substantially updated the data in this section as suggested by another reviewer. Following their recommendation, we quantified the frequency of MSH-5-only foci and COSA-1-only foci from our existing datasets. We found that upon UPS knockdown, COSA-1-only foci were also present, though at a lower frequency compared to MSH-5-only foci. Based on this quantification, we have moderated the previous conclusion. It is now phrased as: 'These results suggest that the UPS may be involved in clearing pro-crossover proteins that have already accumulated at non-crossover sites under physiological conditions,' rather than the stronger previous statement, 'these results implicate the UPS in dismantling recombination intermediates that are marked by MSH-5 but fail to accumulate COSA-1.' (lines 273-279)

Figure 3D-E: Missing controls for *pbs-6* RNAi

A key claim is that ubiquitination (*uba-1*), but not the proteasome core (*pbs-6*), is required for the dissociation of pro-crossover proteins (Figure 3D). However, the authors do not provide evidence that the *pbs-6* RNAi was effective. Without a Western blot or IF demonstrating that *pbs-6* RNAi caused a global accumulation of COSA-1/ZHP-3 similar to *rpn-6* RNAi, the result is uninterpretable.

We thank the reviewer for raising this important point. As suggested, we performed the Western blot and IF analyses, which confirm that *pbs-6* RNAi does indeed cause a global accumulation of COSA-1 (S4A to S4C Fig), similar to the effect observed with *rpn-6* RNAi. This new result validates the effectiveness of our *pbs-6* knockdown. The corresponding description have been added to the manuscript (lines 339-342).

After the paragraph ending at line 315 before leading up to the next section, it would be helpful to conclude the results with potential explanations. I suggest the authors to lay out both possibilities up front: (1) the involvement of CDC-48 and (2) the necessity of ubiquitination for processing DNA substrates (e.g. resolution).

We thank the reviewer for this constructive suggestion. Accordingly, we have revised the paragraph to conclude with a discussion of potential mechanistic explanations, as recommended. We have incorporated the possibility that certain segregase may be involved in disassembling polyubiquitinated pro-crossover complexes (lines 357-364). To avoid repetition, we have removed the now-redundant introductory sentences from the next section.

Regarding the second possibility that ubiquitination is necessary for processing DNA substrates (e.g., for resolution), our experimental observations do not appear to support this model. Specifically, in our study, the efficient processing of DNA substrates into crossover products was not impaired upon *uba-1* knockdown; rather, crossovers were produced in excess. This outcome suggests that the resolution step itself can proceed effectively even when ubiquitination is compromised, arguing against an essential role for

ubiquitination in this particular stage of substrate processing. Therefore, we have not laid out this possibility.

Figure 4: Penetrance of *cdc-48* RNAi knockdown

The text states that CDC-48 is "mediated" and "responsible for" dissociation. However, the data in Fig 4C show that only a small fraction (~15-20%) of -1 oocytes retain foci upon cdc-48 knockdown. This low penetrance does not support the claim that CDC-48 is the primary segregase for this process. I suggest that the authors acknowledge this issue and compare the *cdc-48* RNAi phenotype with *uba-1* RNAi to evaluate to what extent they phenocopy each other.

We thank the reviewer for raising this important point. We indeed noticed this phenotypic difference between the *cdc-48*/*npl-4* and *uba-1* knockdowns. We reasoned that this likely reflects limitations in detection sensitivity. First, following *uba-1* RNAi, the protein levels of COSA-1 and other pro-crossover factors are elevated, making any delay in their dissociation easier to detect. Second, when we performed *npl-4* RNAi in a different experimental setup using transgenic worms expressing GFP::COSA-1 or ZHP-3::GFP, the penetrance of the delayed dissociation phenotype (70–90%) approached that observed in *uba-1* RNAi (100%). The remaining difference may suggest the existence of another redundant segregase or reflect incomplete RNAi knockdown of *npl-4*. These results indicate that the CDC-48/NPL-4 complex is indeed required for the efficient removal of pro-CO factors.

As the reviewer may have noted, we primarily used *npl-4* RNAi rather than *cdc-48* RNAi to disrupt the function of the underlying CDC-48/NPL-4 segregase complex. This choice is based on the following observations: First, knockdown of either *cdc-48* or *npl-4* resulted in the retention of COSA-1 and ZHP-3 on diakinesis chromosomes, confirming that CDC-48 and NPL-4 function within the same pathway. Second, regarding the delayed dissociation phenotype, *npl-4* RNAi produced a more pronounced effect than *cdc-48* RNAi. We attribute this difference to a comparatively weaker knockdown efficiency of *cdc-48* by RNAi.

We have incorporated the above-discussed points into the revised manuscript (lines 375-387), toned down phrases such as ‘responsible for’ to more accurate descriptions like ‘is required for the efficient dissociation of pro-CO factors’ (lines 394-395, 419-422), and also admit the possibility of the existence of another segregase (lines 419-422).

Minor points:

Figure 1A: The volcano plot highlights BRD-1 alongside known pro-crossover factors. However, the text does not explain its relevance. Please briefly mention BRD-1's known or suspected connection to meiosis to justify its inclusion in the figure.

We thank the reviewer for this helpful suggestion. To clarify the relevance of BRD-1 in our analysis, we have added a brief explanation in the text (lines 139-141). BRD-1 forms a heterodimer with BRC-1, and this complex is known to accumulate at crossover-designated sites, colocalizing with pro-CO factors, implicating it in the regulation of crossover formation (Janisiw et al., 2018; PMID: 30383754). In our updated DIA proteomics dataset, BRC-1 was also identified as differentially regulated. Therefore, we have included and highlighted both BRD-1 and its partner BRC-1 alongside known pro-crossover factors in Fig 1C for a more comprehensive representation.

Figure 1B: Since the experiment tested hundreds of proteins, it would be more appropriate to report both FDR and p-values for the highlighted hits.

Thank you for the suggestion. We have added a table in Fig 1D to report the FDR and P values for the highlighted hits.

Figure 1D-F: Please add small, high-magnification insets of representative nuclei for different meiotic stages for the rpn-6 and uba-1 RNAi conditions to clearer show the localization of mis-regulated COSA-1 and ZHP-3.

We have added images for TZ through LP in S2A Fig. DP and DK have not been included as these two stages have already been shown in Fig 3 and Fig 4.

Figure 3: Please add a quantification panel scoring the percentage of -1/-2/-3 oocytes with chromosome-associated foci across control, *uba-1*, *rpn-6*, and *pbs-6* RNAi to allow for direct comparison.

Thank you for the suggestion. We have now added quantification panel in Fig 3D (for COSA-1::3HA with control, *uba-1*, *rpn-6*, *pbs-6* and *rpn-6*+*pbs-6* RNAi) and S4D Fig (for GFP::COSA-1 with ctrl, *pbs-6* and *uba-1* RNAi) for direct compare the effects of ubiquitination and proteasomal degradation.

Line 55: Please define the ZMM proteins. It appears that this term is used distinctively from pro-crossover factors. If ZMM proteins are broadly defined as proteins that stabilize crossover-specific recombination intermediates, CDK-2 and COSA-1/CNTD1 can certainly be classified as the ZMM proteins.

Thank you for raising this point regarding the definition of ‘ZMM proteins’. We agree that the term can be ambiguous especially when used together with pro-crossover factors. In response, we have replaced the broad term ‘ZMM proteins’ with the more specific description: ‘the Zip3 family of proteins and the conserved MutSγ complex’, and the sentence now reads ‘This group of factors, comprising the Zip3 family of proteins, the

conserved MutSγ complex, COSA-1/CNTD1 and the MutLγ complex, dynamically associate with the chromosomes in a meiosis stage-dependent manner.’ (lines 66-69).

Line 64: Reference 19 is not published in Nature Cell Biology (https://doi.org/10.1038/s41556-025-01688-9)

We thank the reviewer for the careful check of the references. We have double-checked the citation and verified that the article is indeed published in *Nature Cell Biology*. The DOI provided (https://doi.org/10.1038/s41556-025-01688-9) resolves to the *Nature Cell Biology* website.

Line 70: It looks like the abbreviation for non-crossover (NCO) is not used throughout the manuscript. I suggest to remove it.

Thank you. We have removed it in the revised manuscript.

Reviewer #2: In their manuscript "Ubiquitin-proteasome-mediated regulation of pro-crossover protein dynamics contributes to crossover patterning during C. elegans meiosis", Drs. Zhang, Liang, Li. and colleagues explore how meiotic recombination is affected by defective degradation via the ubiquitin-proteasome system. Analyzing consequences of depletion of E1(Uba-1)-mediated ubiquitin activation and of proteasome RP and CP components, they show that RNAi-mediated depletion of these essential components has several intriguing effects: First, crossover-associated proteins such as COSA-1 and MSH-5 form foci in excess of those normally observed in C. elegans, even though other meiotic chromosome components are not affected in the same way. Second, rather than disappearing at the end of pachynema, foci tend to persist into later stages of meiosis, diplonema or even diakinesis. Third, COSA-1 abundance is increased in the nucleoplasm of cells throughout meiosis. Fourth, instead of forming one crossover per homologue pair, both cytological and importantly also genetic analysis indicates that additional crossovers are formed frequently. The work further explores whether persistence of CO-proteins on meiotic chromosomes is related to a failure to displace them via Cdc48.
Overall, this is an intriguing piece of research. It gives rise to the idea that simply the failure to degrade pro-crossover factors is sufficient to keep them in place along chromosomes, increase their localization to additional sites and, most surprisingly, result in the formation of additional crossovers, essentially overriding mechanisms that limit the frequency of crossovers. This is quite a surprising finding, as it suggests a critical role of the UPS system in limiting crossovers, above and beyond of just targeting components of the recombination machinery. Moreover, limiting the availability of CO proteins might be a key determinant for CO numbers.
The data quality and analyses are quite impressive. My suggestions for revisions are mostly textual, even though some additional analysis of existing data may also be required. Overall, the Discussion is rather superficial and lacks detailed consideration of the rather

complicated set of results.

Major points:
L47: "The mechanism underlying crossover interference remains elusive." - This statement really understates the status of current research. There are various models out there, among them the coarsening that is brough up later in the manuscript, but also the stress hypothesis, involvement of the SC or not, role of topo2 and the UPS system. Rather than elaborating too much on class II crossovers which, as the authors state, likely do not exist in their experimental system, they should at least paraphrase the current status of insights.

We thank the reviewer for raising this important point. We have now briefly introduced factors known to regulate crossover interference, outlined the two prevailing models proposed for its underlying mechanism, and focused in greater detail on the coarsening model (lines 47-60).

L48: DSBs have not been introduced.

Thank you for this correction. We have now introduced and defined ‘DSBs’ upon its first mention in the text (lines 39–41).

L125: The GO annotation is so redundant that it is simply not informative. There is "cell cycle", "cell cycle regulation", "mitotic cell cycle"-this all seems to refer to the same thing. Can you do Revigo or other analysis that reduces redundancies? I understand that you are not using the data, but it will make things more useful for the general reader.

We thank the reviewer for this suggestion. We have eliminated redundancy and retained a more informative set of the top 10 representative GO terms. The revised GO term annotation is now presented in S1B Fig.

L130: REPLACE "was not identified" with "detected"

In the revised manuscript, as COSA-1 was detected in the DIA data, so we deleted the original sentence, and therefore this concern is no longer relevant.

Fig. 1D: It needs to be mentioned in the text that this staining is initially nucleoplasmic and later transitions to foci along chromosomes. I suggest showing an excerpt of the region where uba-1 and rpn-6 exhibit the transition from excessive nucleoplasmic COSA-1 or ZHP-3 staining to foci. (Either here or in Fig. 2A where this is indicated by a (barely visible) dashed white line.)

We thank the reviewer for this helpful suggestion. To better illustrate the dynamics of COSA-1 and ZHP-3 localization, we have now added high-magnification insets of

representative nuclei from the TZ to LP stages under *rpn-6* and *uba-1* RNAi conditions in S2A Fig. These insets clearly show the transition from initially diffuse nucleoplasmic staining to distinct chromosomal foci. We also explicitly described this transition in the text (lines 214-221). Additionally, the dashed white line in Fig 2A has been bolded for improved visibility.

L142/Fig. 1E: Unclear whether this quantitation was done for entire germ line. Also, please provide the number of germlines analyzed.

Thank you for raising these points. The quantification of the immunofluorescence intensity was performed from the distal tip to the end of the pachytene region of the gonad, as described in our Statistics section of Materials and Methods. To improve clarity, we have now explicitly stated this in the Fig 1 legend, and have also added the number of germlines analyzed there (lines 162-163).

L186: I would show the spo-11 result in the main figure. Also, please state clearly whether there are any foci detected in spo-11 or only staining in the nucleoplasm

We thank the reviewer for this suggestion. As recommended, we have now included the data of UPS knockdown in the *spo-11* background in the main figure (Fig 1J and 1K). Regarding foci formation, in agreement with previous studies (PMID: 22464324, 41124211), 1 to 2 COSA-1 foci were observed within the oocyte nuclei at late pachytene in the *spo-11* mutant (S2B and S2C Fig). Following *rpn-6* RNAi, the number of foci remained largely unchanged, but their intensity was significantly increased, presumably due to elevated COSA-1 protein abundance (S2B and S2C Fig). Interestingly, *uba-1* RNAi led to a marked increase in the number of COSA-1 foci (S2B and S2C Fig), revealing a fundamental divergence in how ubiquitination and the proteasome pathway regulate COSA-1 localization within a genetic background incapable of crossover formation (lines 228-235).

L197: The section on MPK-1 needs a conclusion to be useful, e.g., increased COSA-1 abundance is not an indirect consequence of progression defect. It is not clear to this reviewer whether there is a progression defect in uba-1 or rpn-6. Apparently not, but why then introduce mpk-1?

We initially aimed to identify a genetic context in which depletion of UPS components would not elevate pro-crossover proteins such as COSA-1. While the *mpk-1* mutant provided such a background, the mechanistic basis for this observation remains unclear and the findings do not directly contribute to the core narrative of our study. Therefore, in light of the reviewer's suggestion, and to maintain a clear and focused presentation, we have removed this data from the revised manuscript.

L205: "COSA-1 accumulation" is very unspecific- do you mean additional foci and/or more intense foci and/or nucleoplasmic staining?

We thank the reviewer for pointing it out. We have clarified in the revised manuscript (lines 217-221) that we specifically refer to 'COSA-1 foci formation' rather than the more general 'accumulation' term.

L224//Fig. 2A: The spatial patterning of nuclei in straight lines in the two mutant germlines, but not the WT, looks unusual. It should be clearly stated whether this is part of the mutant phenotype.

We thank the reviewer for this careful observation. The apparent alteration in spatial patterning is likely attributable to a global increase in COSA-1::mCherry signal, both cytoplasmic and focal, resulting from elevated protein levels upon UPS impairment (*rpn-6* or *uba-1* RNAi). Importantly, despite this overall signal enhancement and the presence of brighter, larger COSA-1 foci, their spatiotemporal regulation remained intact: foci were strictly confined to the normal late pachytene region (indicated by the dotted line in Fig 2A). This indicates that the timing of crossover designation is tightly controlled and resilient to perturbations in protein turnover.

Fig. 2B: While the SC/axis differentiation appear to be intact in the UPS mutants, the morphology of diplotene nuclei looks quite different (as would be expected if there are additional crossovers). Can you quantitate the aberrant morphologies?

We thank the reviewer for this insightful comment. We agree that diplotene chromosome morphology appears altered upon UPS knockdown, such as shortened or disorganized axes, as indicated by HTP-1 staining (lines 242-247). This early abnormality likely underlies the more severe condensation defects we observe at later stages. However, directly quantifying these subtle morphological changes at the decondensed diplotene stage is inherently challenging due to the lack of clear, standardized benchmarks.

Fig. 2C/L242/L247: Please quantitate how many MSH-5 foci without COSA-1 and vice versa. This is important for a mechanistic understanding: Is MSH-5 persisting and COSA-1 following suit or are both persisting coordinately. This should also be addressed in the Discussion.

We thank the reviewer for this important suggestion. We have now quantified the co-localization of MSH-5 and COSA-1 foci in the relevant genetic backgrounds. Our analysis revealed the presence of both MSH-5-only and COSA-1-only foci, with MSH-5-only foci being more frequent (S3D and S3E Fig). This result supports a model in which the UPS is involved in clearing pro-crossover proteins that have been loaded at recombination sites but are destined for non-crossover outcomes. The higher frequency of MSH-5-only foci is

consistent with its earlier accumulation at recombination intermediates and its initial formation of more foci than the later-acting COSA-1.

We have incorporated the description of these quantitative results in the revised Results section (lines 273-281), and have addressed their mechanistic implication in the Discussion. Specifically, we discuss this finding as supporting evidence that the UPS reinforces crossover interference by regulating the chromosomal localization of pro-crossover factors (lines 728-732).

Discussion: I understand that in C.e., it is widely considered as a loss of CO interference if there are 2COs or 3COs on a given chromosome. But in the current case, it seems a reasonable alternative that a defective UPS simply results in more COs. Whether these crossovers still interfere with each other would depend on coefficient-of-coincidence analysis, which is difficult with this small data set. The authors should address this point at the very least in the Discussion.

We thank the reviewer for this suggestion. We agree that the observed increase in chromosomes with multiple COs upon UPS knockdown could reflect either a true loss of interference or a general increase in the number of COs, without necessarily abolishing interference. Distinguishing between these models would require a formal analysis of interference strength (e.g., coefficient of coincidence), which is not statistically robust with our current dataset. We have therefore addressed this alternative interpretation in the Discussion (lines 648–661) and clarified that, while the data are consistent with weakened interference, we cannot rule out a scenario in which CO number is elevated while interference remains intact.

L273/Fig. 3: The argument that the E1 ligase is more critical than the proteasome is the least convincing part of the paper. Isn't it possible that the E1 ligase is more limiting, and hence the knockdown effects are more dramatic and/or lasting into later cellular stages compared to the proteasome subunit knockdown which still allows many fully functional proteasome particles to be assembled.

We thank the reviewer for raising this important point, which highlights an alternative interpretation of our data. Our experiments revealed that simultaneously knocking down both *rpn-6* and *pbs-6* did not enhance the pro-CO protein retention phenotype as compared with rpn-6 or pbs-6 single RNAi, and these treatments were consistently less effective than *uba-1* knockdown. Moreover, similar to *uba-1* RNAi, both *rpn-6* and *pbs-6* RNAi led to an elevation of COSA-1 protein. We note that the difference in the delayed dissociation of COSA-1 protein following *rpn-6* and *pbs-6* RNAi, compared with *uba-1* RNAi (11% vs 100%, Fig 3D), is far greater than the difference observed at the level of COSA-1 protein elevation (fold change 8.7 vs 10.2, Fig 1G). This indirectly suggests distinct functional roles of E1/ubiquitination and the proteasome in the dissociation process. While this suggests the proteasome may not be the direct effector of dissociation in this context, we acknowledge

that our experiments do not definitively rule out the alternative explanation reviewer proposed.

To address this concern and to present our conclusions more cautiously, we have made the following modifications to the manuscript:

1. We have changed the title of the corresponding result section from ‘E1-mediated ubiquitination, but not proteasomal degradation, is required...’ to the more cautious and accurate wording: ‘E1-mediated ubiquitination is required for the dissociation of pro-crossover proteins from diakinesis chromosomes.’ This removes the direct negation of proteasome involvement (lines 289-290).

2. Throughout the results, we now describe the findings with greater precision, stating that ‘knockdown of E1, but not the proteasome subunit, leads to...’. In concluding statements, we have introduced cautious language such as ‘probably’ or ‘suggests’, thereby avoiding an overinterpretation of the functional distinction. For example: ’The above data indicate that ubiquitination, but probably not proteasomal degradation, is responsible for the dissociation.’ (lines 357-359)

3. We have incorporated the reviewer's alternative interpretation in the Discussion section (lines 769-772).

We believe these revisions provide a more balanced and accurate representation of our findings while fully acknowledging the current experimental limitations.

L288: undetectable and completely absent are the same thing.

Thank you. We have revised the sentence and it now reads ‘During normal meiotic progression to diakinesis, pro-CO proteins gradually dissociate from CO sites, becoming undetectable in the majority of -3 and -2, and all -1 diakinesis oocytes.’ (lines 324-326)

L293: So not all foci disappear? How many remain, in how many diakinesis oocytes?

As quantified in the revised Fig 3D, following *rpn-6* RNAi, only 11% (2 out of 18) -1 diakinesis nuclei exhibited COSA-1 foci associated with chromosomes, with 2 to 3 foci per nucleus, while the vast majority of nuclei (16 out of 18.89%) showed no chromosomal COSA-1 foci. This phenotype contrasts sharply with the effect of *uba-1* RNAi, under which every nucleus examined (19 out of 19, 100%) contained COSA-1 foci localized to all chromosomes, as shown in Fig 3B.

L314-L332: I understand that the authors want to address the point whether there is a pathway of polyubiquitination>displacement by Cdc48>degradation by proteasome, but the analysis is not very rigorous, and the rational for double mutants should be better explained, to support or exclude this scenario. If this scenario was the case, shouldn't there

be just nucleoplasmic foci in the proteasome mutant, rather than exhibiting a longer persistence on chromosomes?

We thank the reviewer for this insightful comment.

1. Rationale for the double RNAi analysis:

As the reviewer pointed out, we indeed would like to propose a sequential pathway: protein polyubiquitination > recognition and displacement from chromosomes by the Cdc-48/NPL-4 complex > final degradation by the proteasome. The single *rpn-6* RNAi phenotype, where COSA-1 foci are displaced from chromosomes (predominantly non-chromosomal foci), served as a starting point. It showed that displacement can occur even when degradation is blocked. We then performed *rpn-6* + *npl-4* double RNAi to test if the displacement step is dependent on CDC-48/NPL-4. If our hypothesis is correct, we would expect the phenotype to revert from the *rpn-6* RNAi phenotype (non-chromosomal foci) to resemble the *uba-1* or *npl-4* RNAi phenotype (foci retained on chromosomes). We have added this rationale to the revised manuscript (lines 409-417).

2. Regarding the proteasome RNAi phenotype:

Consistent with the reviewer's expectation, we indeed observed that upon proteasome subunit knockdown, a majority of the foci were nucleoplasmic (only in 2 out 18 nuclei, chromosomal COSA-1 foci were detected, with 2 to 3 foci per nucleus), which contrasts with the pronounced chromosomal persistence seen in *uba-1* RNAi (Fig 3D). This supports the idea that the CDC-48/NPL-4-mediated displacement can proceed efficiently even when proteasomal degradation is impaired.

The prolonged chromosomal signal observed under proteasome subunit RNAi during diplotene is likely a consequence of the overall increased protein abundance (due to stabilization), making the residual chromosomal-associated signal more detectable than wild type. Importantly, these foci were still efficiently dissociated from chromosomes by -1 diakinesis in the proteasome knockdown, a key phenotypic difference from the *uba-1* RNAi condition where dissociation fails (lines 330-332).

In summary, the genetic and cytological data suggest that while the proteasome is required for the ultimate turnover of these proteins, the critical step for regulating their timely removal from meiotic chromosomes is the upstream, UBA-1-dependent (likely ubiquitin-mediated) process that involves Cdc48/NPL-4. We have revised the relevant section in the manuscript (lines 357-359, 409-419, 769-772) to present this interpretation more clearly.

L353: This section is confusing: Fig. 3B shows that rpn-6 results in persistence of ZHP-3 and COSA-1, but the statement in the text claims that it doesn't impair the dissociation of these factors. If you mean that it retains pro-CO factors during diplotene, but remains functional for dissociation during diakinesis, this needs to be said clearly. Also, the reader needs to see the rpn-6 single mutant (again) in this figure, and the entire germline to get a

sense whether the increased levels of COSA-1 in the nucleoplasm are part of the phenotype in the double mutant.

We thank the reviewer for this comment, which helps us clarify a key point. Our data show that *rpn-6* RNAi results in increased abundance and persistence of ZHP-3 and COSA-1 at diplotene, but these factors are still efficiently dissociated from chromosomes by -1 diakinesis oocytes. Therefore, we concluded that the final dissociation of pro-CO proteins does not appear to be dependent on proteasome function, as it still occurs, albeit later, despite high protein levels. We have clarified this point in the main text to avoid ambiguity (lines 328-333).

As suggested, we have added the *rpn-6* RNAi data as a control in Fig 3F and included images of the entire germline in S4E Fig to allow a clearer assessment of the nucleoplasmic COSA-1 level in the double mutant.

L368: I couldn't find the cosa-1-PD allele in your NAR 2024 paper - is it mentioned under a different name? Also, the rationale/approach for identifying effects of these two amino acids needs to be explained, and how these aa are mutated (deleted, replaced by Ala?).

We apologize for the lack of clarity. The designation 'PD' refers to the two amino acids, P51 and D52, in COSA-1. These residues are mutated in the previously published *cosa-1-6A* allele but remain intact in the *cosa-1-4A* allele (Yang et al., 2024). Since *cosa-1-6A* exhibits more severe meiotic defects than *cosa-1-4A*, we hypothesized that P51 and D52 might be functionally important. To test this, we generated a new allele specifically substituting both residues with alanine (P51A, D52A). This mutant is referred to as *cosa-1^{PD}* in the current study. We have clarified these points in the revised manuscript (lines 432-436 and Fig 5A).

Fig. 5I/L393: Please add the rpn-6 and uba-1 single mutants to the panel so the reader can compare the double and single mutants side-by-side

Thank you. We have added quantification for *rpn-6* and *uba-1* RNAi in the wild type background to the panel (Fig 5I).

L401-405: The rescue of the binding-impaired COSA-1 by reducing the UPS machinery is quite impressive, but the section is difficult to follow, and the authors need to do a better job in explaining their rational and findings here. Either decreased ubiquitination or decreased displacement via Cdc48 rescue, but is it unclear why rpn-6 is only shown for COSA-1 foci, but not for DAPI staining bodies.

We thank the reviewer for this positive feedback and for the suggestion to improve the clarity of this section.

To clarify, as noted in the earlier dissociation experiments (Fig 4), n*pl-4* RNAi produced a more penetrant delay in pro-CO protein disassembly than *cdc-48* RNAi. Therefore, for the subsequent functional rescue experiments, we used *npl-4* RNAi as a representative means to inhibit the CDC-48/NPL-4 segregase complex.

As suggested, we have performed *rpn-6* partial RNAi and analyzed diakinesis oocytes for bivalent formation. Consistent with the results from *uba-1* and *npl-4* RNAi, *rpn-6* RNAi also significantly rescued the crossover defects in *cosa-1*^{PD} mutants. These new data are now included in Fig 5C and 5D, demonstrating that knockdown of the proteasome (RPN-6), like that of ubiquitination (UBA-1) or the segregase (NPL-4), can restore bivalent formation.

Although we showed that *npl-4* RNAi partially rescued bivalent formation, it failed to restore COSA-1^{PD} focus formation (S6D Fig), suggesting that the UPS may have a broader functional scope than the CDC-48/NPL-4 complex. Considering that *npl-4* RNAi does not increase protein abundance but delays the disassembly of pro-crossover factors, we propose that by inhibiting the timely disassembly of pro-CO complexes, *npl-4* RNAi may prolong the chromosomal residency of the functionally compromised COSA-1 ^{PD} protein. This extended window may allow more opportunity to complete crossover designation.

We have thoroughly revised the Results section (lines 472-474, 486-492) and the Discussion (lines 751-760) to incorporate these new data and present this logical framework more clearly.

L463: "Consistent with its defect in crossover formation, cosa-1PD exhibited a reduced recombination rate" - I'm not sure what this sentence is trying to say. Crossover formation and recombination rate in this context are the same thing, so the statement sounds rather tautological. Maybe you mean: As expected based on effects on COSA-1 focus formation and viability/HIM effects, crossover frequencies are reduced?

We agree with the reviewer and have revised the sentence to clarify the logic. It now reads 'Consistent with the presence of univalent at Diakinesis and the HIM (High Incidence of Male progeny) phenotype, *cosa-1*^{PD} exhibited a reduced recombination rate.' (lines 516-518).

L446: I do not see any nuclei with >9 COSA-1 foci in cosa-1PD in Fig. 5I, so the argument makes no sense

We apologize for the lack of clarity in our original description. We observed that in the *cosa-1*^{PD} background, treatment with *rpn-6* or *uba-1* RNAi led to a subset of nuclei containing more than 9 COSA-1 foci. In contrast, the same RNAi treatments in the wild-type background did not result in nuclei with >9 COSA-1 foci. We have modified original description to make it clear (lines 526-528).

L450: "Analysis of double and triple crossover positioning revealed no consistent spacing preference, indicating a fundamental breakdown of crossover spacing constraints following UBA-1 depletion (Fig 6B)." - The fact that only 10 events were analyed should be added as a caveat here. Also, as mentioned before, it is unclear to this reader whether we are looking at a breakdown of CO spacing or simply additional events that mature into COs. This alternative explanation should be discussed.

We thank the reviewer for raising these two important points regarding the interpretation of our double and triple crossover data. Regarding the sample size, we fully agree with the reviewer that the relatively low number of analyzed events is an important caveat. We have now explicitly stated this limitation in the revised Results section where this data is presented (lines 536-539). We have acknowledged that our current sample size limits a definitive statistical analysis of interference strength via coefficient-of-coincidence calculations in the discussion (lines 648-652).

Regarding the alternative explanation, as having been raised earlier by the reviewer, we have expanded our discussion of this alternative interpretation. (lines 655-658). We now explicitly state that the observed loss of spacing preference could be interpreted in two ways: 1) as a failure of the mechanistic process that normally enforces interference, or 2) as a consequence of an increased number of designated intermediates maturing into COs. We discuss the implications of both scenarios for understanding the role of ubiquitination in regulating crossover patterning (lines 648-661). We believe these revisions directly address the reviewer's concerns and provide a more balanced and thorough interpretation of these data.

L482: the crossover formation defect was EXAGGERTED [REPLACE deteriorated] as SUGGESTED [revealed] by the increased incidence of univalentS [INSERT]

Thank you. We have modified the sentence as suggested (lines 570-571).

L564: UPS's DUAL [REPLACE bidirectional] functionality

We have replaced ‘bidirectional’ with ‘dual’ (line 634).

L572: The authors should provide a better rational for the MPK-1 experiment or maybe remove it.

We agree that the MPK-1 experiment, while yielding an interesting observation that the depletion of *uba-1* or *rpn-6* no longer affected pro-CO proteins in the *mpk-1* mutant background, does not strengthen our core message and may detract from it. To maintain clarity and focus on our central conclusions, we have removed this data.

L602: Coarsening was never explained in the Introduction and the relevant references need to be provided (e.g. Morgan 2021 and others).

We have introduced and explained coarsening in the revised manuscript and the relevant references have been added (lines 47-60).

L604: Are these additional crossovers really ectopic? Isn't the results simply that some additional DSBs mature into COs - they aren't ectopic, but surplus, if anything. More importantly, the idea that abundance of one or several pro-CO factors might be sufficient to overcome interference would suggest that e.g. COSA-1 overexpression would have the same effect. Haversat (2022) PMID: 35576467 explores this scenario, but it could be that more than one pro-CO factor needs to be stabilized? These points should be addressed in a more comprehensive discussion.

We thank the reviewer for these insightful comments and the opportunity to clarify these important points. Regarding the use of 'ectopic', the reviewer is correct. Our use of the term 'ectopic' was imprecise. We intended to describe additional crossovers that form, likely at sites that would not normally mature into crossovers under wild-type conditions. As suggested, 'surplus' is a more accurate description. We have revised the text accordingly to replace 'ectopic' with 'surplus'. (line 736)

Regarding the relationship between protein abundance and CO interference, we agree with the reviewer that the scenario where an abundance of a single pro-CO factor (e.g., COSA-1 overexpression) can influence interference. Our findings, however, reveal a distinct but potentially complementary mechanism:

First, we propose that the surplus COs in our study of *cosa-1*^{PD} arise not merely from overexpression of a functional protein, but from impaired interactions between pro-CO factors. A strong and stable network of interactions among pro-CO protein is essential for the rapid and orderly progression of the coarsening process. In contrast, weakened protein interactions, such as in *cosa-1*^{PD} mutants, slow the dynamics of coarsening, preventing the system from completing the 'winner-takes-all' selection within the limited temporal window. Consequently, some pro-CO proteins, failing to be fully recruited to the designated CO site, may become stranded at other DSB sites that are normally destined for non-crossover fate, erroneously initiating additional COs. We have clarified this point in the discussion (lines 534-536, 711-725).

Second, As the reviewer noted, and as supported by studies like Haversat et al., the abundance of key factors like COSA-1 is critical. In our study, UPS knockdown leads to the simultaneous stabilization of multiple pro-crossover proteins, positioning the UPS as an upstream hub that coordinates the entire pro-CO network. This offers a new perspective on CO interference regulation: rather than depending solely on the dosage of individual factor, it can be subject to integrated, UPS-mediated control of the entire pro-CO protein

network. Our model thus extends the single-factor dosage concept, proposing that the coordinated stabilization of multiple network components upon UPS inhibition may exert a distinct, collective effect on crossover interference. We have integrated this discussion in the revised manuscript (lines 700-709).

Minor points:

L24: You are not really inhibiting the activity of the UPS, but lowering the expression (via RNAi)

Thank you. We have replaced ‘inhibiting the activity of’ with ‘knockdown of’ in the revised manuscript. The sentence now reads ‘Knockdown of either the ubiquitin-activating enzyme E1 or the proteasome resulted in elevated pro-crossover protein levels and crossover designation.’ (line 24)

L26: replace "interference" with another term such as "impairment". Since the paper is about CO interference, the reader could get confused when the term is used for the depletion of proteins

We thank the reviewer for this suggestion. We have replaced ‘interference’ with ‘impairment’ to avoid confusion The sentence now reads ‘Impairing ubiquitination, but not proteasome activity, led to persistent association of pro-crossover proteins on meiotic chromosomes.’ (lines 26-27)

L38: either parental or homologous, this is too much (between paternal and maternal homologous chromosomes)

We have deleted 'paternal and maternal'.

L56: dynamically associate [no s] - or it could be a sentence fragment that doesn't belong here, in that case please rewrite.

We thank the reviewer for this suggestion. We have rewritten the sentence and it now reads ‘This group of factors, comprising the Zip3 family of proteins, the conserved MutSγ complex COSA-1/CNTD1 and the MutLγ complex, dynamically associate with the chromosomes in a meiosis stage-dependent manner, under the regulation of protein-protein interaction(s).’ (lines 66-71).

L69: that [OMIT the] pro-crossover proteins

We have omitted ‘the’ as suggested.

L86: Didn't Ahuja et al (2017) show that proteasome inhibition, either chemical or via mutation, decreases crossover levels?

We agree with the reviewer that Ahuja et al. (2017) clearly showed a decrease in crossover levels upon proteasome inhibition. Our intended emphasis was on the unresolved biological significance of the dynamic changes in pro-crossover protein localization. To avoid any ambiguity, we have removed the sentence in question.

L94: "leaving the contribution of ubiquitination to pro-crossover factors turnover and crossover patterning remains to be determined" EDIT: Thus, the contribution of ubiquitination to pro-crossover factor[s OMIT] turnover and crossover patterning remains to be determined.

Thank you for correction. We have rephrased the sentence and it now reads 'Thus, the contribution of ubiquitination to pro-crossover factor turnover and crossover patterning remains to be determined.' (lines 105-107)

L161: …are preferentially targeted over other meiotic proteins.

We have revised the sentence and it now reads 'Notably, the level of a GFP-tagged synaptonemal complex component SYP-3 remained unchanged, suggesting that pro-crossover factors might be more preferentially targeted over other meiotic proteins.' (lines 176-179)

L216: further demonstrating that [OMIT the functional] crossover designation remains intact

We have omitted 'functional' (line 242).

L223: focus intensity [foci is plural]

We have replaced 'foci' with 'focus' (line 253).

L267: Univalent were never [REPLACE has never been] observed

We have replaced 'has never been' with 'were never'. And it now reads 'Univalent were never observed under *rpn-6* or *uba-1* knockdown conditions, implying efficient crossover formation.' (lines 302-303)

L273/Fig. 3A,C: Please consider showing the schematics of C.e. germline and proteasome in Fig. 1 where they will be more useful for the general reader.

We thank the reviewer for this suggestion. We have now made schematics of the UPS and *C. elegans* germline and included them at Fig 1A and 1B in the revised figures.

L295: exhibited distinct [INSERT localization] pattern

We have inserted 'localization' as suggested and it now reads 'The remnant foci of COSA-1 and ZHP-3 also exhibited distinct localization pattern, where most foci were in the nucleoplasm of the diakinesis oocytes.' (lines 331-333)

L339: explain the red/black arrows in legend

Sorry, there was no specific intent behind using the two colors; they were simply used to visually distinguish the two pathways. To prevent any potential confusion, we have now unified all arrows to black in the revised Fig 4A.

L363: that [OMIT the] late recombination intermediates

We have omitted 'the' as suggested (line 427).

L365: REPHRASE: as observed for slx-4

We have rephrased the sentence and it now reads 'The persistent association of the pro-crossover factors with meiotic chromosomes observed above could be explained by an alternative possibility that late recombination intermediates were not processed into mature crossovers after *uba-1* or *npl-4* knockdown, as observed for *slx-4* mutants.' (line 429)

L388: It is unclear why HIM-3 is shown when this panel is only about the number of DAPI staining bodies. Unless has to do with staging the cells, but that needs to be explained to a general reader.

Thank you for your comment. We have replaced all the images with those showing DAPI staining only (Fig 5C).

L418: REPLACE [per homolog pair is supposed to be generated] WITH is normally generated

We have replaced 'per homolog pair is supposed to be generated' with 'is normally generated'. (line 495).

L423: Can you provide some significance level for the CO increase. The C.e. reader will know that more than1 CO is rare, but the general reader wonders how different 1 or 2% is from 0%, considering that the number of analyzed events is relatively small.

We thank the reviewer for this important point regarding the statistical significance of the observed CO increase. Given the relatively small sample size and our specific hypothesis that *uba-1* RNAi would increase CO frequency, we employed a one-tailed Fisher's exact test.

Our analysis indicated that in a wild-type background, the increase in CO number following *uba-1* partial RNAi did not reach statistical significance compared with the control RNAi (P=0.065). However, in the $cosa\text{-}1^{PD}$ background, the increase in COs upon *uba-1* partial RNAi was statistically significant compared with the control (P=0.0063). We have now explicitly described this statistical approach and the results in the main text (lines 501-504, 530-533).

L439: impaired instead of attenuated

Thank you. We have replaced 'attenuated' with 'impaired' (lines 519-520).

L613: ref 23 is incorrect in this place, C. elegans was not analyzed in the Rao et al paper.

We have removed the ref 23 from this place in the revised manuscript.

L580-585: Why not start this section with the conclusion, i.e. that protein abundance is linked to CO control, then enumerate the various E3 ligases, in the current order it is unclear why you are enumerating all the various E3 ligase mutant effects.

We sincerely appreciate the reviewer's constructive suggestion regarding the logical organization of this section. Following the recommendation, we have reorganized the content to first present the key finding that protein abundance is intrinsically connected to crossover control. Subsequently, we systematically describe the functional impacts of different E3 ligase mutants (lines 669-676). This revised structure significantly enhances the clarity and coherence of our argument.

L598: The last sentence needs to be integrated into the entire argument, rather than being tagged on

We thank the reviewer for the suggestion. The sentence has been replaced and developed into a more coherent argument: 'Even though the two possibilities are not mutually exclusive, our data suggest that these functions could be separated.' This statement is

now logically preceded by the relevant context and is immediately followed by explanatory sentences that elaborate on its meaning (lines 749-751).
