# Supplementary material for: Ubiquitin-proteasome system regulates pro-crossover protein dynamics during meiosis in Caenorhabditis elegans
Source: PLoS Biol. 2026 Jun 16;24(6):e3003868. doi: 10.1371/journal.pbio.3003868 (PMC13293516; doi:10.1371/journal.pbio.3003868)
Supplement: Attachment — Submitted filename: responses to reviewers 0604.pdf [file pbio.3003868.s019.pdf]

Reviewer remarks:

Black = Referee comments

Red = **Our reply**

Reviewer #1: The authors have addressed all previous concerns. Congratulations on an interesting story.

We are glad that our revisions have fully addressed your previous concerns. Thank you for your time and constructive input throughout the review process.

Reviewer #2: The authors have thoroughly addressed all reviewer comments. While I remain enthusiastic about the findings reported here, there is still quite a list of edits for clarity. Still, a very impressive study.

We truly appreciate your continued enthusiasm for the findings reported in this study, as well as the many detailed suggestions you have provided to improve clarity. Below, please find our point-by-point responses.

L40: Suggest: Meiotic crossover formation is a result of repair of DNA DSBs via homologous recombination

We have rephrased the sentence as suggested. (Lines 38-40)

L60: produced in excess of [OMIT the] crossovers

We have omitted ‘the’.

L61: how future crossover sites are selected from among all [OMIT the] DSBs.

We have omitted ‘the’.

L77: focus number… decreaseS

We have made changes as suggested, and it now reads ‘Their focus number gradually decreases and the remaining foci specifically mark crossover sites’. (line 76)

L117: It is quite difficult to keep focus number and persistence into late stages apart. I suggest that authors remind the reader what stage they are referring to at regular intervals: E.g., "While both ubiquitination and proteasomal degradation control the abundance and chromosomal distribution of pro-crossover factors ALONG PACHYTENE CHROMOSOMES, ubiquitination exclusively governs the dissociation of pro-crossover factors from crossover sites after crossover formation IN DIPLOTENE NUCLEI."

Thank you for the suggestion. We have added stage information into this sentence. Based on our experimental data, the ubiquitination mediated dissociation takes place at the diakinesis stage. We have therefore indicated this in the revised manuscript accordingly and it now reads 'While both ubiquitination and proteasomal degradation control the abundance and chromosomal distribution of pro-crossover factors along pachytene chromosomes, ubiquitination exclusively governs the dissociation of pro-crossover factors from crossover sites after crossover formation in diakinesis nuclei.' (lines 117-119)

L121: of [OMIT the] pro-crossover proteins to ensure accurate crossover patterning, [EDIT] revealING a previously unrecognized restrictive role of the UPS in crossover formation.

We have made changes as suggested, and it now reads 'our findings establish the UPS as a critical post-translational regulator that orchestrates spatiotemporal dynamics of pro-crossover proteins to ensure accurate crossover patterning, revealing a previously unrecognized restrictive role of the UPS in crossover formation.' (lines 120-123)

> L178: might be [OMIT more] preferentially…

We have omitted 'more'.

L223: this enhancement was not associated with [REPLACE did not correspond to] premature appearance of COSA-1 foci

We have replaced 'did not correspond to' with 'was not associated with'. (line 225)

L241: I recommend to mention the atypical remodeling upfront to avoid resistance from the reader "and that remodeling [INSERT though with some deviations from WT] still

occurred following…"

Thank you for this very helpful suggestion. We have revised the paragraph accordingly, now presenting the abnormal morphology first and then stating that remodeling still occurred. Our revised text reads as follows: We found that the knockdown of the UPS did not result in any significant changes in the protein levels of HTP-1 and SYP-1 (S3A to S3C Fig), but diplotene chromosome morphology appears altered, such as shortened or disorganized axes, as indicated by HTP-1 staining (Fig 2B). We speculate that this is related to the previously reported role of the UPS in regulating the dynamics of these proteins [32]. Nevertheless, remodeling still occurred following RPN-6 or UBA-1 depletion (Fig 2B), albeit with some deviations from wild type, further demonstrating that crossover designation remains intact. (lines 241-248)

L264: nuclei with [INSERT the] indicated number

We have inserted 'the'.(line 264)

L333: likely

We have removed 'likely'.

L334 This wording is confusing. I suggest replacing 'foci' with 'aggregates' as foci are a microscopic manifestation and you are describing a cytological structure "reflecting that COSA-1 and ZHP-3 COMPLEXES/ RECOMBINOSOMES could be dissociated from chromosomes but were unable to be degraded."

We agree with the reviewer and have replaced 'foci' with 'complexes'.(line 336)

L335: I find this sentence confusing. Maybe: 'Persistent ... in uba-1, but not rpn-6 was not due to a difference in protein levels, since COSA-1 and ZHP-3 were similarly increased in both mutants.'

Thank you. We have rephrased this sentence as suggested and it now reads 'The persistent chromatin association of ZHP-3 and COSA-1 in *uba-1* RNAi-treated oocyte was not due to a difference in protein levels, since both COSA-1 and ZHP-3 were similarly increased upon *rpn-6* or *uba-1* knockdown.' (lines 337-339)

L344: Say explicitly that you are changing the tag, so the reader doesn't have to sort through the genotypes

Thank you. The sentence now reads ‘We next used GFP-tagged transgenic *cosa-1* and *zhp-3* worms to further validate these findings.’ (line 346)

L357: Again, it would be useful to say what stage you are talking about. "Together, the above data indicate that ubiquitination, but probably not proteasomal degradation, is responsible for the dissociation of [OMIT the] pro-crossover proteins from [DIPLOTENE] chromosomes.

We have made changes accordingly and it now reads ‘Together, the above data indicate that ubiquitination, but probably not proteasomal degradation, is responsible for the dissociation of pro-crossover proteins from diakinesis chromosomes.’ (lines 359-361)

L359-364: I understand that this change was suggested by a reviewer. But in its current form it really disrupts the flow of results. Could you substantially shorten, refer to Discussion and return to this in more detail there?

We agree with the review regarding the flow of results. As suggested, we have shortened this section and only keep one sentence at the end of this paragraph, and moved the details into the Discussion.

And now it reads ‘This mechanism is reminiscent of how polyubiquitination directs the disassembly of stable complexes, such as the replisome (see the Discussion for further details)’ in the Results (lines 361-363);

and ‘A well-established example of ubiquitination-mediated complex disassembly is that of the replisome. In that setting, polyubiquitination of MCM7 (a component of the CMG helicase) primes the replisome for CDC-48$^{UFD-1/NPL-4}$-mediated disassembly at the end of DNA replication [53]. Our findings in this study indicates that polyubiquitin chains on one or more pro-crossover factors may similarly be recognized by CDC-48$^{UFD-1/NPL-4}$ to trigger their dissociation from crossover sites’ in the Discussion (lines 774-780).

L376-386: Please tighten up this section. It is very wordy.

Thank you. We have tightened up this section, and it now reads ‘This is consistent with

the idea that CDC-48 and NPL-4 function within the same pathway. Since *npl-4* RNAi produced a stronger effect than *cdc-48* RNAi (Figs 4C and S5A to S5C), we subsequently used *npl-4* alone to represent the function of the underlying CDC-48UFD−1/NPL−4 segregase complex. We also noticed an evident phenotypic difference between *npl-4* RNAi and *uba-1* RNAi when COSA-1::HA was monitored (20% vs 100%, Figs 3D and 4C). We reason that this likely reflects limitations in detection sensitivity, because when *npl-4* RNAi was performed in transgenic GFP::COSA-1 and ZHP-3::GFP worms, the penetrance of the delayed dissociation phenotype (70%–100%) (S5B and S5C Fig) approached that observed in *uba-1* RNAi (100%) (S4D and S5B Fig).'(lines 374-384)

L398: Rewrite: "Unlike uba-1 RNAi, npl-4 depletion did not RESULT IN an increase in COSA-1 or ZHP-3, even though both cdc-48ufd-1/npl-4 and uba-1 knockdown resulted in delayed dissociation of pro-crossover factors AT DIPLOTENE(?)"

We have rewritten the sentence and it now reads 'Unlike *uba-1* RNAi, *npl-4* depletion did not result in an increase in COSA-1 or ZHP-3 (S5D to S5G Fig), even though both *cdc-48*$^{ufd\text{-}1/npl\text{-}4}$ and *uba-1* knockdown resulted in delayed dissociation of pro-crossover factors at diakinesis.' (lines 386-388)

L392: "specifically regulates the chromosomal association of pro-crossover factors." This wording is confusing. 'Chromosomal association of pro-crossover factors' is ambiguous, as it could refer to either the increased focus number in pachytene nuclei or persistence of foci into post-pachytene stages. See my earlier suggestion.

We have rewritten the sentence and it now reads 'Therefore, in contrast to ubiquitination, CDC-48$^{UFD\text{-}1/NPL\text{-}4}$ specifically regulates the dissociation of pro-crossover factors from diakinesis chromosomes.' (lines 388-390)

L394: segregase is required for STAGE-SPECIFIC dissociation [OMIT of the dissociation] of pro-crossover proteins

We have replaced 'efficient' with 'stage-specific' and omitted 'of the dissociation' as suggested, and it now reads 'Fig 4. The CDC-48$^{UFD\text{-}1/NPL\text{-}4}$ segregase is required for stage-specific dissociation of pro-crossover proteins from chromosomes.'(lines 392-393)

L396: [REPLACE following recognition by WITH] following recognition by

Thank you. The sentence now reads 'Schematic showing the fate of a polyubiquitinated substrate protein with the CDC-48$^{UFD-1/NPL-4}$ segregase.' (lines 393-394)

L409: Make transition smoother: Based on these results...

We have added 'Based on these results,' to the very beginning of this paragraph. (line 407)

L417: Why does that not simply indicate that npl-4 is epistatic to rpn-6, i.e. if you dont displace pro-CO, it doesnt matter whether you have a functioning proteasome?

We thank the reviewer for this insightful comment. We have now modified the relevant sentence to: 'We performed double knockdown of *rpn-6* and *npl-4* and observed persistent chromosome-associated ZHP-3 foci in -1 diakinesis oocytes (Fig 4E and 4F), indicating *npl-4* is epistatic to *rpn-6* in the dissociation of pro-crossover factors.' (lines 415-418)

L419: I recommend to put the alternative explanation of another segregase in a separate, second sentence.

We thank the reviewer for this helpful suggestion. We have split the content into two separate sentences. The revised text now reads 'Collectively, our data establish that the CDC-48$^{UFD-1/NPL-4}$ segregase is required for efficient dissociation of pro-crossover complex from diakinesis chromosome. However, we cannot formally exclude the possibility that another segregase is also involved.' (lines 418-421)

L426: association of [OMIT the] pro-crossover

We have removed 'the'.

L466: Please explain "20% RNAi treatment" for the non-specialist reader. Are you diluting the RNAi with ctrlRNAi or injecting less RNAi?

We apologize for the lack of clarity. By "20% *uba-1* RNAi treatment," we meant that the

bacteria expressing *uba-1* dsRNA was diluted with bacteria carrying the empty vector (control RNAi) at a 1:4 ratio. We have now explained explicitly in the results to make this clear and described how partial RNAi was achieved in the Materials and methods.

It now reads 'After 20% *uba-1* RNAi treatment (diluted 1:4 with control RNAi), we observed partial rescue of bivalent formation defect in *cosa-1*^{PD} mutants, with an average of 6.47 DAPI-stained bodies compared with 8.54 in untreated controls (Figs 5C, 5D and S6A). (lines 466-469)

In Fig. 6A, could you highlight column 6 to indicate that this is the sum of the 5 intervals, and maybe even mention in text? Since the effects of cosa-1PD are non-uniform across intervals, it is difficult for the reader to know what column to focus on.

Thank you for this suggestion. We have highlighted column 6 in red and also mentioned in the figure legend (lines 510-511) and in the text (lines 520-521).

L527: a number never [OMIT been] reached

We have omitted 'been'.

L548: formation [OMIT we discovered] REPORTED here

We have replaced 'we discovered' with 'reported'. (line 550)

L555: we [OMIT thus] compared

We have omitted 'thus'.

L556: with that of UBA-1 FOR their impacts

We have replaced 'in' with 'for'. (line 558)

L571: Split these into two sentences, first your conclusion, then the alternative explanation. Together, while synapsis defects

Thank you for your suggestion. We have split the sentence into two and now it reads 'Together, our genetic evidence strongly suggests that the UPS mediates crossover

control through mechanisms separable from its regulation of SC dynamics. However, we cannot exclude that synapsis defects may still partially contribute to the crossover patterning changes observed upon UPS disruption.' (lines 573-577)

L584: modified proteome was analysed BY MASS SPEC (?)

We have revised this sentence as 'and the ubiquitin-modified proteome was analysed by mass spectrometry' (line 587).

L654: The following phrase is confusing, how can a single CO be widely spaced? "normally ensures a single, widely spaced crossover"

Thank you. We have removed 'widely spaced'. It now reads 'their seemingly random distribution is inconsistent with the robust crossover interference that normally ensures a single crossover per chromosome in *C. elegans*.' (lines 656-657)

L658: Please point the reader to the data that indicate that the double COs are closely spaced

We have updated the sentence to refer to Fig 6B, which provides the supporting data for the close spacing of the double COs (line 661).

L727: Unclear where the "Firstly" is that this "Secondly" refers to.

We thank the reviewer for pointing it out. First and second here refer to two mechanisms, we have proposed, through which UPS regulates crossover patterning. To make it clearer, we have stated the two mechanisms more explicitly, and it now reads: 'Our results demonstrate that the UPS regulates crossover patterning through at least two mechanisms. The first mechanism involves the regulation of pro-crossover protein abundance.' (lines 666-668); and 'The second mechanism by which the UPS regulates crossover patterning involves actively controlling the chromosomal localization of pro-crossover factors.' (lines 731-732)

L741-43 This sentence seems to repeat the previous sentence?

Thank you. To avoid redundancy, we have revised the sentence which now reads 'Consistent with this, the rescue of COSA-1^{PD} accumulation at late pachytene upon

UPS knockdown provides further experimental evidence that the UPS regulates the chromosomal localization of pro-crossover proteins.' (lines 745-747)

L755: Write out "Late pachytene" since this abbreviation hasn't been used earlier\

We have replaced 'LP' with 'late pachytene'. (lines 759-760)

L774: Even though this was listed as corrected in the response to reviews, ref 33 is inappropriate here as it doesn't contain C. elegans data

Thank you. We have removed ref 33 from line 784.

L776: I assume by saying reinforcing you mean 'maintaining CO interference'. Reinforcing is really a confusing term.

Yes, we agree and have replaced 'reinforcing' with 'maintaining'. (line 786)

L993: performed AS [REPLACE according to] previously

We have replaced 'according to' with 'as'. (line 1002)
