# Supplementary material for: Ubiquitin-proteasome system regulates pro-crossover protein dynamics during meiosis in Caenorhabditis elegans
Source: PLoS Biol. 2026 Jun 16;24(6):e3003868. doi: 10.1371/journal.pbio.3003868 (PMC13293516; doi:10.1371/journal.pbio.3003868)
Supplement: Peer review history

# Editor report: Decision Letter 0

**DOI:** 10.1371/journal.pbio.3003868.r001

**Authors:** Richard Hodge

30 Oct 2025

Dear Dr HONG,

Thank you for submitting your manuscript entitled "Ubiquitin-proteasome-mediated regulation of pro-crossover protein dynamics contributes to crossover patterning during C. elegans meiosis" for consideration as a Research Article by PLOS Biology. Please accept my sincere apologies for the long delay in getting back to you as we consulted with an academic editor about your submission.

Your manuscript has now been evaluated by the PLOS Biology editorial staff, as well as by an academic editor with relevant expertise, and I am writing to let you know that we would like to send your submission out for external peer review.

However, before we can send your manuscript to reviewers, we need you to complete your submission by providing the metadata that is required for full assessment. To this end, please login to Editorial Manager where you will find the paper in the 'Submissions Needing Revisions' folder on your homepage. Please click 'Revise Submission' from the Action Links and complete all additional questions in the submission questionnaire.

Once your full submission is complete, your paper will undergo a series of checks in preparation for peer review. After your manuscript has passed the checks it will be sent out for review. To provide the metadata for your submission, please Login to Editorial Manager (https://www.editorialmanager.com/pbiology) within two working days, i.e. by Nov 01 2025 11:59PM.

If your manuscript has been previously peer-reviewed at another journal, PLOS Biology is willing to work with those reviews in order to avoid re-starting the process. Submission of the previous reviews is entirely optional and our ability to use them effectively will depend on the willingness of the previous journal to confirm the content of the reports and share the reviewer identities. Please note that we reserve the right to invite additional reviewers if we consider that additional/independent reviewers are needed, although we aim to avoid this as far as possible. In our experience, working with previous reviews does save time.

If you would like us to consider previous reviewer reports, please edit your cover letter to let us know and include the name of the journal where the work was previously considered and the manuscript ID it was given. In addition, please upload a response to the reviews as a 'Prior Peer Review' file type, which should include the reports in full and a point-by-point reply detailing how you have or plan to address the reviewers' concerns.

During the process of completing your manuscript submission, you will be invited to opt-in to posting your pre-review manuscript as a bioRxiv preprint. Visit http://journals.plos.org/plosbiology/s/preprints for full details. If you consent to posting your current manuscript as a preprint, please upload a single Preprint PDF.

Feel free to email us at plosbiology@plos.org if you have any queries relating to your submission.

Kind regards,

Richard

Richard Hodge, PhD

Senior Editor, PLOS Biology

rhodge@plos.org

PLOS

Empowering researchers to transform science

Carlyle House, Carlyle Road, Cambridge, CB4 3DN, United Kingdom

California (U.S.) corporation #C2354500, based in San Francisco

# Peer review: Decision Letter 1

**DOI:** 10.1371/journal.pbio.3003868.r002

**Authors:** Richard Hodge, Richard Hodge

5 Jan 2026

Dear Dr HONG,

Thank you for your patience while your manuscript "Ubiquitin-proteasome-mediated regulation of pro-crossover protein dynamics contributes to crossover patterning during C. elegans meiosis" was peer-reviewed at PLOS Biology. Please accept my sincere apologies for the delays that you have experienced during the peer review process. Your manuscript has now been evaluated by the PLOS Biology editors, an Academic Editor with relevant expertise, and by two independent reviewers.

In light of the reviews, which you will find at the end of this email, we would like to invite you to revise the work to thoroughly address the reviewers' reports.

As you will see, both reviewers are generally positive about the study and think it is interesting and that the data are of high quality. However, the reviewers raise overlapping concerns about the interpretation and explanation of the data in several places and with the lack of important quantifications and missing controls to fully support the findings. In addition, Reviewer #2 notes that the discussion section should be expanded to include a more comprehensive consideration of the results.

Given the extent of revision needed, we cannot make a decision about publication until we have seen the revised manuscript and your response to the reviewers' comments. Your revised manuscript is likely to be sent for further evaluation by all or a subset of the reviewers.

In addition to these revisions, you will need to complete some formatting changes, which you will receive in a follow up email. A member of our team will be in touch with a set of requests shortly.

We expect to receive your revised manuscript within 3 months. Please email us (plosbiology@plos.org) if you have any questions or concerns, or would like to request an extension.

At this stage, your manuscript remains formally under active consideration at our journal; please notify us by email if you do not intend to submit a revision so that we may withdraw it.

**IMPORTANT - SUBMITTING YOUR REVISION**

Your revisions should address the specific points made by each reviewer. Please submit the following files along with your revised manuscript:

1. A 'Response to Reviewers' file - this should detail your responses to the editorial requests, present a point-by-point response to all of the reviewers' comments, and indicate the changes made to the manuscript.

*NOTE: In your point-by-point response to the reviewers, please provide the full context of each review. Do not selectively quote paragraphs or sentences to reply to. The entire set of reviewer comments should be present in full and each specific point should be responded to individually, point by point.

You should also cite any additional relevant literature that has been published since the original submission and mention any additional citations in your response.

2. In addition to a clean copy of the manuscript, please also upload a 'track-changes' version of your manuscript that specifies the edits made. This should be uploaded as a "Revised Article with Changes Highlighted" file type.

*Re-submission Checklist*

When you are ready to resubmit your revised manuscript, please refer to this re-submission checklist: https://plos.io/Biology_Checklist

To submit a revised version of your manuscript, please go to https://www.editorialmanager.com/pbiology/ and log in as an Author. Click the link labelled 'Submissions Needing Revision' where you will find your submission record.

Please make sure to read the following important policies and guidelines while preparing your revision:

*Published Peer Review*

Please note while forming your response, if your article is accepted, you may have the opportunity to make the peer review history publicly available. The record will include editor decision letters (with reviews) and your responses to reviewer comments. If eligible, we will contact you to opt in or out. Please see here for more details:

https://blogs.plos.org/plos/2019/05/plos-journals-now-open-for-published-peer-review/

*PLOS Data Policy*

Please note that as a condition of publication PLOS' data policy (http://journals.plos.org/plosbiology/s/data-availability) requires that you make available all data used to draw the conclusions arrived at in your manuscript. If you have not already done so, you must include any data used in your manuscript either in appropriate repositories, within the body of the manuscript, or as supporting information (N.B. this includes any numerical values that were used to generate graphs, histograms etc.). For an example see here: http://www.plosbiology.org/article/info%3Adoi%2F10.1371%2Fjournal.pbio.1001908#s5

*Blot and Gel Data Policy*

We require the original, uncropped and minimally adjusted images supporting all blot and gel results reported in an article's figures or Supporting Information files. We will require these files before a manuscript can be accepted so please prepare them now, if you have not already uploaded them. Please carefully read our guidelines for how to prepare and upload this data: https://journals.plos.org/plosbiology/s/figures#loc-blot-and-gel-reporting-requirements

*Protocols deposition*

To enhance the reproducibility of your results, we recommend that if applicable you deposit your laboratory protocols in protocols.io, where a protocol can be assigned its own identifier (DOI) such that it can be cited independently in the future. Additionally, PLOS ONE offers an option for publishing peer-reviewed Lab Protocol articles, which describe protocols hosted on protocols.io. Read more information on sharing protocols at https://plos.org/protocols?utm_medium=editorial-email&utm_source=authorletters&utm_campaign=protocols

Thank you again for your submission to our journal. We hope that our editorial process has been constructive thus far, and we welcome your feedback at any time. Please don't hesitate to contact us if you have any questions or comments.

Best regards,

Richard

Richard Hodge, PhD

Senior Editor, PLOS Biology

rhodge@plos.org

------------------------------------

REVIEWS:

Reviewer #1: The manuscript by Zhang et al. investigates how the ubiquitin-proteasome system (UPS) controls the behavior of pro-crossover proteins during C. elegans meiosis. Using a combination of Data-Independent Acquisition proteomics, RNAi screens, and cytological analysis, the authors show that inhibiting the E1 enzyme UBA-1 or the proteasome increases levels of pro-crossover proteins and elevates crossover designation. Interestingly, blocking ubiquitination, but not the proteasome activity, causes pro-crossover proteins to persist on meiotic chromosomes, and their removal requires the CDC-48 segregase complex. Using a hypomorphic allele of cosa-1, the authors further show that partial uba-1 knockdown rescues crossover formation, supporting a model in which the UPS restricts crossover number by tuning pro-crossover protein levels and ensuring their timely dissociation from chromatin.

This study addresses an important question in meiotic regulation, and the identification of pro-crossover factors as UPS targets is very interesting. The experimental approaches are largely appropriate and the data are of high quality. However, there are several concerns regarding the interpretation of the results, the statistical power of the proteomics data, and the lack of evidence demonstrating RNAi efficiency (particularly for the proteasome core). Addressing these points and incorporating the suggested quantifications and explanations would substantially strengthen the manuscript.

Main points:

Figure 1: Statistical Power of Proteomics

The Methods and Figure 7 legend indicate that the DIA proteomics relied on N = 2 biological replicates. N = 2 yields very poor statistical power and likely results in a large False Discovery Rate (FDR) for many proteins. For a study relying on this data to define the mechanism, this is below the standard for the field. Increasing the biological replicate number is highly recommended. If this is not possible, the authors must provide a strong justification in the text (e.g., extreme rarity of samples) and be more transparent about the statistical limitations.

Figure 2B: If altering levels of pro-CO factors by uba-1 RNAi induces double crossovers as shown in Figure 6, this should result in ring-like bivalents. Have the authors observed such structures?

SYP-1 appears abnormal in RNAi-treated animals, as it is continuous between two chromosomes in rpn-6 RNAi-treated oocytes, and there more additional smaller SYP-1 foci in uba-1 RNAi-treated oocytes. Can the authors comment about this?

Figure 2D: The authors observe MSH-5 foci that lack COSA-1 and interpret this as a failure of the UPS to "dismantle recombination intermediates". Could it be that the persistent MSH-5 foci represent excess protein aggregates that cannot be degraded, rather than bona fide recombination intermediates? Perhaps showing additional recombination marker (e.g. RMH-1) would resolve this issue.

Figure 3D-E: Missing controls for pbs-6 RNAi

A key claim is that ubiquitination (uba-1), but not the proteasome core (pbs-6), is required for the dissociation of pro-crossover proteins (Figure 3D). However, the authors do not provide evidence that the pbs-6 RNAi was effective. Without a Western blot or IF demonstrating that pbs-6 RNAi caused a global accumulation of COSA-1/ZHP-3 similar to rpn-6 RNAi, the result is uninterpretable.

After the paragraph ending at line 315 before leading up to the next section, it would be helpful to conclude the results with potential explanations. I suggest the authors to lay out both possibilities up front: (1) the involvement of CDC-48 and (2) the necessity of ubiquitination for processing DNA substrates (e.g. resolution).

Figure 4: Penetrance of cdc-48 RNAi knockdown

The text states that CDC-48 is "mediated" and "responsible for" dissociation. However, the data in Fig 4C show that only a small fraction (~15-20%) of -1 oocytes retain foci upon cdc-48 knockdown. This low penetrance does not support the claim that CDC-48 is the primary segregase for this process. I suggest that the authors acknowledge this issue and compare the cdc-48 RNAi phenotype with uba-1 RNAi to evaluate to what extent they phenocopy each other.

Minor points:

Figure 1A: The volcano plot highlights BRD-1 alongside known pro-crossover factors. However, the text does not explain its relevance. Please briefly mention BRD-1's known or suspected connection to meiosis to justify its inclusion in the figure.

Figure 1B: Since the experiment tested hundreds of proteins, it would be more appropriate to report both FDR and p-values for the highlighted hits.

Figure 1D-F: Please add small, high-magnification insets of representative nuclei for different meiotic stages for the rpn-6 and uba-1 RNAi conditions to clearer show the localization of mis-regulated COSA-1 and ZHP-3.

Figure 3: Please add a quantification panel scoring the percentage of -1/-2/-3 oocytes with chromosome-associated foci across control, uba-1, rpn-6, and pbs-6 RNAi to allow for direct comparison.

Line 55: Please define the ZMM proteins. It appears that this term is used distinctively from pro-crossover factors. If ZMM proteins are broadly defined as proteins that stabilize crossover-specific recombination intermediates, CDK-2 and COSA-1/CNTD1 can certainly be classified as the ZMM proteins.

Line 64: Reference 19 is not published in Nature Cell Biology (https://doi.org/10.1038/s41556-025-01688-9)

Line 70: It looks like the abbreviation for non-crossover (NCO) is not used throughout the manuscript. I suggest to remove it.

Reviewer #2: In their manuscript "Ubiquitin-proteasome-mediated regulation of pro-crossover protein dynamics contributes to crossover patterning during C. elegans meiosis", Drs. Zhang, Liang, Li. and colleagues explore how meiotic recombination is affected by defective degradation via the ubiquitin-proteasome system. Analzying consequences of depletion of E1(Uba-1)-mediated ubiquitin activation and of proteasome RP and CP components, they show that RNAi-mediated depletion of these essential components has several intriguing effects: First, crossover-associated proteins such as COSA-1 and MSH-5 form foci in excess of those normally observed in C. elegans, even though other meiotic chromosome components are not affected in the same way. Second, rather than disappearing at the end of pachynema, foci tend to persist into later stages of meiosis, diplonema or even diakinesis. Third, COSA-1 abundance is increased in the nucleoplasm of cells throughout meiosis. Fourth, instead of forming one crossover per homologue pair, both cytological and importantly also genetic analysis indicates that additional crossovers are formed frequently. The work further explores whether persistence of CO-proteins on meiotic chromosomes is related to a failure to displace them via Cdc48.

Overall, this is an intiguing piece of research. It gives rise to the idea that simply the failure to degrade procrossover factors is sufficient to keep them in place along chromosomes, increase their localization to additional sites and, most surprisingly, result in the formation of additional crossovers, essentially overriding mechanisms that limit the frequency of crossovers. This is quite a surprising finding, as it suggests a critical role of the UPS system in limiting crossovers, above and beyond of just targeting components of the recombination machinery. Moreover, limiting the availability of CO proteins might be a key determinant for CO numbers.

The data quality and analyses are quite impressive. My suggestions for revisions are mostly textual, even though some additional analysis of existing data may also be required. Overall, the Discussion is rather superficial and lacks detailed consideration of the rather complicated set of results.

Major points:

L47: "The mechanism underlying crossover interference remains elusive." - This statement really understates the status of current research. There are various models out there, among them the coarsening that is brough up later in the manuscript, but also the stress hypothesis, involvement of the SC or not, role of topo2 and the UPS system. Rather than elaborating too much on class II crossovers which, as the authors state, likely do not exist in their experimental system, they should at least paraphrase the current status of insights.

L48: DSBs have not been introduced.

L125: The GO annotation is so redundant that it is simply not informative. There is "cell cycle", "cell cycle regulation", "mitotic cell cycle"-this all seems to refer to the same thing. Can you do Revigo or other analysis that reduces redundancies? I understand that you are not using the data, but it will make things more useful for the general reader.

L130: REPLACE "was not identified" with "detected"

Fig. 1D: It needs to be mentioned in the text that this staining is initially nucleoplasmic and later transitions to foci along chromosomes. I suggest showing an excerpt of the region where uba-1 and rpn-6 exhibit the transiton from excessive nucleoplasmic COSA-1 or ZHP-3 staining to foci. (Either here or in Fig. 2A where this is indicated by a (barely visible) dashed white line.)

L142/Fig. 1E: Unclear whether this quantitation was done for entire germ line. Also, please provide the number of germlines analyzed.

L186: I would show the spo-11 result in the main figure. Also, please state clearly whether there are any foci detected in spo-11 or only staining in the nucleoplasm

L197: The section on MPK-1 needs a conclusion to be useful, e.g., increased COSA-1 abundance is not an indirect consequence of progression defect. It is not clear to this reviewer whether there is a progression defect in uba-1 or rpn-6. Apparently not, but why then introduce mpk-1?

L205: "COSA-1 accumulation" is very unspecific- do you mean additional foci and/or more intense foci and/or nucleoplasmic staining?

L224//Fig. 2A: The spatial patterning of nuclei in straight lines in the two mutant germlines, but not the WT, looks unusual. It should be clearly stated whether this is part of the mutant phenotype.

Fig. 2B: While the SC/axis differentaition appear to be intact in the UPS mutrants, the morphology of diplotene nuclei looks quite different (as would be expected if there are additional crossovers). Can you quantitate the aberrant morphologies?

Fig. 2C/L242/L247: Please quantitate how many MSH-5 foci without COSA-1 and vice versa. This is important for a mechanistic understanding: Is MSH-5 persisting and COSA-1 following suit or are both persisting coordinately. This should also be addressed in the Discussion.

Discussion: I understand that in C.e., it is widely considered as a loss of CO ionterference if there are 2COs or 3COs on a given chromosome. But in the current case, it seems a reasonable alterantive that a defective UPS simply results in more COs. Whether these crossovers still interfere with each other would depend on coefficient-of-coindicence analysis, which is difficult with this small data set. The authors should address this point at the very lest in the Discussion.

L273/Fig. 3: The argument that the E1 ligase is more critical than the proteasome is the least convincing part of the paper. Isnt it possible that the E1 ligase is more limiting, and hence the knockdown effects are more dramatic and/or lasting into later cellular stages compared to the proteasome subunit knockdown which still allows many fully functional proteasome particles to be assembled.

L288: undetectable and completely absent are the same thing.

L293: So not all foci disappear? How many remain, in how many diakinesis oocytes?

L314-L332: I understand that the authors want to address the point whether there is a pathway of polyubiquitination>displacement by Cdc48>degradation by proteasome, but the analysis is not very rigorous, and the rational for double mutants should be better explained, to support or exclude this scenario. If this scenario was the case, shouldn't there be just nucleoplasmic foci in the proteasome mutant, rather than exhibiting a longer persistence on chromosomes?

L353: This section is confusing: Fig. 3B shows that rpn-6 results in persistence of ZHP-3 and COSA-1, but the statement in the text claims that it doesn impair the dissoiciation of these factors. If you mean that it retains pro-CO factors during diplotene, but remains functional for dissociation during diakinesis, this needs to be said clearly. Also, the reader needs to see the rpn-6 single mutant (again) in this figure, and the entire germline to get a sense whether the increased levels of COSA-1 in the nucleoplasm are part of the phenotype in the double mutant.

L368: I couldnt find the cosa-1-PD allele in your NAR 2024 paper - is it mentioned under a different name? Also, the rationale/approach for identifying effects of these two aminoacids needs to be explained, and how these aas are mutated (deleted, replaced by Ala?).

Fig. 5I/L393: Please add the rpn-6 and uba-1 single mutants to the panel so the reader can compare the double and single mutants side-by-side

L401-405: The rescue of the binding-impaired COSA-1 by reducing the UPS machinery is quite impressive, but the section is difficult to follow, and the authors need to do a better job in explaining their rational and findings here. Either decreased ubiquitination or decreased displacement via Cdc48 rescue, but is it unclear why rpn-6 is only shown for COSA-1 foci, but not for DAPI staining bodies.

L463: "Consistent with its defect in crossover formation, cosa-1PD exhibited a reduced recombination rate" - I'm not sure what this sentence is trying to say. Crossover formation and recombination rate in this context are the same thing, so the statement sounds rather tautological. Maybe you mean: As expected based on effects on COSA-1 focus formation and viability/HIM effects, crossover frequencies are reduced?

L446: I do not see any nuclei with >9 COSA-1 foci in cosa-1PD in Fig. 5I, so the argument makes no sense

L450: "Analysis of double and triple crossover positioning revealed no consistent spacing preference, indicating a fundamental breakdown of crossover spacing constraints following UBA-1 depletion (Fig 6B)." - The fact that only 10 events were analyed should be added as a caveat here. Also, as mentioned before, it is unclear to this reader whether we are looking at a breakdown of CO spacing or simply additional events that mature into COs. This alternative explanation should be discussed.

L482: the crossover formation defect was EXAGGERTED [REPLACE deteriorated] as SUGGESTED [revealed] by the increased incidence of univalentS [INSERT]

L564: UPS's DUAL [REPLACE bidirectional] functionality

L572: The authors should provide a better rational for the MPK-1 experiment or maybe remove it.

L602: Coarsening was never explained in the Introduction and the relevant references need to be provided (e.g. Morgan 2021 and others).

L604: Are these additional crossovers really ectopic? Isnt the results simply that some additional DSBs mature into COs - they arent ectopic, but surplus, if anything. More importantly, the idea that abundance of one or several pro-CO factors might be sufficient to overcome interference would suggest that e.g. COSA-1 overexpression would have the same effect. Haversat (2022) PMID: 35576467 explores this scenario, but it could be that more than one pro-CO factor needs to be stabilized? These points should be addressed in a more comprehensive discussion.

Minor points:

L24: You are not really inhibiting the activity of the UPS, but lowering the expression (via RNAi)

L26: replace "interference" with anothter term such as "impairment". Since the paper is about CO interference, the reader could get confused when the term is used for the depletion of proteins

L38: either parental or homologous, this is too much (between paternal and maternal homologous chromosomes)

L56: dynamically associate [no s] - or it could be a sentence fragment that doesn't belong here, in that case please rewrite.

L69: that [OMIT the] pro-crossover proteins

L86: Didn't Ahuja et al (2017) show that proteasome inhibiton, either chemical or via mutation, decreases crossover levels?

L94: "leaving the contribution of ubiquitination to pro-crossover factors turnover and crossover patterning remains to be determined" EDIT: Thus, the contribution of ubiquitination to pro-crossover factor[s OMIT] turnover and crossover patterning remains to be determined.

L161: …are preferentially targeted over other meiotic proteins.

L216: further demonstrating that [OMIT the functional] crossover designation remains intact

L223: focus intensity [foci is plural]

L267: Univalent were never [REPLACE has never been] observed

L273/Fig. 3A,C: Please consider showing the schematics of C.e. germline and proteasome in Fig. 1 where they will be more useful for the general reader.

L295: exhibited distinct [INSERT localization] pattern

L339: explain the red/black arrows in legend

L363: that [OMIT the] late recombination intermediates

L365: REPHRASE: as observed for slx-4

L388: It is unclear why HIM-3 is shown when this panel is only about the number of DAPI staining bodies. Unless has to do with staging the cells, but that needs to be explained to a general reader.

L418: REPLACE [per homolog pair is supposed to be generated] WITH is normally generated

L423: Can you provide some significance level for the CO increase. The C.e. reader will know that more than1 CO is rare, but the general reader wonders how different 1 or 2% is from 0%, considering that the number of analyzed events is relatively small.

L439: impaired instead of attenuated

L613: ref 23 is incorrect in this place, C. elegans was not analyed in the Rao et al paper.

L580-585: Why not start this section with the conclusion, i.e.that protein abundance is linked to CO control, then enumerate the various E3 ligases, in the current order its unclear why you are enumerating all the various E3 ligase mutant effects.

L598: The last sentence needs to be integrated into the entire argument, rather than being tagged on

# Author comment: Author response to Decision Letter 2

**DOI:** 10.1371/journal.pbio.3003868.r003

6 Apr 2026

Attachment Submitted filename: PB-revision-responses to reviewers.pdf

# Peer review: Decision Letter 2

**DOI:** 10.1371/journal.pbio.3003868.r004

**Authors:** Richard Hodge, Richard Hodge, Richard Hodge

22 May 2026

Dear Dr HONG,

Thank you for your patience while we considered your revised manuscript "Ubiquitin-proteasome-mediated regulation of pro-crossover protein dynamics contributes to crossover patterning during C. elegans meiosis" for publication as a Research Article at PLOS Biology. Please accept my sincere apologies for the delays that you have experienced during this round of the peer review process. This revised version of your manuscript has been evaluated by the PLOS Biology editors, the Academic Editor and the original reviewers.

Based on the reviews, I am pleased to say that we are likely to accept this manuscript for publication, provided you satisfactorily address the remaining points raised by Reviewer #2. Please also make sure to address the following data and other policy-related requests that I have provided below (A-F):

(A) We routinely suggest changes to titles to ensure maximum accessibility for a broad, non-specialist readership. In this case, we would suggest a minor edit to the title, as follows. Please ensure you change both the manuscript file and the online submission system, as they need to match for final acceptance:

“Ubiquitin-proteasome system regulates pro-crossover protein dynamics during meiosis in C. elegans”

(B) You may be aware of the PLOS Data Policy, which requires that all data be made available without restriction: http://journals.plos.org/plosbiology/s/data-availability. For more information, please also see this editorial: http://dx.doi.org/10.1371/journal.pbio.1001797

Note that we do not require all raw data. Rather, we ask that all individual quantitative observations that underlie the data summarized in the figures and results of your paper be made available in one of the following forms:

-Supplementary files (e.g., excel). Please ensure that all data files are uploaded as 'Supporting Information' and are invariably referred to (in the manuscript, figure legends, and the Description field when uploading your files) using the following format verbatim: S1 Data, S2 Data, etc. Multiple panels of a single or even several figures can be included as multiple sheets in one excel file that is saved using exactly the following convention: S1_Data.xlsx (using an underscore).

-Deposition in a publicly available repository. Please also provide the accession code or a reviewer link so that we may view your data before publication.

Regardless of the method selected, please ensure that you provide the individual numerical values that underlie the summary data displayed in the following figure panels as they are essential for readers to assess your analysis and to reproduce it:

Figure 1C, 1G, 1I, 1K, 2F, 3D, 4C, 4E, 5D-G, 5I, 7A-B, S1A-B, S1F, S2C, S3B-E, S4C-D, S5B-C, S5E, S5G, S6C, S8D, S8H

NOTE: the numerical data provided should include all replicates AND the way in which the plotted mean and errors were derived (it should not present only the mean/average values).

(C) Please also ensure that each of the relevant figure legends in your manuscript include information on *WHERE THE UNDERLYING DATA CAN BE FOUND*, and ensure your supplemental data file/s has a legend.

(D) Per journal policy, if you have generated any custom code during the course of this investigation, please make it available without restrictions. Please ensure that the code is sufficiently well documented and reusable, and that your Data Statement in the Editorial Manager submission system accurately describes where your code can be found. More information on our Code Policy, what and how to share can be found here: https://journals.plos.org/plosbiology/s/code-availability

Please note that we cannot accept sole deposition of code in GitHub, as this could be changed after publication. However, you can archive this version of your publicly available GitHub code to Zenodo. Once you do this, it will generate a DOI number, which you will need to provide in the Data Accessibility Statement (you are welcome to also provide the GitHub access information). See the process for doing this here: https://docs.github.com/en/repositories/archiving-a-github-repository/referencing-and-citing-content

(E) Please ensure that you are using best practice for statistical reporting and data presentation. These are our guidelines https://journals.plos.org/plosbiology/s/best-practices-in-research-reporting#loc-statistical-reporting and a useful resource on data presentation https://journals.plos.org/plosbiology/article?id=10.1371/journal.pbio.1002128

- If you are reporting experiments where n ≤ 5, please plot each individual data point.

(F) Please note that per journal policy, the model system/species studied should be clearly stated in the abstract of your manuscript.

------------------------------------------------------------------------

As you address these items, please take this last chance to review your reference list to ensure that it is complete and correct. If you have cited papers that have been retracted, please include the rationale for doing so in the manuscript text, or remove these references and replace them with relevant current references. Any changes to the reference list should be mentioned in the cover letter that accompanies your revised manuscript.

In addition to these revisions, you may need to complete some formatting changes, which you will receive in a follow up email. A member of our team will be in touch with a set of requests shortly. If you do not receive a separate email within a few days, please assume that checks have been completed, and no additional changes are required.

We expect to receive your revised manuscript within two weeks.

To submit your revision, please go to https://www.editorialmanager.com/pbiology/ and log in as an Author. Click the link labelled 'Submissions Needing Revision' to find your submission record. Your revised submission must include the following:

- a cover letter that should detail your responses to any editorial requests, if applicable, and whether changes have been made to the reference list

- a Response to Reviewers file that provides a detailed response to the reviewers' comments (if applicable, if not applicable please do not delete your existing 'Response to Reviewers' file.)

- a track-changes file indicating any changes that you have made to the manuscript.

NOTE: If Supporting Information files are included with your article, note that these are not copyedited and will be published as they are submitted. Please ensure that these files are legible and of high quality (at least 300 dpi) in an easily accessible file format. For this reason, please be aware that any references listed in an SI file will not be indexed. For more information, see our Supporting Information guidelines:

https://journals.plos.org/plosbiology/s/supporting-information

*Published Peer Review History*

Please note that you may have the opportunity to make the peer review history publicly available. The record will include editor decision letters (with reviews) and your responses to reviewer comments. If eligible, we will contact you to opt in or out. Please see here for more details:

https://plos.org/published-peer-review-history/

*Press*

Should you, your institution's press office or the journal office choose to press release your paper, please ensure you have opted out of Early Article Posting on the submission form. We ask that you notify us as soon as possible if you or your institution is planning to press release the article.

*Protocols deposition*

To enhance the reproducibility of your results, we recommend that if applicable you deposit your laboratory protocols in protocols.io, where a protocol can be assigned its own identifier (DOI) such that it can be cited independently in the future. Additionally, PLOS ONE offers an option for publishing peer-reviewed Lab Protocol articles, which describe protocols hosted on protocols.io. Read more information on sharing protocols at https://plos.org/protocols?utm_medium=editorial-email&utm_source=authorletters&utm_campaign=protocols

Please do not hesitate to contact me should you have any questions.

Best regards,

Richard

Richard Hodge, PhD

Senior Editor, PLOS Biology

rhodge@plos.org

------------------------------------------------------------------------

Reviewer remarks:

Reviewer #1: The authors have addressed all previous concerns. Congratulations on an interesting story.

Reviewer #2: The authors have thoroughly addressed all reviewer comments. While I remain enthusiastic about the findings reported here, there is still quite a list of edits for clarity. Still, a very impressive study.

L40: Suggest: Meiotic crossover formation is a result of repair of DNA DSBs via homologous recombination

L60: produced in excess of [OMIT the] crossovers

L61: how future crossover sites are selected from among all [OMIT the] DSBs.

L77: focus number… decreaseS

L117: It is quite difficult to keep focus number and persistence into late stages apart. I suggest that authors remind the reader what stage they are referring to at regular intervals: E.g., "While both ubiquitination and proteasomal degradation control the abundance and chromosomal distribution of pro-crossover factors ALONG PACHYTENE CHROMOSOMES, ubiquitination exclusively governs the dissociation of pro-crossover factors from crossover sites after crossover formation IN DIPLOTENE NUCLEI."

L121: of [OMIT the] pro-crossover proteins to ensure accurate crossover patterning, [EDIT] revealING a previously unrecognized restrictive role of the UPS in crossover formation.

L178: might be [OMIT more] preferentially…

L223: this enhancement was not associated with [REPLACE did not correspond to] premature appearance of COSA-1 foci

L241: I recommend to mention the atypical remodeling upfront to avoid resistance from the reader "and that remodeling [INSERT though with some deviations from WT] still occurred following…"

L264: nuclei with [INSERT the] indicated number

L333: likely

L334 This wording is confusing. I suggest replacing 'foci' with 'aggregates' as foci are a microscopic manifestation and you are describing a cytological structure "reflecting that COSA-1 and ZHP-3 COMPLEXES/ RECOMBINOSOMES could be dissociated from chromosomes but were unable to be degraded."

L335: I find this sentence confusing. Maybe: 'Persistent ... in uba-1, but not rpn-6 was not due to a difference in protein levels, since COSA-1 and ZHP-3 were similarly increased in both mutants.'

L344: Say explicitly that you are changing the tag, so the reader doesn't have to sort through the genotypes

L357: Again, it would be useful to say what stage you are talking about. "Together, the above data indicate that ubiquitination, but probably not proteasomal degradation, is responsible for the dissociation of [OMIT the] pro-crossover proteins from [DIPLOTENE] chromosomes.

L359-364: I understand that this change was suggested by a reviewer. But in its current form it really disrupts the flow of results. Could you substantially shorten, refer to Discussion and return to this in more detail there?

L376-386: Please tighten up this section. It is very wordy.

L398: Rewrite: "Unlike uba-1 RNAi, npl-4 depletion did not RESULT IN an increase in COSA-1 or ZHP-3, even though both cdc-48ufd-1/npl-4 and uba-1 knockdown resulted in delayed dissociation of pro-crossover factors AT DIPLOTENE(?)"

L392: "specifically regulates the chromosomal association of pro-crossover factors." This wording is confusing. 'Chromosomal association of pro-crossover factors' is ambiguous, as it could refer to either the increased focus number in pachytene nuclei or persistence of foci into post-pachytene stages. See my earlier suggestion.

L394: segregase is required for STAGE-SPECIFIC dissociation [OMIT of the dissociation] of pro-crossover proteins

L396: [REPLACE following recognition by WITH] following recognition by

L409: Make transition smoother: Based on these results...

L417: Why does that not simply indicate that npl-4 is epistatic to rpn-6, i.e. if you dont displace pro-CO, it doesnt matter whether you have a functioning proteasome?

L419: I recommend to put the alternative explanation of another segregase in a separate, second sentence.

L426: association of [OMIT the] pro-crossover

L466: Please explain "20% RNAi treatment" for the non-specialist reader. Are you diluting the RNAi with ctrlRNAi or injecting less RNAi?

In Fig. 6A, could you highlight column 6 to indicate that this is the sum of the 5 intervals, and maybe even mention in text? Since the effects of cosa-1PD are non-uniform across intervals, it is difficult for the reader to know what column to focus on.

L527: a number never [OMIT been] reached

L548: formation [OMIT we discovered] REPORTED here

L555: we [OMIT thus] compared

L556: with that of UBA-1 FOR their impacts

L571: Split these into two sentences, first your conclusion, then the alternative explanation. Together, while synapsis defects

L584: modified proteome was analysed BY MASS SPEC (?)

L654: The following phrase is confusing, how can a single CO be widely spaced? "normally ensures a single, widely spaced crossover"

L658: Please point the reader to the data that indicate that the double COs are closely spaced

L727: Unclear where the "Firstly" is that this "Secondly" refers to.

L741-43 This sentence seems to repeat the previous sentence?

L755: Write out "Late pachytene" since this abbreviation hasn't been used earlier\

L774: Even though this was listed as corrected in the response to reviews, ref 33 is inappropriate here as it doesn't contain C. elegans data

L776: I assume by saying reinforcing you mean 'maintaining CO interference'. Reinforcing is really a confusing term.

L993: performed AS [REPLACE according to] previously

# Author comment: Author response to Decision Letter 3

**DOI:** 10.1371/journal.pbio.3003868.r005

4 Jun 2026

Attachment Submitted filename: responses to reviewers 0604.pdf

# Editor report: Decision Letter 3

**DOI:** 10.1371/journal.pbio.3003868.r006

**Authors:** Richard Hodge, Richard Hodge, Richard Hodge, Richard Hodge

5 Jun 2026

Dear Dr Hong,

On behalf of my colleagues and the Academic Editor, Sarah Zanders, I am pleased to say that we can accept your manuscript for publication, provided you address any remaining formatting and reporting issues. These will be detailed in an email you should receive within 2-3 business days from our colleagues in the journal operations team; no action is required from you until then. Please note that we will not be able to formally accept your manuscript and schedule it for publication until you have completed any requested changes.

Please take a minute to log into Editorial Manager at http://www.editorialmanager.com/pbiology/, click the "Update My Information" link at the top of the page, and update your user information to ensure an efficient production process.

PRESS

We frequently collaborate with press offices. If your institution or institutions have a press office, please notify them about your upcoming paper at this point, to enable them to help maximise its impact. If the press office is planning to promote your findings, we would be grateful if they could coordinate with biologypress@plos.org. If you have previously opted in to the early version process, we ask that you notify us immediately of any press plans so that we may opt out on your behalf.

We also ask that you take this opportunity to read our Embargo Policy regarding the discussion, promotion and media coverage of work that is yet to be published by PLOS. As your manuscript is not yet published, it is bound by the conditions of our Embargo Policy. Please be aware that this policy is in place both to ensure that any press coverage of your article is fully substantiated and to provide a direct link between such coverage and the published work. For full details of our Embargo Policy, please visit http://www.plos.org/about/media-inquiries/embargo-policy/.

Thank you again for choosing PLOS Biology for publication and supporting Open Access publishing. We look forward to publishing your study.

Best wishes,

Richard

Richard Hodge, PhD

Senior Editor, PLOS Biology

rhodge@plos.org

PLOS

Empowering researchers to transform science

Carlyle House, Carlyle Road, Cambridge, CB4 3DN, United Kingdom

California (U.S.) corporation #C2354500, based in San Francisco
